# Evaluating Journal Impact Factor: a systematic survey of the pros and cons, and overview of alternative measures

**DOI:** 10.1590/1678-9199-JVATITD-2019-0082

**Published:** 2020-08-31

**Authors:** Eugene Mech, Muhammad Muneeb Ahmed, Edward Tamale, Matthew Holek, Guowei Li, Lehana Thabane

**Affiliations:** 1 Department of Biochemistry and Biomedical Sciences, McMaster University, Hamilton, ON, Canada.; 2 Department of Health Research Methods, Evidence, and Impact (HEI), McMaster University, Hamilton, ON, Canada.; 3 Michael G. DeGroote School of Medicine, McMaster University, Hamilton, ON, Canada.; 4 Center for Clinical Epidemiology and Methodology (CCEM), Guangdong Second Provincial General Hospital, China.; 5 Biostatistics Unit, Research Institute at St Joseph’s Healthcare, Hamilton, ON, Canada.; 6 Department of Anesthesia, McMaster University, Hamilton, ON, Canada.; 7 Department of Pediatrics, McMaster University, Hamilton, ON, Canada.; 8 School of Nursing, McMaster University, Hamilton, ON, Canada.; 9 School of Rehabilitation Science, McMaster University, Hamilton, ON, Canada.

**Keywords:** Journal Impact Factor, Bibliometrics, Alternative metrics, Citations and impact

## Abstract

**Background:**

Journal Impact Factor (JIF) has several intrinsic flaws, which highlight its inability to adequately measure citation distributions or indicate journal quality. Despite these flaws, JIF is still widely used within the academic community, resulting in the propagation of potentially misleading information. A critical review of the usefulness of JIF is needed including an overview of the literature to identify viable alternative metrics. The objectives of this study are: *(1)* to assess the usefulness of JIF by compiling and comparing its advantages and disadvantages; *(2)* to record the differential uses of JIF within research environments; and *(3)* to summarize and compare viable alternative measures to JIF.

**Methods:**

Three separate literature search strategies using MEDLINE and Web of Science were completed to address the three study objectives. Each search was completed in accordance with PRISMA guidelines. Results were compiled in tabular format and analyzed based on reporting frequency.

**Results:**

For objective *(1)*, 84 studies were included in qualitative analysis. It was found that the recorded advantages of JIF were outweighed by disadvantages (18 disadvantages vs. 9 advantages). For objective *(2)*, 653 records were included in a qualitative analysis. JIF was found to be most commonly used in journal ranking (n = 653, 100%) and calculation of scientific research productivity (n = 367, 56.2%). For objective *(3)*, 65 works were included in qualitative analysis. These articles revealed 45 alternatives, which includes 18 alternatives that improve on highly reported disadvantages of JIF.

**Conclusion:**

JIF has many disadvantages and is applied beyond its original intent, leading to inaccurate information. Several metrics have been identified to improve on certain disadvantages of JIF. Integrated Impact Indicator (I3) shows great promise as an alternative to JIF. However, further scientometric analysis is needed to assess its properties.

## Background

In 1955, Eugene Garfield introduced Journal Impact Factor (JIF) as a method of journal rating to be used by librarians when deciding on journal subscriptions [1]. JIF is the total number of citations, received by a journal in a given year, to articles published in the two immediately preceding years, divided by the total number of citable items published by that journal in the past two years [1]. Since its conception, JIF has become one of the most well-recognized and influential bibliometric measures in journal rating [2]. Over time, improvements in the fields of statistics and bibliometrics have led to recognition of flaws intrinsic to JIF as well as misuses of the factor within the scientific community. 

Several researchers have highlighted intrinsic flaws of JIF that limit its accuracy in measuring journal citation distributions [1, 2, 3]. One prominently noted flaw of JIF involves its function as an average measure. Journal citation distributions are commonly skewed and are most appropriately measured by a median measure. However, JIF uses an average calculation, which leaves it prone to the effects of outliers within journal citation distributions [1, 2, 3]. 

Several researchers have also argued against the use of JIF as an indicator of quality. Positive correlations between research quality - defined as innovative, uses appropriate methods and analysis, contains well-thought discussion, and is useful in informing the scientific community [4] - and citations served to motivate researchers to use JIF as an indicator of quality, even though this is an application beyond the metrics original intent [5, 6]. Lack of normalization, such as for citation pool size by genera (general journals have larger citation pools vs. specific journals) and publication citability (reviews receive more citations than case reports), limit JIFs accuracy as an indicator for quality on its own [7, 8]. JIF has been applied as an indicator of journal quality [2, 9] as well as author quality in academic promotions and institutional decisions - such as award of scholarships, research awards, grants, research funds, and evaluation of postgraduate courses [1, 9]. When utilized in these applications, JIF can mislead researchers and influence author decisions [1, 9]. The association of JIF with quality and prestige has also motivated several exploitative practices (e.g., self-citation, increased ratio of non-source to source publications, duplicate publications, selective publishing of highly-citable literature, etc.), further convoluting the accuracy of the impact measured by JIF [2, 9, 10, 11, 12]. 

Due to the aforementioned issues surrounding JIF, it has been suggested that the measure be modified or replaced entirely [2, 3, 9, 12, 13]. These concerns were further advocated in the publication of the Declaration on Research Assessment (DORA) in 2012 [14, 15]. Despite many concerns regarding JIF, its continued use has been justified by its familiarity to researchers and by the inability to provide adequate alternatives for journal rating [1]. However, a more extensive review of all advantages and disadvantages of JIF is needed in order to fully understand viability and consequences of its continued use in the scientific setting. Furthermore, there remains to be a summary of current bibliometric alternatives to explore viable alternatives to JIF.

Using a systematic survey approach, the objectives of this study were to *(1)* assess the usefulness of JIF, *(2)* address its differential uses, and *(3)* identify and provide an overview of alternative metrics. The usefulness of JIF - as a bibliometric measure - was assessed by comparing its advantages and disadvantages. Differential uses of JIF were identified and tabulated. Viable alternative metrics were identified and summarized.

## Assessing JIF Usefulness - Sample 1

### Search strategy

The systematic survey was conducted using Web of Science and Medline (1946-2017), by *(1)* searching the keyword “Journal Impact Factor” and *(2)* limiting results to commentaries, editorials, interviews, lectures, letters, and reviews (Table 1). This search strategy was chosen to capture articles that primarily addressed advantages and/or disadvantages of JIF.

**Table 1.** Search strategy summary.

	Sample 1	Sample 2	Sample 3 (Samples 1 + 2)
**Purpose**	Assessing the usefulness of JIF	Assessing differential uses of JIF	Overview of alternative measures
**Keyword**	Web of Science Medline (1946-2017)	Web of Science Medline (1946-2017)	Web of Science Medline (1946-2017)
**Database(s) searched**	Journal Impact Factor	Journal Impact Factor	Journal Impact Factor
**Inclusion criteria**	Journal articles Commentaries Editorials Interviews Lectures Letters Reviews	Journal articles Reviews Systematic reviews Retrospective cohort studies Cross-sectional studies	Utilizes all articles from Samples 1 and 2
**Exclusion criteria**	Articles excluded if they did not mention advantages or disadvantages of JIF	Articles excluded if they did not utilize JIF in a functional manner	Articles excluded if they did not mention alternative measures or novel bibliometrics

### Eligibility and data extraction

**Figure 1.**
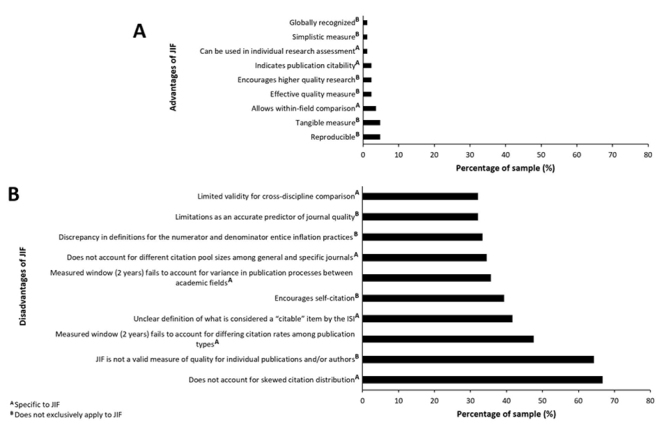
Percentage of sample reporting specific advantages and disadvantages of JIF (n = 84). **(A)** Recorded advantages of JIF. **(B)** Recorded disadvantages of JIF. Subscript letters A and B show which advantages/disadvantages are specific to and do not exclusively apply to JIF, respectively.

Search results were not limited by year. Articles that were not in English were excluded. The inclusion criteria comprised publications stating advantages and/or disadvantages of JIF. Articles that did not mention advantages or disadvantages of JIF were excluded. Study selection and data extraction were performed by a single reviewer (EM) using an inclusion checklist and data extraction form consisting of *a priori* list of advantages/disadvantages (Figure 1), respectively. Issues were solved by consulting other authors who would make the final decision on inclusion.

Data collected consisted of publication characteristics (year and article classification) and the contents of each publication. The contents of each publication were reviewed and tabulated into JIF advantage or disadvantage categories. Information regarding the advantages and disadvantages of JIF was extracted based on categories defined by the authors.

### Study identification and sample characteristics

For sample 1, there were 1701 and 197 (1898 combined total) records retrieved from MEDLINE and Web of Science online databases, respectively (Additional file 1). A total of 84 studies were included for analyses (Additional file 2). Sample 1 was primarily composed of editorials (61.9%, n = 52) with a median publication year of 2013 (Table 2A). 

**Table 2.** Sample characteristics. **(A)** Displays the sample used for the study’s first objective (n = 84). **(B)** Shows the sample used for the study’s second objective (n = 653). **(C)** Presents the sample used for the study’s third objective (n = 65).

A		
Publication type	Number of publications (n, %)	Median publication year
Editorial	52 (61.9%)	2013
Review	11 (13.1%)	2012
Journal article	10 (11.9%)	2009.5
Letter	6 (7.1%)	2014
Commentary	5 (6.0%)	2009
**B**		
**Publication type**	**Number of publications (n, %)**	**Median publication year**
Journal article	572 (87.6%)	2012
Review	41 (6.3%)	2012
Systematic review	33 (5.1%)	2015
Retrospective cohort study	5 (0.8%)	2011
Cross-sectional study	2 (0.3%)	2016
**C**		
**Publication type**	**Number of publications (n, %)**	**Median publication year**
Editorial	22 (33.8%)	2013
Journal article	21 (32.3%)	2012
Review	7 (10.8%)	2012
Letter	4 (6.2%)	2015
Commentary	3 (4.6%)	2009
Perspective	3 (4.6%)	2010
Other	5 (7.7%)	2013

### Advantages and disadvantages of JIF

The most frequently reported advantages of JIF were reproducibility (4.8%, n = 4) and its characteristic as a tangible measure (4.8%, n = 4) (Figure 1A). The most frequently reported disadvantages of JIF included its inability to account for the skewedness of citation distributions (66.7%, n = 56) and not being a valid measure of quality for individual publications and/or authors (64.3%, n = 54) (Figure 1B). Additionally, the list of recorded disadvantages of JIF was much more extensive in comparison to the recorded advantages of JIF (18 vs. 9) (Additional file 3).

## Assessing Differential Uses of JIF - Sample 2

### Search strategy

We systematically searched electronic databases, Web of Science and Medline (1946-2017), by *(1)* using the keyword “Journal Impact Factor” and *(2)* limiting results to journal articles, reviews, systematic reviews, retrospective cohort studies, and cross-sectional studies (Table 1). This search strategy was chosen to capture articles which used JIF functionally, such as in calculations, comparisons, etc.

### Eligibility and data extraction

Search results were not limited by year. Articles that were not in English were excluded. The inclusion criteria comprised publications that utilized JIF functionally. Articles that did not utilize JIF were excluded (ex: studies that mention JIF, but do not utilize it functionally). This criterion allows for the assessment of how JIF is being used in research settings. Study selection and data extraction was performed by two reviewers (EM and MA) using an inclusion checklist and data extraction form consisting of *a priori* list of functional uses of JIF (Table 3), respectively. Disagreement was solved by consulting other authors who would make the final decision on inclusion.

Data collected consisted of publication characteristics (year and article classification) and the contents of each publication. The contents of each publication were reviewed and tabulated into categories pertaining to differential uses of JIF. Information regarding differential uses of JIF was extracted based on categories defined by the authors.

**Table 3.** Percentage of sample displaying specific functional uses of JIF (n = 653).

Functional uses of JIF	Number of publications (n, %)
Journal ranking	653 (100%)
Calculation of scientific research productivity	367 (56.2%)
Debunking associations with quality	22 (3.4%)
Associations with positive results	15 (2.3%)
Functional comparisons to journal evidence index	5 (0.8%)
Functional comparisons to diffusion factor	4 (0.6%)
Correlation with dangerous diseases	4 (0.6%)

### Study identification and sample characteristics

For sample 2, there were 1467 and 519 (1986 combined total) records retrieved from MEDLINE and Web of Science online databases, respectively (Additional file 4). We included a total of 653 studies for analyses (Additional file 5). Sample 2 was primarily composed of journal articles (87.6%, n = 572) with a median publication year of 2012 (Table 2B).

### Functional uses of JIF

Within the analyzed sample, JIF was found to be most commonly used in journal ranking (100%, n = 653) and calculation of scientific research productivity (56.2%, n = 367) (Table 3). 

## Overview of Alternative Measures - Sample 3

### Search strategy

All records from searches 1 and 2 were screened for alternative measures. Titles and abstracts containing the names of known or novel bibliometric alternatives were sorted into a third sample for qualitative analysis (Table 1). This search strategy was chosen to broadly capture a large number of articles addressing alternative bibliometrics.

### Eligibility and data extraction

Search results were not limited by year. Articles that were not in English were excluded. The inclusion criteria comprised publications that mentioned alternative bibliometric measures to JIF. Articles that did not display alternative bibliometric measures were excluded. Study selection and data extraction was performed by three reviewers (EM, ET, and MH) using a selection checklist form developed for the purposes of this study objective. Disagreement was solved by consulting other authors who would make the final decision on inclusion.

Data collected consisted of publication characteristics (year and article classification) and the contents of each publication. The contents of each publication were reviewed and alternative measure properties as well as their advantages/disadvantages compared to JIF were tabulated. 

### Study identification and sample characteristics

After screening a collective 3884 records from the first (n = 1898) and second samples (n = 1986), we included 65 studies for qualitative analysis regarding sample 3 (Additional file 6). Sample 3 was primarily composed of editorials (33.8%, n = 22) (Table 2C).

### Alternative metrics

A total of 45 alternative metrics were identified in sample 3 (n = 65) (Table 4). Key alternative metrics - including Journal to Field Impact Score, SCImago Journal Rank, Source Normalized Impact per Paper (SNIP), Crown indicator, Relative Citation Ratio, Integrated Impact Indicator (I3), h-index, hw-index, hg-index, g-index, D-index, e-index, m-quotient, L-index, R-index, A-index, AR-index, and M-index - metrics were identified by displays of improvement on reported disadvantages of JIF.

**Table 4.** Advantages and disadvantages of alternative methods. The properties of each method were summarized using information compiled from the reviewed commentaries. The advantages and disadvantages of each alternative method were compiled from reviewed commentaries.

Alternative measure	Properties	Advantages vs. JIF	Disadvantages vs. JIF	Key references
SCImago Journal Rank	Calculated by taking the average number of weighted citations received during a selected year divided by the number of documents published in that journal during the previous 3 years.	Relationship coverage between citable items and total output of journal	Favours more prestigious journal outputs	[24, 42, 43]
Age	Age of journal	Not found	Only takes account age of paper in ranking Favours journals established for longer periods of time	[21]
Evaluation of Research Activity	ERA ranking from Crookes et al. (2010)	Accounts for quality of editorial board and peer review process	Bias towards journals with larger global output	[21]
Excellence in Research Australia	Excellence in Research Australia journal ranking from 2010	Useful in comparing journals between subject fields with low JIFs	Has been dropped from assessing research output	[21]
5-Year Journal Impact Factor	Calculated by dividing the number of citations in the JCR year by the total number of articles published in the preceding 5 years	Uses a longer period of measure for greater accuracy	Still carries intrinsic flaws associated with 2-year JIF	[23, 29, 30]
Eigenfactor	Measures the number of times articles from a journal published in the past 5 years have been cited in a Journal Citation Reports year	Eliminates self-citations	Journals have to be assigned to single subject category due to the inability to compare across disciplines similar to JIF	[32, 33, 34]
Article influence score	Calculated using a journals Eigenfactor divided by the fraction of articles published by the journal	Measures the average influence, per article, of the papers published in a journal	Journals have to be assigned to single subject category due to the inability to compare across disciplines similar to JIF	[42, 59, 60]
Source Normalized Impact per Paper (SNIP)	Measures the citation impact by weighting a journal’s total citations to the total number of citations within a subject field per number of publications in the last 3 years	Assesses a journal’s impact within a set context which avoids the disparity encountered between different specialities/fields	Does not take into account the extent to which papers in a field are cited from other fields	[42, 44, 45]
Integrated Impact Indicator (I3)	Citation curves are integrated after proper normalization to the same scales of the hundredth percentile	Can be used across databases Accounts for skewed citation distribution by normalizing citation curves to quartile values	Not found	[47, 48]
CiteScore	Calculated by taking the average number of citations received in a calendar year by all items published in that journal in the preceding three years	It is calculated from the Scopus journal list, which is much larger than the Web of Science list and includes more social sciences and humanities journals Includes citations to all documents in its calculation	Not found	[27, 28]
Free Disposable Hull	Aggregation of four citation-based indicators, JIF, AI, h-index, and Discounted Impact Factor	Provides a ranking along with a journals efficiency level compared to other journals	The four aggregated indicators may not be the most effective combination for optimal accuracy	[40]
Immediacy index	Calculated by dividing a journal’s yearly citations by the number of articles published in a given journal (average number of times an article is cited in its year of publication)	Useful indicator to identify journals publishing in emerging areas of research	Favours journals who publish earlier in the year	[23]
Cited half-life	Number of years, going back from the current year, that account for 50% of the total citations received by a journal in that current year	Gives information on editorial policy	Does not reflect scientific value of journal	[23]
Journal to Field Impact Score	Average number of cited articles in a specific journal and compares this number with that of other journals in the same research field category	Overcomes the limitations of JIF regarding research field productivity	Not found	[23]
Crown indicator	Calculated by dividing the average number of received citations by the average number that could be expected for publications of the same type, during the same year, and published in journals within the same field	Overcomes the limitations of JIF with regards to research field productivity	Size of a research group influences its productivity - quite simply, the more researchers in a group, the larger the number of published articles	[23]
Retraction Rate	Amount of papers retracted in a given period	Measure of quality of journal	Number can be skewed as a large number of articles are retracted for fraud	[36, 61]
Citation Half-Life	The median age of articles cited in a journal	Takes all the ages of all of the articles cited in a journal into consideration	Only accounts for age of paper in ranking	[24]
Citations 2011 JCR/WoS	Total amount of citations obtained by an article in the JCR/WoS database	Eliminates bias towards journals that publish less ‘citable items’ and have resultant inflated JIF Gives more recognition to less cited articles in highly cited journals	Only factors in total number of citations from a journal	[21]
Citations 2011 Scopus	Total amount of citations obtained by an article in the Scopus database	Uses journals not listed within JCR Eliminates bias towards journals that publish less ‘citable items’ and have resultant inflated JIF	Only lists citations from 1996 onwards	[21]
Altmetrics	The score is a weighted count of all of the mentions Altmetric has tracked for an individual research output	Scores quantify the digital attention an article receives in a multitude of online sources Social media, Wikipedia, public policy documents, blogs, and mainstream news are tracked and screened by the Altmetric database	Not a direct substitute for traditional markers of scientific importance	[27, 35, 36]
Relative Citation Ratio	A field-normalized metric that shows the scientific influence of one or more articles relative to the average NIH-funded paper (average NIH paper is composed of the articles co-citation network)	Replacing journal level with relative article-level assessment would place the many highly influential articles that appear in JIF < 28 journals on an equal footing with those in JIF ≥ 28 journals	Can be skewed towards authors with better "reputation"	[46]
Citation Counts	Total amount of citations a journal article accumulates	Can measure impact and influence	Slow to collect data	[25, 26]
Comments	Comments on a paper	Can provide valuable and immediate feedback	Currently sparse and require a change in research reward culture to improve quality	[25]
Bookmarking statistics	Total amount of times an article gets bookmarked into a personal library	Rapid to collect and contain high quality information	Novel and untested metric	[25]
D-index	Defined as the number of papers with download number ≥ d Index for popularity of journal articles	Analyzes authors suitability of their texts to a specific audience Not effected by citation outliers	Values can be inflated (both JIF and D-index can be inflated)	[54]
Scopus trend line	Average amount of times an article is cited in the year it is published	Gives year-to-year comparisons	Citations can change drastically from year to year	[43]
AR-index	Calculated by taking the square root of the sum of a paper’s citations divided by the number of years since its publication Performance and time dependent	Value that can decrease therefore a researcher cannot rest on his laurels Evaluates impact of individual authors	Not designed to be a metric used to evaluate journals on its own	[36, 51, 56]
Hw-index	Variant of H-index that is dependent on researcher performance and is time-dependent	Accounts for periods of inactivity; can decrease with time Evaluates impact of individual authors	Not designed to be a metric used to evaluate journals on its own	[51]
e-index	Calculated by taking the square root of the surplus of citations in the h-core beyond h^2^	Works as a complement to the h-index to differentiate between scientists with identical h-indices but different citations Evaluates impact of individual authors	Not designed to be a metric used to evaluate journals on its own	[52, 62]
Web Impact Factor	Calculated by taking the number of hyperlinks to a site divided by the number of Web pages inside that site	It can encompass a larger range of journals compared to the Institute for Scientific Information	There are no standards for the quality of data on the web compared to the Institute for Scientific Information	[38]
Download Statistics (counts)	Measures popularity of a research item by total unique number of downloads	Data is quick to collect and update daily Good predictor of future impact	Misleading information and may not directly indicate impact	[25, 26]
Social Media	Between July 2008 and November 2011, all tweets containing links to articles in the Journal of Medical Internet Research (JMIR) were mined	Allows for an accurate measure of social impact	Methods cannot be so easily replicated for all journals	[37]
PageRank	PageRank works by counting the number and quality of links to a page to determine a rough estimate of how important the website is The underlying assumption is that more important websites are likely to receive more links from other websites	Measures prestige	Bias towards journal articles in more prestigious journals	[39]
g-index	Given a set of articles ranked in decreasing order of the number of citations that they received, the g-index is the unique largest number such that the top g articles received together at least g^2^ citations	g-index looks at the overall record of the publisher and not just the most highly cited papers Evaluates impact of individual authors	Favour authors who publish greater volumes of articles	[36, 52, 53]
AWMF’s evaluation of medical research performance	Scores quality of researcher based on whether the individual has contributed to progress in his or her discipline Assessed on 3 different levels: 1st Level: Evaluation of publications a) In recognized scientific journals with peer review b) In other media (books, guidelines etc.) c) Citation by guideline recommendations 2nd Level: Active contributions to scientific organizations or boards and editorships 3rd Level: Leadership in organizing scientific conferences	More holistic assessment of quality, which takes more than the number of citations into account	Time consuming to go through each research report and grade it	[22]
Standardized Average Index	Calculated by finding the percentage of each journal’s JIF and h-index out of the total JIF and h-index sums within a defined discipline, respectively, and taking the average of the sum of these two percentage values €	SA index allows one to evaluate the journal from various angles, such as the scientist, institutions, and scientific research	Google scholar was used to calculate h-index and some journals are not located within Google scholar database limiting the range across disciplines	[41]
L-index	Calculated by squaring the reference citation distribution	Applicable to authors Rewards reliability and regularity Sensitive to highly cited papers when comparing authors	Difficult computation	[63]
m-quotient	Calculated by dividing an h-index score by the number of years the academic researcher has been active (measured as the number of years since the first published paper)	Applicable to authors Allows direct comparisons between individual researchers	“m” generally stabilizes later in a researcher’s career	[36, 51, 56]
h-index	Calculated by counting the number of publications for which an author has been cited at least the same amount of times.	Designed to measure individual impact of researchers Prevents few highly cited articles from heavily influencing a researcher’s, group’s, or journal’s profile Considers a much larger timeline that diminishes the effects of variable citation behaviour (ex: short period of researcher, group, or journal producing lowly cited articles)	Ignores the number of citations to each individual article over and above what is needed to achieve a certain h-index Shows bias increases towards earlier established researchers (h), groups, or journals H-index value must be an integer and may lead to inability to compare between researchers, groups, or journals	[41, 49, 50]
R-Index	Calculated by taking the square root of the sum of citations in the Hirsch core	Evaluates impact of individual authors Quality from various angles such as quality of scientist, institutions and scientific research without being punished for having a high h	This index can be very sensitive to just a very few papers receiving extremely high citation counts.	[53]
A-Index	A-index calculates the expected contributions of individual authors for a specific publication A-index can be applied to obtain C- and P-index The sum of a researcher’s total publications weighted to A-index provides the C-index (collaboration index) The sum of JIFs weighted by A-index provides the P-index (productivity index)	Gives fairer assessment of individual researchers by taking into account relative scientific contributions of the researchers	A-index is a method of weighting scientific contribution and thus is applied to JIF, rather than replaces it In cases where A-index is applied to JIF, it carries the same intrinsic flaws of JIF when A-index weighting is applied to JIF in research calculations of C-index and P-index	[51, 53, 55]
M-Index	Variation of the “a” index using the median instead of the arithmetic mean	Derivative of A-Index shares same advantages	Derivative of A-Index shares same disadvantages	[51]
Hg-index	Calculated by taking the square root of the product of the h- and g- indices	Evaluates the impact of individual authors	Not found	[36]
Journal Authority Factor	Average h-index of a journal’s editors	Prestige of a journal is based on the merit of its own faculty and accolades within a journal	Bias towards journals with more experienced and well published authors as editors	[64]
i10-index	Number of publications with at least 10 citations	Not found	Not found	[26]

## Discussion

### JIF usefulness and its differential uses

Upon examination of the reported advantages and disadvantages of JIF, a substantial difference was found in both the diversity and frequency of reporting. There were a significantly greater number of disadvantages recorded in comparison to advantages (18 vs. 9) (Additional file 3). Furthermore, a large proportion of these disadvantages were mentioned more frequently (>>4.8% of articles) than the most frequently reported advantage of JIF (4.8% of articles) (Figure 1A and 1B). Thus, the reported disadvantages of JIF substantially outweigh the reported advantages. However, to fully assess JIF usefulness, qualitative aspects of the advantages and disadvantages were also analyzed and compared.

Qualitative analysis of the top advantages and disadvantages of JIF (Figure 1A and 1B) provides a more diverse representation of the data. Several of the reported advantages and disadvantages are not specific to JIF itself (ie: applicable to other bibliometric measures or usage of JIF). Advantages, such as “reproducibility”, “tangible measure”, “encourages higher quality research”, “indicates publication citability”, “simplistic measure”, and “globally recognized”, are common characteristics of many impact factors, not just JIF (Figure 1A) [14, 15, 16]. As a result, they do not significantly strengthen the case for JIF usage over other metrics. Similarly, many recorded disadvantages are related to criticisms of JIF being used beyond its original intent (ex: indicator of author quality) or manipulation practices (ex: encouraging self-citation or inflationary practices). These criticisms do not directly target issues with JIF itself; however, they highlight limitations of the factor’s usage. 

The remaining advantages and disadvantages are shown to be JIF-specific and were primarily used in this studies critical analysis of JIF usefulness. Advantages, such as “can be used in individual research assessment” and “indicates publication citability”, are refuted by a larger proportion of disadvantages (“does not account for a skewed distribution” and “not a valid indicator for individual authors and/or publications”) arguing against these statements (Figure 1A and 1B). Since JIF is a metric focused on measuring a journals average citation per article, it is applied beyond its original intent when used to assess individual authors or publications [16]. However, the last advantage, “allows within-field comparison”, is unrefuted as a benefit with several authors advocating for the measures ability to provide rough comparisons within a general field [17].

In comparison, there are six disadvantages specific to JIF, all reported in a larger proportion of the sample (Figure 1B). Several disadvantages are focused on downfalls of using a two-year window. The two-year window has been criticized for its inability to capture the differences in citation rates between publication types or the variance in publication times [18]. Another common criticism is focused on the lack of normalization between general and specialized journal citation pools [7, 8]. Due to the vast differences in size of citation pools for general journals (large citation pools) versus very specialized journals (smaller citation pools), caution should be taken when comparing JIF between general and specialized journals without normalization. Similarly, a lack of normalization in “between-field comparison” was noted as a disadvantage. There are significant differences in citation pool sizes between fields, and thus, this highlights a need for normalization [7, 8]. Lastly, the most reported disadvantage of JIF highlights an intrinsic flaw of the factor. As JIF is an average measure, it does not accurately measure skewed distributions [19]. Since citation distributions are skewed, JIF is not a valid measure for this application as means are heavily influenced by outliers [19]. 

It appears that, within this sample of articles, qualitative analysis indicates a greater number of disadvantages - reported at higher frequencies - that are specific to JIF and outnumber refuting advantages. Thus, the usefulness of JIF - in terms of providing unique and accurate data - was determined to be low and is not highly advocated for in this sample. Interestingly, despite recognition of JIFs low usefulness, JIF is still extensively used in scientific practices [15, 20]. Upon surveying the literature, it can be seen that JIF is predominantly used in journal ranking and the calculation of scientific productivity (Table 4). This remains problematic as JIF does not accurately measure citation distributions and both journal ranking and the calculation of scientific productivity rely on accurate analysis of citation data [1, 2, 3]. This assessment of how JIF is functionally used in the scientific community and the reported pros and cons of JIF have outlined the needs of the research community and promising areas for metric improvement, respectively. In combination, this information can serve as an effective guide in the search for alternative metrics.

### Alternatives to JIF

When reviewing viable alternatives, holistic research review by assembled research committees are not indicator-based and serve as gold standards in assessing the quality of research [21, 22]. As a result, Evaluation of Research Activity, Excellence in Research Australia, and AWMF’s evaluation of medical research performance serve as extensive assessments of research impact and quality. However, using these rating systems in equitable volumes to JIF is questionably feasible [21, 22]. As a result, indicators remain a valuable measure of assessment. In this overview, many different indicators were reviewed and many improve on certain reported disadvantages of JIF. 

Factors, such as age, citation half-life, cited half-life, comments, bookmarking statistics, immediacy index, provide valuable information in complement to JIF, but do not appear to be comparable functional measures of impact on their own [21, 23, 24, 25]. Citation counts, citations 2011 JCR/WoS, and citations 2011 Scopus, provide information on impact through raw citation counts [21, 25, 26]. However, these are total citation measures and do not provide article-level information, like JIF [21, 25, 26]. Factors that are more similar to the 2-year JIF, include CiteScore [27, 28] and the 5-year JIF [23, 29, 30]. Cite Score includes all documents in its calculation as opposed to only citable items in the calculation of JIF [27, 28]. This decreases susceptibility of the factor to be skewed by favouring publication of research items that are not included in the JIF denominator [27, 28, 31]. The 5-year JIF measures citations in the JCR divided by articles published in the previous five years [23, 29, 30]. This uses a longer measurement window for greater accuracy in capturing differing publication citation rates compared to the 2-year JIF [23, 29, 30]. Other citation metrics, such as Eigenfactor and Article influence score, are less prone to the inflation practice of self-citation, providing citation counts more reflective of impact [32, 33, 34]. 

Although many of the reviewed factors are citation-based, several use other measures of impact. Altmetrics [27, 35, 36] and related measures, such as download statistics (counts) [25, 26], social media [37], web impact factor [38], and PageRank [39], utilize internet functions as indicators of impact. Altmetrics is a weighted count of all the mentions altmetrics has tracked for an individual research output [27, 35, 36]. This gains the advantage of measuring impact beyond that captured by citations; however, altmetrics has been mentioned to not be a direct substitute for traditional measures of scientific importance [27, 35, 36]. Download statistics (counts) are mentioned to be a good predictor of popularity; however, they can provide misleading information at times [25, 26]. Social media has been shown to accurately measure social impact; however, the methodology is difficult to replicate with high volumes of journals [37]. Web impact factor is calculated by taking the number of hyperlinks to a site divided by the number of web pages inside the site [38]. This encompasses a larger body of journals than the Institute for Scientific Information (ISI), but lacks data quality standards compared to the ISI [38]. Lastly, PageRank measures prestige of websites through measurement of the number of links to a certain journals website [39]. This seems to have a bias towards journal articles in more prestigious journals [39]. There seems to be great promise in these novel measures as they have the added advantages of tracking impact in the form of web-based attention [25, 26, 27,35, 36, 37, 38, 39]. However, this is related to the use of web-based prediction of impact as opposed to traditional citation-based prediction of impact, which is beyond the scope of this paper.

Certain alternatives were found to combine multiple bibliometrics in a type of composite score, such as free disposable hull [40] and standardized average index [41]. Free disposable hull is an aggregation of four citation metrics (JIF, AI, h-index, and discounted impact factor) while standardized average index utilizes two metrics (JIF and h-index). The combination of multiple factors provides better journal ranking by diversifying the number of outputs considered within one metric; however, these combinations may not be the most effective for optimal accuracy [40, 41]. Further analysis and research is needed to optimize metric combinations; however, these factors still utilize JIF and the reported disadvantages - although offset by other metrics - still apply.

The remaining alternative metrics, such as Journal to Field Impact Score, SCImago Journal Rank, SNIP, Crown indicator, Relative Citation Ratio, I3, h-index, hw-index, hg-index, g-index, D-index, e-index, m-quotient, L-index, R-index, A-index, AR-index, and M-index, serve as key alternative metrics that improve on highly reported disadvantages of JIF. Journal to Field Impact Score [23], SCImago Journal Rank [24, 42, 43], and SNIP [42, 44, 45] are normalized relative to their field of publication, which allows for effective cross-field/discipline comparison. Crown Indicator contains normalization using an average citation of matched publication type within its own field [23]. This accounts for differing citation rates of different publication types as well as prepares a relative number for cross-field comparison [23]. Relative Citation Ratio is field-normalized and compares relative to the average NIH-funded paper, allowing cross-field comparison [46]. However, these factors still use average citations in their calculation and do not accurately measure skewed citation distributions. I3 normalizes citation distributions to the 100^th^ percentile before comparison, allowing it to account for the skewedness of citation distributions [47, 48]. I3 appears to correct for the most reported disadvantage of JIF, inability to account for the skewedness of citation distributions [47, 48]. Additionally, I3 and many other factors (h-index [41, 49, 50], hw-index [51], hg-index [36], g-index [36, 52, 53], D-index [54], e-index [52, 62], m-quotient [36, 51, 56], L-index [63], R-index [53], A-index [51, 53, 55], AR-index [36, 51, 56] and M-index [51]), are metrics developed to be applicable to individual researchers and allow for comparison at the author level. Since, I3 shows substantial utility as well as corrects for several highly reported disadvantages of JIF, it shows promise as a valid alternative.

## Study Considerations

The results obtained from this study are consistent with other studies that conducted smaller reviews of the advantages and disadvantages of JIF [57, 58]. However, there are several limitations to this study’s results. The advantages and disadvantages of JIF were gathered primarily from editorials, commentaries, and letters, which are opinion-based and subjective (Table 2A). Thus, a certain degree of bias should be considered. However, the most reported disadvantage of JIF is heavily supported by standard statistical practice of using the median to measure skewed distributions. Additionally, this study did not look at the negative consequences of relying on impact factors (IF) for any type of rating as it was beyond the scope of the study.

Despite certain limitations, this study has several implications. It has been clearly shown that JIF is highly recognized as a measure that is applied beyond its original means as well as fails to accurately measure citation distributions [1, 2, 3, 9, 10, 11, 12]. It is apparent that many journals are recognizing JIF’s limitations as journals are starting to use other metrics, such as article downloads [26]. As a result, a transition away from JIF may be soon.

## Conclusion

It is clear that there are many opinions among the scientific community supporting that the mentioned disadvantages of JIF significantly outweigh the mentioned advantages. Despite recognition of many disadvantages and misuses of JIF, it is still prominently used in journal ranking and calculation of research productivity, leading to inaccuracies in these assessments. Upon review of the literature, it appears that there are several factors that improve on certain disadvantages of JIF and may function as suitable alternatives in certain settings. Journal to Field Impact Score, SCImago Journal Rank, SNIP, Crown Indicator, and Relative Citation Ratio account for differences across fields, giving more accuracy to cross-field comparison. Author-level indicators, including I3, h-index, hw-index, hg-index, g-index, D-index, e-index, m-quotient, L-index, R-index, A-index, AR-index, and M-index, show greater utility in author-level assessment. Furthermore, I3 improves on the most reported disadvantage of JIF. The reviewed data indicates that this factor is a favourable replacement for JIF. This study functions only to highlight current alternatives that improve on reported disadvantages of JIF, but further scientometric analysis is needed to determine the performance of these indicators within their respective categories.

## Supplementary material

The following online material is available for this article:

Additional file 1. Sample 1 references addressing advantages and disadvantages of JIF.

Additional file 2. PRISMA flowchart detailing systematic search protocol for the study’s first objective (Sample 1 - Additional file 1).

Additional file 3. Percentage of sample reporting specific advantages and disadvantages of JIF.

Additional file 4. Sample 2 references addressing functional uses of JIF.

Additional file 5. PRISMA flowchart detailing systematic search protocol for the study’s second objective (Sample 2).

Additional file 6. Sample 3 references addressing alternative measures to JIF.
