# Supplementary material for: Evaluating Journal Impact Factor: a systematic survey of the pros and cons, and overview of alternative measures
Source: J Venom Anim Toxins Incl Trop Dis. 2020 Aug 31;26:e20190082. doi: 10.1590/1678-9199-JVATITD-2019-0082 (PMC7458102; doi:10.1590/1678-9199-JVATITD-2019-0082)
Supplement: Additional file 1. [file 1678-9199-jvatitd-26-e20190082-s1.pdf]

Journal of Venomous Animals and Toxins including Tropical Diseases
https://doi.org/10.1590/1678-9199-JVATITD-2019-0082

Supplementary Material to "Evaluating Journal Impact Factor: a systematic survey of the pros and cons, and overview of alternative measures"

Additional file 1. Sample 1 references addressing advantages and disadvantages of JIF.

1. Jacso P. A deficiency in the algorithm for calculating the impact factor of scholarly journals: The journal impact factor. CORTEX. 2001 Sep;37(4):590–594.
2. Gaba DM. A Remarkable Journal Impact Factor for Simulation in Healthcare. SIMULATION IN HEALTHCARE. 2011 Dec;6(6):313–315.
3. DePellegrin TA, Johnston M. An Arbitrary Line in the Sand: Rising Scientists Confront the Impact Factor. Genetics. 2015;201(3):811–3.
4. Taylor RLJ. An Incomplete Story Told by a Single Number. Poultry science. 2015;94(9):1995–6.
5. Shashikiran ND. Appraising journals–impact factor, citation index, ...?. Journal of the Indian Society of Pedodontics and Preventive Dentistry. 2013;31(3):133–4.
6. Suhrbier A, Poland GA. Are Impact Factors corrupting truth and utility in biomedical research?. Vaccine. 2013;31(51):6041–2.
7. Brink PA. Article visibility: journal impact factor and availability of full text in PubMed Central and open access. Cardiovascular journal of Africa. 2013;24(8):295–6.
8. Handel MJP. Article-level metrics-it's not just about citations. The Journal of experimental biology. 2014;217(Pt 24):4271–2.
9. Casadevall A, Bertuzzi S, Buchmeier MJ, Davis RJ, Drake H, Fang FC, et al. ASM Journals Eliminate Impact Factor Information from Journal Websites. Clinical microbiology reviews. 2016;29(4):i–ii.
10. Rosendaal FR, Reitsma PH. August editorial: impact factors. Journal of thrombosis and haemostasis : JTH. 2013;11(8):1441–2.
11. Joshi MA. Bibliometric indicators for evaluating the quality of scientifc publications. The journal of contemporary dental practice. 2014;15(2):258–62.
12. Agarwal A, Durairajanayagam D, Tatagari S, Esteves SC, Harlev A, Henkel R, et al. Bibliometrics: tracking research impact by selecting the appropriate metrics. Asian journal of andrology. 2016;18(2):296–309.
13. Corey DR, Wise JA, Fox KR, Stoddard BL. Breakthrough articles: putting science first. Nucleic acids research. 2014;42(18):11273–4.
14. Metze K. Bureaucrats, researchers, editors, and the impact factor: a vicious circle that is detrimental to science. Clinics (Sao Paulo, Brazil). 2010;65(10):937–40.
15. Finch A. Can we do better than existing author citation metrics? BIOESSAYS. 2010 Sep;32(9):744–747.
16. Feetham L. Can you measure the impact of your research?. The Veterinary record. 2015;176(21):542–3.
17. Casadevall A, Fang FC. Causes for the persistence of impact factor mania. mBio. 2014;5(2):e00064–14.
18. Sims JL, McGhee CNJ. Citation analysis and journal impact factors in ophthalmology and vision science journals. Clinical & experimental ophthalmology. 2003;31(1):14–22.
19. Zwahlen M, Junker C, Egger M. Commentary II - The journal impact factor in the evaluation of research quality: villain, scapegoat or innocent bystander? SOZIAL-UND PRAVENTIVMEDIZIN. 2004;49(1):19–22.
20. Ramam M. Concluding remarks. Indian journal of dermatology, venereology and leprology. 2017;83(1):1–3.
21. Walter G, Bloch S, Hunt G, Fisher K. Counting on citations: a flawed way to measure quality. The Medical journal of Australia. 2003;178(6):280–1.
22. Bornmann L, Marx W, Gasparyan AY, Kitas GD. Diversity, value and limitations of the journal impact factor

and alternative metrics. RHEUMATOLOGY INTERNATIONAL. 2012 Jul;32(7):1861–1867.
23. Pulverer B. Dora the Brave. EMBO JOURNAL. 2015 Jun;34(12):1601–1602.
24. Jackson D, Haigh C, Watson R. Editorial: Nurses and publications - the impact of the impact factor. Journal of clinical nursing. 2009;18(18):2537–8.
25. Mavrogenis AF, Ruggieri P, Papagelopoulos PJ. Editorial: Self-citation in Publishing. CLINICAL ORTHOPAEDICS AND RELATED RESEARCH. 2010 Oct;468(10):2803–2807.
26. Martin BR. Editors' JIF-boosting stratagems - Which are appropriate and which not? RESEARCH POLICY. 2016 Feb;45(1):1–7.
27. Jacso P. Eigenfactor and article influence scores in the Journal Citation Reports. ONLINE INFORMATION REVIEW. 2010;34(2):339–348.
28. Misteli T. Eliminating the impact of the Impact Factor. The Journal of cell biology. 2013;201(5):651–2.
29. Mingers J, Yang L. Evaluating journal quality: A review of journal citation indicators, and ranking in business and management. EUROPEAN JOURNAL OF OPERATIONAL RESEARCH. 2017 Feb;257(1):323–337.
30. Yla-Herttuala S. From the impact factor to DORA and the scientific content of articles. Molecular therapy : the journal of the American Society of Gene Therapy. 2015;23(4):609.
31. Bornmann L, Marx W, Schier H. Hirsch-Type Index Values for Organic Chemistry Journals: A Comparison of New Metrics with the Journal Impact Factor. EUROPEAN JOURNAL OF ORGANIC CHEMISTRY. 2009 Apr;(10):1471–1476.
32. Sanchis-Gomar F. How does the journal impact factor affect the CV of PhD students? EMBO REPORTS. 2014 Mar;15(3):207.
33. Rawat S. How is impact factor impacting our research?. Biomedical journal. 2014;37(6):415–6.
34. Citrome L. How we rate: is impact factor the most important measure?. International journal of clinical practice. 2013;67(9):819–20.
35. Zupanc GKH. Impact beyond the impact factor. Journal of comparative physiology A, Neuroethology, sensory, neural, and behavioral physiology. 2014;200(2):113–6.
36. Pulverer B. Impact fact-or fiction?. The EMBO journal. 2013;32(12):1651–2.
37. Alberts B. Impact factor distortions. Science (New York, NY). 2013;340(6134):787.
38. Padubidri JR, Shetty BSK. Impact factor impacting our scientific research - Probable solutions. Biomedical journal. 2016;39(3):226.
39. Kamath PS, Bologna G. Impact factor: misused and overhyped?. Hepatology (Baltimore, Md). 2009;49(6):1787–9.
40. Malay DS. Impact factors and other measures of a journal's influence. The Journal of foot and ankle surgery : official publication of the American College of Foot and Ankle Surgeons. 2013;52(3):285–7.
41. Casadevall A, Fang FC. Impacted science: impact is not importance. mBio. 2015;6(5):e01593–15.
42. Burke D, Phillips LH 2nd. Is the "impact factor" a valid measure of the impact of research published in Clinical Neurophysiology and Muscle & Nerve?. Clinical neurophysiology : official journal of the International Federation of Clinical Neurophysiology. 2012;123(9):1687–90.
43. Woodside AG. Journal and author impact metrics: An editorial. JOURNAL OF BUSINESS RESEARCH. 2009 Jan;62(1):1–4.
44. Kanchan T, Krishan K. Journal impact factor - Handle with care. Biomedical journal. 2016;39(3):227.
45. Lee C-H. Journal impact factor and individual article impact. AMERICAN JOURNAL OF EMERGENCY MEDICINE. 2013 Mar;31(3):624.
46. Seixas NS. Journal Impact Factor Rises, Again. ANNALS OF OCCUPATIONAL HYGIENE. 2013 Aug;57(7):823.
47. Price J, Jeffrey J. Journal impact factor: Bibliometrics and the Journal of School Health. JOURNAL OF SCHOOL HEALTH. 2006 Apr;76(4):123–125.
48. Plebani M. Journal impact factor: the debate continues. Clinical chemistry and laboratory medicine. 2013;51(12):2247–8.
49. Nahata MC. Journal impact factor: what it is and is not. The Annals of pharmacotherapy. 2009;43(1):112–3.
50. Hjorland B. Methods for evaluating information sources: An annotated catalogue. JOURNAL OF INFORMATION SCIENCE. 2012 Jun;38(3):258–268.
51. Marks MS, Marsh M, Schroer TA, Stevens TH. Misuse of journal impact factors in scientific assessment. Traffic (Copenhagen, Denmark). 2013;14(6):611–2.

52. Bertuzzi S, Drubin DG. No shortcuts for research assessment. Molecular biology of the cell. 2013;24(10):1505–6.
53. Anonymous. On impact. Nature methods. 2015;12(8):693.
54. Wu X-F, Fu Q, Rousseau R. On indexing in the Web of Science and predicting journal impact factor. Journal of Zhejiang University Science B. 2008;9(7):582–90.
55. Cartwright VA, McGhee CNJ. Ophthalmology and vision science research. Part 1: Understanding and using journal impact factors and citation indices. Journal of cataract and refractive surgery. 2005;31(10):1999–2007.
56. Cartwright VA, Savino PJ. Ophthalmology journals and the ether: considering Journal Impact Factor and citation analysis in context. Clinical & experimental ophthalmology. 2009;37(9):833–5.
57. Magnus D. Overthrowing the Tyranny of the Journal Impact Factor. AMERICAN JOURNAL OF BIOETHICS. 2013 Jul;13(7):1–2.
58. Triaridis S, Kyrgidis A. Peer review and journal impact factor: the two pillars of contemporary medical publishing. Hippokratia. 2010;14(Suppl 1):5–12.
59. Maximin S, Green D. Practice corner: the science and art of measuring the impact of an article. Radiographics : a review publication of the Radiological Society of North America, Inc. 2014;34(1):116–8.
60. Hunt GE, Cleary M, Walter G. Psychiatry and the Hirsch h-index: The relationship between journal impact factors and accrued citations. Harvard review of psychiatry. 2010;18(4):207–19.
61. Kanchan T, Krishan K. Questioning the impact of journal impact factor on research?. Biomedical journal. 2015;38(5):462.
62. Schweinberger SR, Edwards MG, Neyer FJ. Reflections on impact issues. British journal of psychology (London, England : 1953). 2015;106(2):183–5.
63. Casson RJ, Al-Qureshi SH. Scientific information, journal impact factors and editorial policy. Clinical & experimental ophthalmology. 2010;38(7):655–6.
64. Mavrogenis AF, Ruggieri P, Papagelopoulos PJ. Self-citation in publishing. Clinical orthopaedics and related research. 2010;468(10):2803–7.
65. Honekopp J, Kleber J. Sometimes the impact factor outshines the H index. RETROVIROLOGY. 2008 Oct;5.
66. Bagatin E, Gontijo B. The expansion of a measure: what is a scientific journal impact factor and how important is it for academic Brazilian dermatologists. International journal of dermatology. 2011;50(11):1432–4.
67. Stojanovska J. The Figley Fellowship: an entrance to fundamentals of excellent radiology journalism through the lens of editorship and publishing. AJR American journal of roentgenology. 2015;204(4):689–91.
68. Jones JFX. The impact of impact factors and the ethics of publication. Irish journal of medical science. 2013;182(4):541.
69. Foley JA, Della Sala S. The impact of self-citation. Cortex; a journal devoted to the study of the nervous system and behavior. 2010;46(6):802–10.
70. McVeigh ME, Mann SJ. The Journal Impact Factor Denominator Defining Citable (Counted) Items. JAMA-JOURNAL OF THE AMERICAN MEDICAL ASSOCIATION. 2009 Sep;302(10):1107–1109.
71. Diamandis EP. The Journal Impact Factor is under attack - use the CAPCI factor instead. BMC MEDICINE. 2017 Jan;15.
72. Bornmann L, Pudovkin AI. The Journal Impact Factor Should Not Be Discarded. JOURNAL OF KOREAN MEDICAL SCIENCE. 2017 Feb;32(2):180–182.
73. Vakil N. The journal impact factor: judging a book by its cover. The American journal of gastroenterology. 2005;100(11):2436–7.
74. Gasparyan AY, Nurmashev B, Yessirkepov M, Udovik EE, Baryshnikov AA, Kitas GD. The Journal Impact Factor: Moving Toward an Alternative and Combined Scientometric Approach. JOURNAL OF KOREAN MEDICAL SCIENCE. 2017 Feb;32(2):173–179.
75. Satcher MJ, Litton AG, Waterbor JW, Brooks CM. The Journal of Cancer Education: a retrospective review of quality indicators. Journal of cancer education : the official journal of the American Association for Cancer Education. 2009;24(1):16–21.
76. De Sutter A, van Driel M, Maier M, De Maeseneer J. The new impact factor has arrived. Who cares?. The European journal of general practice. 2015;21(3):153–4.
77. Al-Benna S, Clover J. The role of the journal impact factor: choosing the optimal source of peer-reviewed plastic surgery information. Plastic and reconstructive surgery. 2007;119(2):755–6.

78. Ogden TL, Bartley DL. The ups and downs of journal impact factors. ANNALS OF OCCUPATIONAL HYGIENE. 2008 Mar;52(2):73–82.
79. Pendlebury DA. The use and misuse of journal metrics and other citation indicators. Archivum immunologiae et therapiae experimentalis. 2009;57(1):1–11.
80. Reeves S, Kenaszchuk C, Sawatzky-Girling B, Goldman J. Understanding the "impact" of the impact factor. Journal of interprofessional care. 2012;26(1):2–3.
81. Kanter SL. Understanding the Journal's Impact. Academic medicine : journal of the Association of American Medical Colleges. 2009;84(9):1169–70.
82. Kurmis AP. Understanding the limitations of the journal impact factor. The Journal of bone and joint surgery American volume. 2003;85-A(12):2449–54.
83. McKerahan TL, Carmichael SW. What is the impact factor, anyway?. Clinical anatomy (New York, NY). 2012;25(3):283.
84. Wagner PD. What's in a number?. Journal of applied physiology (Bethesda, Md : 1985). 2011;111(4):951–3.
