# Supplementary material for: Evaluating Journal Impact Factor: a systematic survey of the pros and cons, and overview of alternative measures
Source: J Venom Anim Toxins Incl Trop Dis. 2020 Aug 31;26:e20190082. doi: 10.1590/1678-9199-JVATITD-2019-0082 (PMC7458102; doi:10.1590/1678-9199-JVATITD-2019-0082)
Supplement: Additional file 3. [file 1678-9199-jvatitd-26-e20190082-s3.pdf]

Journal of Venomous Animals and Toxins including Tropical Diseases
https://doi.org/10.1590/1678-9199-JVATITD-2019-0082

Supplementary Material to "Evaluating Journal Impact Factor: a systematic survey of the pros and cons, and overview of alternative measures"

Additional file 3. Percentage of sample reporting specific advantages and disadvantages of JIF.

Table 1. Percentage of sample reporting specific advantages of JIF (n = 84).

Advantages	Number of publications (count, %)
Reproducible	4 (4.8%)
Tangible measure	4 (4.8%)
Allows within-field comparison	3 (3.6%)
Effective quality measure	2 (2.4%)
Encourages scientists to produce higher quality research	2 (2.4%)
Indicates publication citability	2, (2.4%)
Can be used in individual research assessment for academic promotions and recruitment	1 (1.2%)
Simplistic measure	1 (1.2%)
Globally recognized	1 (1.2%)

Table 2. Percentage of sample reporting specific disadvantages of JIF (n = 84).

Number	Disadvantages	Number of publications (count, %)
1	Does not account for skewed citation distribution	56 (66.7%)
2	JIF is not a valid measure of quality for individual publications and/or authors	54 (64.3%)
3	Measured window (2 years) fails to account for differing citation rates among publication types	40 (47.6%)
4	Unclear definition of what is considered a "citable" item by the ISI	35 (41.7%)
5	Encourages self-citation	33 (39.3%)
6	Measured window (2 years) fails to account for variance in publication processes between academic fields	30 (35.7%)
7	Does not account for different citation pool sizes among general and specific journals	29 (34.5%)
8	Discrepancy in definitions for the numerator and denominator entice inflation practices	28 (33.3%)
9	Limitations as an accurate predictor of journal quality	27 (32.1%)
10	Limited validity for cross-discipline comparison	27 (32.1%)
11	Database used in the calculation of JIF (Science Citation Index) do not include citations of journals outside of its database	14 (16.7%)
12	Does not capture real impact of the journal	14 (16.7%)
13	Shows bias towards English journals	12 (14.3%)
14	Year-to-year variability of 10-20%	6 (7.1%)
15	Encourages multiple publication (salami-publishing)	4 (4.8%)
16	Measured window (2 years) promotes holding of research in attempts to maximize citations recorded	2 (2.4%)
17	Only statistically significant to 2 decimal places	2 (2.4%)
18	Too simple	1 (1.2%)
