# Supplementary material for: Evaluating Journal Impact Factor: a systematic survey of the pros and cons, and overview of alternative measures
Source: J Venom Anim Toxins Incl Trop Dis. 2020 Aug 31;26:e20190082. doi: 10.1590/1678-9199-JVATITD-2019-0082 (PMC7458102; doi:10.1590/1678-9199-JVATITD-2019-0082)
Supplement: Additional file 4. [file 1678-9199-jvatitd-26-e20190082-s4.pdf]

Journal of Venomous Animals and Toxins including Tropical Diseases
https://doi.org/10.1590/1678-9199-JVATITD-2019-0082

Supplementary Material to "Evaluating Journal Impact Factor: a systematic survey of the pros and cons, and overview of alternative measures"

Additional file 4. Sample 2 references addressing functional uses of JIF.

1. Eysenbach G. 10 years experience with pioneering open access publishing in health informatics: the Journal of Medical Internet Research (JMIR). Studies in health technology and informatics. 2010;160(Pt 2):1329–33.
2. Foo JYA. A 9-year analysis of bibliographical trends for journals in the subject category of general and internal medicine. Accountability in research. 2009;16(3):127–52.
3. Smith DR. A 30-year citation analysis of bibliometric trends at the Archives of Environmental Health, 1975-2004. Archives of environmental & occupational health. 2009;64 Suppl 1(101282564):43–54.
4. Falagas ME, Michalopoulos AS, Bliziotis IA, Soteriades ES. A bibliometric analysis by geographic area of published research in several biomedical fields, 1995-2003. CANADIAN MEDICAL ASSOCIATION JOURNAL. 2006 Nov;175(11):1389–1390.
5. Clausen H, Wormell I. A bibliometric analysis of IOLIM conferences 1977-1999. JOURNAL OF INFORMATION SCIENCE. 2001;27(3):157–169.
6. Garcia-Romero A, Manuel Estrada-Lorenzo J. A bibliometric analysis of plagiarism and self-plagiarism through Deja vu. Scientometrics. 2014 Oct;101(1):381–396.
7. Joyce CW, Kelly JC, Sugrue C. A bibliometric analysis of the 100 most influential papers in burns. Burns : journal of the International Society for Burn Injuries. 2014;40(1):30–7.
8. Vosner HB, Kokol P, Bobek S, Zeleznik D, Zavrsnik J. A bibliometric retrospective of the Journal Computers in Human Behavior (1991-2015). COMPUTERS IN HUMAN BEHAVIOR. 2016 Dec;65:46–58.
9. Hruby GW, McKiernan J, Bakken S, Weng C. A centralized research data repository enhances retrospective outcomes research capacity: a case report. Journal of the American Medical Informatics Association : JAMIA. 2013;20(3):563–7.
10. Fu LD, Aphinyanaphongs Y, Wang L, Aliferis CF. A comparison of evaluation metrics for biomedical journals, articles, and websites in terms of sensitivity to topic. Journal of biomedical informatics. 2011;44(4):587–94.
11. Haddawy P, Hassan S-U, Asghar A, Amin S. A comprehensive examination of the relation of three citation-based journal metrics to expert judgment of journal quality. JOURNAL OF INFORMETRICS. 2016 Feb;10(1):162–173.
12. Moed H, vanLeeuwen T, Reedijk J. A critical analysis of the journal impact factors of Angewandte Chemie and the Journal of the American Chemical Society - Inaccuracies in published impact factors based on overall citations only. Scientometrics. 1996 Sep;37(1):105–116.
13. Torres D, Gugala Z, Lindsey RW. A dedicated research program increases the quantity and quality of orthopaedic resident publications. Clinical orthopaedics and related research. 2015;473(4):1515–21.
14. Lin Y, Li W, Chen K, Liu Y. A document clustering and ranking system for exploring MEDLINE citations. Journal of the American Medical Informatics Association : JAMIA. 2007;14(5):651–61.
15. Guerrero-Bote VP, Moya-Anegon F. A further step forward in measuring journals' scientific prestige: The SJR2 indicator. JOURNAL OF INFORMETRICS. 2012 Oct;6(4):674–688.
16. Harzing A-W, van der Wal R. A Google Scholar h-Index for Journals: An Alternative Metric to Measure Journal Impact in Economics and Business. JOURNAL OF THE AMERICAN SOCIETY FOR INFORMATION SCIENCE AND TECHNOLOGY. 2009 Jan;60(1):41–46.
17. Long X, Huang J-Z, Ho Y-S. A historical review of classic articles in surgery field. AMERICAN JOURNAL OF SURGERY. 2014 Nov;208(5):841–849.
18. Hsu W-C, Tsai C-F, Li J-H. A hybrid indicator for journal ranking An example from the field of Health Care Sciences and Services. ONLINE INFORMATION REVIEW. 2015;39(7):858–869.

19. Feeley TH, Smith RA, Moon S-I, Anker AE. A journal-level analysis of Health Communication. Health communication. 2010;25(6–7):516–21.
20. Shi D, Rousseau R, Yang L, Li J. A Journal's Impact Factor Is Influenced by Changes in Publication Delays of Citing Journals. JOURNAL OF THE ASSOCIATION FOR INFORMATION SCIENCE AND TECHNOLOGY. 2017 Mar;68(3):780–789.
21. Smith DR. A longitudinal analysis of bibliometric and impact factor trends among the core international journals of nursing, 1977-2008. International journal of nursing studies. 2010;47(12):1491–9.
22. Chang J, Phelan M, Cummings BJ. A meta-analysis of efficacy in pre-clinical human stem cell therapies for traumatic brain injury. EXPERIMENTAL NEUROLOGY. 2015 Nov;273:225–233.
23. Al CMW, Stams GJJM, Bek MS, Damen EM, Asscher JJ, van der Laan P. A meta-analysis of intensive family preservation programs: Placement prevention and improvement of family functioning. CHILDREN AND YOUTH SERVICES REVIEW. 2012 Aug;34(8):1472–1479.
24. Sombatsompop N, Markpin T, Premkamolnetr N. A modified method for calculating the Impact Factors of journals in ISI Journal Citation Reports: Polymer Science Category in 1997-2001. Scientometrics. 2004;60(2):217–235.
25. Haustein S, Lariviere V. A multidimensional analysis of Aslib proceedings - using everything but the impact factor. ASLIB JOURNAL OF INFORMATION MANAGEMENT. 2014;66(4):358–380.
26. Bonnevie E. A multifaceted portrait of a library and information science Journal: the case of the Journal of Information Science. JOURNAL OF INFORMATION SCIENCE. 2003;29(1):11–23.
27. Maria Gomez-Sancho J, Jesus Mancebon-Torrubia M. A new approach to measuring scientific production in JCR journals and its application to Spanish public universities. Scientometrics. 2010 Oct;85(1):271–293.
28. Gonzalez-Pereira B, Guerrero-Bote VP, Moya-Anegon F. A new approach to the metric of journals' scientific prestige: The SJR indicator. JOURNAL OF INFORMETRICS. 2010 Jul;4(3):379–391.
29. Lando T, Bertoli-Barsotti L. A new bibliometric index based on the shape of the citation distribution. PloS one. 2014;9(12):e115962.
30. Moed H, Van Leeuwen T, Reedijk J. A new classification system to describe the ageing of scientific journals and their impact factors. JOURNAL OF DOCUMENTATION. 1998 Sep;54(4):387–419.
31. Bollen J, Van de Sompel H, Hagberg A, Chute R. A principal component analysis of 39 scientific impact measures. PloS one. 2009;4(6):e6022.
32. Fox CS, Bonaca MA, Ryan JJ, Massaro JM, Barry K, Loscalzo J. A randomized trial of social media from Circulation. Circulation. 2015;131(1):28–33.
33. Haley MR. A ranking of journals for the aspiring health economist. APPLIED ECONOMICS. 2016;48(18):1710–1718.
34. Tanner-Smith EE, Polanin JR. A retrospective analysis of dissemination biases in the brief alcohol intervention literature. Psychology of addictive behaviors : journal of the Society of Psychologists in Addictive Behaviors. 2015;29(1):49–62.
35. Ekpo EU, Hogg P, McEntee MF. A Review of Individual and Institutional Publication Productivity in Medical Radiation Science. JOURNAL OF MEDICAL IMAGING AND RADIATION SCIENCES. 2016 Mar;47(1):13–20.
36. Li X. A review of the development and application of the Web impact factor. ONLINE INFORMATION REVIEW. 2003;27(6):407–417.
37. Mingers J, Leydesdorff L. A review of theory and practice in scientometrics. EUROPEAN JOURNAL OF OPERATIONAL RESEARCH. 2015 Oct;246(1):1–19.
38. Cappell MS, Davis M. A significant decline in the American domination of research in gastroenterology with increasing globalization from 1980 to 2005: an analysis of American authorship among 8,251 articles. The American journal of gastroenterology. 2008;103(5):1065–74.
39. Duncan FE, Derman B, Woodruff TK. A small field for fertile science: the low visibility of reproductive science in high impact journals. Journal of assisted reproduction and genetics. 2014;31(5):511–20.
40. Foo JYA. A Study on Journal Self-Citations and Intra-Citing within the Subject Category of Multidisciplinary Sciences. SCIENCE AND ENGINEERING ETHICS. 2009 Dec;15(4):491–501.
41. Chen TW, Razak AR, Bedard PL, Siu LL, Hansen AR. A systematic review of immune-related adverse event reporting in clinical trials of immune checkpoint inhibitors(aEuro). ANNALS OF ONCOLOGY. 2015 Sep;26(9):1824–1829.

42. Riley SP, Swanson B, Brismee J-M, Sawyer SF. A systematic review of orthopaedic manual therapy randomized clinical trials quality. The Journal of manual & manipulative therapy. 2016;24(5):241–252.
43. Saragiotto BT, Maher CG, Moseley AM, Yamato TP, Koes BW, Sun X, et al. A systematic review reveals that the credibility of subgroup claims in low back pain trials was low. JOURNAL OF CLINICAL EPIDEMIOLOGY. 2016 Nov;79:3–9.
44. Samaan Z, Mbuagbaw L, Kosa D, Borg Debono V, Dillenburg R, Zhang S, et al. A systematic scoping review of adherence to reporting guidelines in health care literature. Journal of multidisciplinary healthcare. 2013;6(101512691):169–88.
45. Bernstein M, Desy NM, Matache BA, McKinley TO, Harvey EJ. A ten-year analysis of the research funding program of the orthopaedic trauma association. The Journal of bone and joint surgery American volume. 2013;95(19):e1421–6.
46. Bertoli-Barsotti L, Lando T. A theoretical model of the relationship between the h-index and other simple citation indicators. Scientometrics. 2017 Jun;111(3):1415–1448.
47. Prathap G. A three-dimensional bibliometric evaluation of recent research in India. Scientometrics. 2017 Mar;110(3):1085–1097.
48. Prathap G. A three-dimensional bibliometric evaluation of research in polymer solar cells. Scientometrics. 2014 Oct;101(1):889–898.
49. Hoag CC, Elterman DS, Macneily AE. Abstracts presented at the American Urological Association Annual Meeting: determinants of subsequent peer reviewed publication. The Journal of urology. 2006;176(6 Pt 1):2624–2629.
50. Bigna JJR, Noubiap JJN, Asangbeh SL, Um LN, Sime PSD, Temfack E, et al. Abstracts reporting of HIV/AIDS randomized controlled trials in general medicine and infectious diseases journals: completeness to date and improvement in the quality since CONSORT extension for abstracts. BMC medical research methodology. 2016;16(1):138.
51. Bezzaoucha A, Atif ML, Bouamra A, El Kebboub A, Benzerga M, Maghreb Group of Bibliometric Studies, et al. Algerian medical teachers' research output and its determinants during the 2000-2009 decade. Revue d'epidemiologie et de sante publique. 2014;62(1):33–40.
52. Oosthuizen JC, Fenton JE. Alternatives to the impact factor. The surgeon : journal of the Royal Colleges of Surgeons of Edinburgh and Ireland. 2014;12(5):239–43.
53. Leydesdorff L. Alternatives to the journal impact factor: I3 and the top-10% (or top-25%?) of the most-highly cited papers. Scientometrics. 2012 Aug;92(2, SI):355–365.
54. Liu X-L, Gai S-S, Zhang S-L, Wang P. An Analysis of Peer-Reviewed Scores and Impact Factors with Different Citation Time Windows: A Case Study of 28 Ophthalmologic Journals. PloS one. 2015;10(8):e0135583.
55. Haughom BD, Goldstein Z, Hellman MD, Yi PH, Frank RM, Levine BR. An analysis of references used for the Orthopaedic In-Training Examination: what are their levels of evidence and journal impact factors? Clinical orthopaedics and related research. 2014;472(12):4024–32.
56. Munzer BW, Love J, Shipman BL, Byrne B, Cico SJ, Furlong R, et al. An Analysis of the Top-cited Articles in Emergency Medicine Education Literature. The western journal of emergency medicine. 2017;18(1):60–68.
57. Abt HA. An anomalous journal impact factor. ASTRONOMISCHE NACHRICHTEN. 2006;327(7):737–738.
58. Masood M, Thaliath ET, Bower EJ, Newton JT. An appraisal of the quality of published qualitative dental research. Community dentistry and oral epidemiology. 2011;39(3):193–203.
59. Yu G, Yang D-H, He H-X. An automatic recognition method of journal impact factor manipulation. JOURNAL OF INFORMATION SCIENCE. 2011 Jun;37(3):235–245.
60. Robertson IJ, Corrigan MA, Sheikh A, Lehane E, Hill ADK. An evaluation of Irish general surgical research publications from 2000 to 2009. The surgeon : journal of the Royal Colleges of Surgeons of Edinburgh and Ireland. 2010;8(6):314–7.
61. Davis MA. An Examination of CAM Journals in the Journal Citation Reports. ALTERNATIVE THERAPIES IN HEALTH AND MEDICINE. 2011 Oct;17(5):38–42.
62. Rafferty AR, Wong BBM, Chapple DG. An increasing citation black hole in ecology and evolution. ECOLOGY AND EVOLUTION. 2015 Jan;5(1):196–199.
63. Wagner CS, Leydesdorff L. An Integrated Impact Indicator: A new definition of `Impact' with policy

relevance. RESEARCH EVALUATION. 2012 Sep;21(3):183–188.
64. Sonderstrup-Andersen EM, Sonderstrup-Andersen HHK. An investigation into diabetes researcher's perceptions of the Journal Impact Factor - reconsidering evaluating research. Scientometrics. 2008 Aug;76(2):391–406.
65. Mela GS, Cimmino MA. An overview of rheumatological research in the European Union. Annals of the rheumatic diseases. 1998;57(11):643–7.
66. Dougherty MC, Lin S-Y, McKenna HP, Seers K, Keeney S. Analysis of international content of ranked nursing journals in 2005 using ex post facto design. Journal of advanced nursing. 2011;67(6):1358–69.
67. Michaelis LC, Ratain MJ. Analysis of phase II studies for the year 2002: Design, conclusions and impact factors of oncology vs. non-oncology trials. Journal of clinical oncology : official journal of the American Society of Clinical Oncology. 2005;23(16_suppl):6059.
68. Silvestre J, Zhang A, Lin SJ. Analysis of References on the Plastic Surgery In-Service Training Exam. Plastic and reconstructive surgery. 2016;137(6):1951–7.
69. Araujo J, Ghiya ND, Calugar A, Popovic T. Analysis of three factors possibly influencing the outcome of a science review process. Accountability in research. 2014;21(4):241–64.
70. Garcia-Romero A, Santin D, Sicilia G. Another brick in the wall: a new ranking of academic journals in Economics using FDH. Scientometrics. 2016 Apr;107(1):91–101.
71. Falagas ME, Charitidou E, Alexiou VG. Article and journal impact factor in various scientific fields. The American journal of the medical sciences. 2008;335(3):188–91.
72. Frandsen T, Rousseau R. Article impact calculated over arbitrary periods. JOURNAL OF THE AMERICAN SOCIETY FOR INFORMATION SCIENCE AND TECHNOLOGY. 2005 Jan;56(1):58–62.
73. Santangelo GM. Article-level assessment of influence and translation in biomedical research. Molecular biology of the cell. 2017;28(11):1401–1408.
74. Meneghini R, Packer AL, Nassi-Calo L. Articles by latin american authors in prestigious journals have fewer citations. PloS one. 2008;3(11):e3804.
75. Paiva CE, Lima JP da SN, Paiva BSR. Articles with short titles describing the results are cited more often. Clinics (Sao Paulo, Brazil). 2012;67(5):509–13.
76. Sanchez FJ. Assessing the Impact of the Psychology of Men & Masculinity, 2000-2008. PSYCHOLOGY OF MEN & MASCULINITY. 2010 Jul;11(3):161–169.
77. Bloching PA, Heinzl H. Assessing the scientific relevance of a single publication over time. SOUTH AFRICAN JOURNAL OF SCIENCE. 2013 Oct;109(9–10).
78. Coleman A. Assessing the value of a journal beyond the impact factor. JOURNAL OF THE AMERICAN SOCIETY FOR INFORMATION SCIENCE AND TECHNOLOGY. 2007 Jun;58(8):1148–1161.
79. Polychronopoulou A, Pandis N, Eliades T. Assessment of publication bias in dental specialty journals. The journal of evidence-based dental practice. 2010;10(4):207–11.
80. Bales ME, Dine DC, Merrill JA, Johnson SB, Bakken S, Weng C. Associating co-authorship patterns with publications in high-impact journals. Journal of biomedical informatics. 2014;52(100970413, d2m):311–8.
81. Madigan S, Wade M, Tarabulsy G, Jenkins JM, Shouldice M. Association Between Abuse History and Adolescent Pregnancy: A Meta-analysis. Journal of Adolescent Health. 2014 Aug;55(2):151–159.
82. Reed DA, Cook DA, Beckman TJ, Levine RB, Kern DE, Wright SM. Association between funding and quality of published medical education research. JAMA-JOURNAL OF THE AMERICAN MEDICAL ASSOCIATION. 2007 Sep;298(9):1002–1009.
83. Perino AC, Hoang DD, Holmes TH, Santangeli P, Heidenreich PA, Perez MV, et al. Association Between Success Rate and Citation Count of Studies of Radiofrequency Catheter Ablation for Atrial Fibrillation Possible Evidence of Citation Bias. Circulation-Cardiovascular Quality and Outcomes. 2014 Sep;7(5):687–692.
84. Probst P, Grummich K, Ulrich A, Buchler MW, Knebel P, Diener MK. Association of industry sponsorship and positive outcome in randomised controlled trials in general and abdominal surgery: protocol for a systematic review and empirical study. Systematic reviews. 2014;3(101580575):138.
85. Calver MC, O'Brien PA, Lilith M. Australasian Plant Pathology: an analysis of authorship and citations in the 21st century. AUSTRALASIAN PLANT PATHOLOGY. 2012;41(2):179–187.
86. Kulkarni AV, Aziz B, Shams I, Busse JW. Author self-citation in the general medicine literature. PloS one. 2011;6(6):e20885.

87. Hakoum MB, Jouni N, Abou-Jaoude EA, Hasbani DJ, Abou-Jaoude EA, Lopes LC, et al. Authors of clinical trials reported individual and financial conflicts of interest more frequently than institutional and nonfinancial ones: a methodological survey. Journal of clinical epidemiology. 2017;(jce, 8801383).
88. Walker RL, Sykes L, Hemmelgarn BR, Quan H. Authors' opinions on publication in relation to annual performance assessment. BMC medical education. 2010;10(101088679):21.
89. Resnik DB, Tyler AM, Black JR, Kissling G. Authorship policies of scientific journals. Journal of medical ethics. 2016;42(3):199–202.
90. Shanahan DR. Auto-correlation of journal impact factor for consensus research reporting statements: a cohort study. PeerJ. 2016;4(101603425):e1887.
91. Lin J-W, Chang C-H, Lin M-W, Ebell MH, Chiang J-H. Automating the process of critical appraisal and assessing the strength of evidence with information extraction technology. Journal of evaluation in clinical practice. 2011;17(4):832–8.
92. Zitt M. Behind citing-side normalization of citations: some properties of the journal impact factor. Scientometrics. 2011 Oct;89(1):329–344.
93. Albion PR. Benchmarking citation measures among the Australian education professoriate. AUSTRALIAN EDUCATIONAL RESEARCHER. 2012 May;39(2):221–235.
94. Carpenter CR, Sarli CC, Fowler SA, Kulasegaram K, Vallera T, Lapaine P, et al. Best Evidence in Emergency Medicine (BEEM) rater scores correlate with publications' future citations. Academic emergency medicine : official journal of the Society for Academic Emergency Medicine. 2013;20(10):1004–12.
95. Fazel S, Lamsma J. Beyond the impact factor? Evidence-based mental health. 2015;18(2):33–5.
96. Vera-Badillo FE, Shapiro R, Ocana A, Amir E, Tannock IF. Bias in reporting of end points of efficacy and toxicity in randomized, clinical trials for women with breast cancer. Annals of oncology : official journal of the European Society for Medical Oncology. 2013;24(5):1238–44.
97. Vanclay JK. Bias in the journal impact factor. Scientometrics. 2009 Jan;78(1):3–12.
98. Vaughan L, Shaw D. Bibliographic and web citations: What is the difference? JOURNAL OF THE AMERICAN SOCIETY FOR INFORMATION SCIENCE AND TECHNOLOGY. 2003 Dec;54(14):1313–1322.
99. Pagel PS, Hudetz JA. Bibliometric analysis of anaesthesia journal editorial board members: correlation between journal impact factor and the median h-index of its board members. British journal of anaesthesia. 2011;107(3):357–61.
100. van Raan AFJ, Visser MS, Van Leeuwen TN, van Wijk E. Bibliometric analysis of psychotherapy research: performance assessment and position in the journal landscape. Psychotherapy research : journal of the Society for Psychotherapy Research. 2003;13(4):511–28.
101. Bayoumy K, MacDonald R, Dargham SR, Arayssi T. Bibliometric analysis of rheumatology research in the Arab countries. BMC research notes. 2016;9(101462768):393.
102. Lee CS. Bibliometric analysis of the Korean Journal of Parasitology: measured from SCI, PubMed, Scopus, and Synapse databases. The Korean journal of parasitology. 2009;47 Suppl(b3d, 9435800):S155–67.
103. Shuaib W, Khan MS, Shahid H, Valdes EA, Alweis R. Bibliometric Analysis of the Top 100 Cited Cardiovascular Articles. American Journal of Cardiology. 2015 Apr;115(7):972–981.
104. Azer SA, Azer S. Bibliometric analysis of the top-cited gastroenterology and hepatology articles. BMJ open. 2016;6(2):e009889.
105. Smith DR, Hazelton M. Bibliometric awareness in nursing scholarship: can we afford to ignore it any longer? Nursing & health sciences. 2011;13(4):384–7.
106. Manriquez J, Andino-Navarrete R, Cataldo-Cerda K, Harz-Fresno I. Bibliometric characteristics of systematic reviews in dermatology: A cross-sectional study through Web of Science and Scopus. DERMATOLOGICA SINICA. 2015 Sep;33(3):154–156.
107. Klimo PJ, Venable GT, Khan NR, Taylor DR, Shepherd BA, Thompson CJ, et al. Bibliometric evaluation of pediatric neurosurgery in North America. Journal of neurosurgery Pediatrics. 2014;14(6):695–703.
108. Franchignoni F, Munoz Lasa S. Bibliometric indicators and core journals in physical and rehabilitation medicine. Journal of rehabilitation medicine. 2011;43(6):471–6.
109. De Sordi JO, Conejero MA, Meireles M. Bibliometric indicators in the context of regional repositories: proposing the D-index. Scientometrics. 2016 Apr;107(1):235–258.
110. Durieux V, Gevenois PA. Bibliometric indicators: quality measurements of scientific publication. Radiology.

2010;255(2):342–51.
111. Suminski RR, Hendrix D, May LE, Wasserman JA, Guillory VJ. Bibliometric measures and National Institutes of Health funding at colleges of osteopathic medicine, 2006-2010. The Journal of the American Osteopathic Association. 2012;112(11):716–24.
112. Mansour AM, El Mollayess G, Habib R, Arabi A, Medawar WA. Bibliometric trends in ophthalmology 1997-2009. INDIAN JOURNAL OF OPHTHALMOLOGY. 2015 Jan;63(1):54–58.
113. Karanatsiou D, Misirlis N, Vlachopoulou M. Bibliometrics and altmetrics literature review Performance indicators and comparison analysis. PERFORMANCE MEASUREMENT AND METRICS. 2017;18(1, SI):16–27.
114. Cooper ID. Bibliometrics basics. Journal of the Medical Library Association : JMLA. 2015;103(4):217–8.
115. Marx W, Bornmann L. Bibliometrics in research evaluation background, significance, and limitations. SOZIALE WELT-ZEITSCHRIFT FUR SOZIALWISSENSCHAFTLICHE FORSCHUNG UND PRAXIS. 2015;66(2):161+.
116. Sole CV, Calvo FA, Ferrer C, Pascau J, Marsiglia H. Bibliometrics of intraoperative radiotherapy Analysis of technology, practice and publication tendencies. STRAHLENTHERAPIE UND ONKOLOGIE. 2014 Nov;190(12):1111–1116.
117. Royle P, Waugh N. Bibliometrics of NIHR HTA monographs and their related journal articles. BMJ open. 2015;5(2):e006595.
118. Royle P, Kandala N-B, Barnard K, Waugh N. Bibliometrics of systematic reviews: analysis of citation rates and journal impact factors. Systematic reviews. 2013;2(101580575):74.
119. Smith DR, Hazelton M. Bibliometrics, citation indexing, and the journals of nursing. Nursing & health sciences. 2008;10(4):260–5.
120. Winkmann G, Schweim H. Biomedical databases and the Journal Impact Factor. Deutsche Medizinische Wochenschrift. 2000 Sep;125(38):1133–1141.
121. Maojo V, Garcia-Remesal M, Bielza C, Crespo J, Perez-Rey D, Kulikowski C. Biomedical Informatics Publications: a Global Perspective Part II: Journals. METHODS OF INFORMATION IN MEDICINE. 2012;51(2):131–137.
122. Selamnia M, Tali-Maamar H. Biomedical research in developing countries. I–The Algerian case (1993-1998). La Tunisie medicale. 2003;81(7):456–60.
123. Vaught J, Nale S. Biopreservation and Biobanking Mid-Year Report. Biopreservation and biobanking. 2016;14(4):263.
124. Abbas AM. Bounds and inequalities relating h-index, g-index, e-index and generalized impact factor: an improvement over existing models. PloS one. 2012;7(4):e33699.
125. Mohallem JR, da Fonseca NE. Brazilian impact factor of physics journals–the third side of the coin. Anais da Academia Brasileira de Ciencias. 2015;87(2):1233–8.
126. Sanchez FJ. Building the Scientific Basis of the Psychological Study of Men and Masculinity. PSYCHOLOGY OF MEN & MASCULINITY. 2015 Jan;16(1):1–10.
127. Maini P, Schnell S, Jolliffe S. Bulletin of mathematical biology - Facts, figures and comparisons. BULLETIN OF MATHEMATICAL BIOLOGY. 2004 Jul;66(4):595–603.
128. Eysenbach G. Can tweets predict citations? Metrics of social impact based on Twitter and correlation with traditional metrics of scientific impact. Journal of medical Internet research. 2011;13(4):e123.
129. Beaufrere H, Kearney MT, Tully TN Jr. Can we trust the avian medical literature: survey and critical appraisal of the use of statistics in avian medicine from 2007 to 2011. Journal of Exotic Pet Medicine. 2015 Oct;24(4):415–426.
130. Potempa KM, Redman RW, Anderson CA. Capacity for the advancement of nursing science: issues and challenges. Journal of professional nursing : official journal of the American Association of Colleges of Nursing. 2008;24(6):329–36.
131. Firat AC, Araz C, Kayhan Z. Case reports: Should we do away with them? Journal of Clinical Anesthesia. 2017 Feb;37:74–76.
132. Ramos A, Mendoza LC, Rabasa F, Bolibar I, Puig T, Corcoy R. Case-control studies in diabetes. Do they really use a case-control design? Acta diabetologica. 2017;54(7):631–634.
133. Read RJ, Kleywegt GJ. Case-controlled structure validation. Acta crystallographica Section D, Biological crystallography. 2009;65(Pt 2):140–7.

134. Salvi S, Gurioli G, De Giorgi U, Conteduca V, Tedaldi G, Calistri D, et al. Cell-free DNA as a diagnostic marker for cancer: current insights. ONCOTARGETS AND THERAPY. 2016;9:6549–6559.
135. Loughborough W, Dale H, Wareham JH, Youssef AH, Rodrigues MA, Rodrigues JCL. Characteristics and trends in publication of scientific papers presented at the European Congress of Radiology: a comparison between 2000 and 2010. Insights into Imaging. 2016 Oct;7(5):755–762.
136. Baek S, Yoon DY, Min KJ, Lim KJ, Seo YL, Yun EJ. Characteristics and trends of research on positron emission tomography: a bibliometric analysis, 2002-2012. Annals of Nuclear Medicine. 2014 Jun;28(5):455–462.
137. Kulkarni AV, Busse JW, Shams I. Characteristics associated with citation rate of the medical literature. PloS one. 2007;2(5):e403.
138. van Leeuwen T, Moed H. Characteristics of Journal Impact Factors: The effects of uncitedness and citation distribution on the understanding of journal impact factors. Scientometrics. 2005 Apr;63(2):357–371.
139. Nietert PJ, Wahlquist AE, Herbert TL. Characteristics of recent biostatistical methods adopted by researchers publishing in general/internal medicine journals. Statistics in Medicine. 2013 Jan;32(1):1–10.
140. Qi X, Deng H, Guo X. Characteristics of retractions related to faked peer reviews: an overview. Postgraduate medical journal. 2016;(pfx, 0234135).
141. Pecaric D, Bakovic B, Tudman M. Characteristics of scientific production in Croatia from 1997 to 2014. Qualitative & Quantitative Methods in Libraries. 2015 Jun;259–271.
142. Ozcakar L, Franchignoni F, Kara M, Munoz Lasa S. Choosing a scholarly journal during manuscript submission: the way how it rings true for physiatrists. European journal of physical and rehabilitation medicine. 2012;48(4):643–7.
143. Smith DR. Citation Analysis and Bibliometric Research in the Field of Ergonomics. Human Factors and Ergonomics in Manufacturing & Service Industries. 2010 Jun;20(3):202–210.
144. Smith DR. Citation analysis and impact factor trends of 5 core journals in occupational medicine, 1975-1984. Archives of environmental & occupational health. 2010;65(3):176–9.
145. Smith DR. Citation analysis and impact factor trends of 5 core journals in occupational medicine, 1985-2006. Archives of environmental & occupational health. 2008;63(3):114–22.
146. Sims JL, McGhee CNJ. Citation analysis and journal impact factors in ophthalmology and vision science journals. Clinical & experimental ophthalmology. 2003;31(1):14–22.
147. Haddow G, Genoni P. Citation analysis and peer ranking of Australian social science journals. Scientometrics. 2010 Nov;85(2):471–487.
148. Perneger TV. Citation analysis of identical consensus statements revealed journal-related bias. Journal of clinical epidemiology. 2010;63(6):660–4.
149. Hunt GE, Happell B, Chan SW-C, Cleary M. Citation analysis of mental health nursing journals: How should we rank thee? International Journal of Mental Health Nursing. 2012 Dec;21(6):576–580.
150. Coelho DH, Edelmayer LW, Fenton JE. Citation analysis of otorhinolaryngology journals: follow-up study. The Journal of laryngology and otology. 2015;129(5):489–93.
151. Fan JC, McGhee CNJ. Citation analysis of the most influential authors and ophthalmology journals in the field of cataract and corneal refractive surgery 2000-2004. Clinical and Experimental Ophthalmology. 2008 Feb;36(1):54–61.
152. Sangwal K. Citation and impact factor distributions of scientific journals published in individual countries. JOURNAL OF INFORMETRICS. 2013;7(2):487–504.
153. Chapman S, Ragg M, McGeechan K. Citation bias in reported smoking prevalence in people with schizophrenia. Australian and New Zealand Journal of Psychiatry. 2009;43(3):277–282.
154. Adusumilli P, Chan M, Ben-Porat L, Mullerad M, Stiles B, Tuorto S, et al. Citation characteristics of basic science research publications in general surgical journals. Journal of Surgical Research. 2005 Oct;128(2, SI):168–173.
155. Kim ES, Yoon DY, Kim HJ, Jeon HJ, Lee JY, Cho B-M, et al. Citation classics in neurointerventional research: a bibliometric analysis of the 100 most cited articles. Journal of neurointerventional surgery. 2017;9(5):508–511.
156. Wong ELY, Tam WWS, Wong FCY, Cheung AWL. Citation classics in nursing journals: the top 50 most frequently cited articles from 1956 to 2011. Nursing research. 2013;62(5):344–51.
157. Stack S. Citation classics in suicide and life threatening behavior: a research note. Suicide & life-threatening

behavior. 2012;42(6):628–39.
158. Uthman OA, Okwundu CI, Wiysonge CS, Young T, Clarke A. Citation classics in systematic reviews and meta-analyses: who wrote the top 100 most cited articles? PloS one. 2013;8(10):e78517.
159. Lustosa LA, Chalco MEP, Borba C de M, Higa AE, Almeida RMVR. Citation distribution profile in Brazilian journals of general medicine. São Paulo Medical Journal/Revista Paulista De Medicina. 2012;130(5):314–7.
160. Nigam A, Nigam PK. Citation Index and Impact factor. Indian journal of dermatology, venereology and leprology. 2012;78(4):511–6.
161. Kapeller J. Citation Metrics: Serious Drawbacks, Perverse Incentives, and Strategic Options for Heterodox Economics. American Journal of Economics and Sociology. 2010 Nov;69(5):1376–1408.
162. Slyder JB, Stein BR, Sams BS, Walker DM, Beale BJ, Feldhaus JJ, et al. Citation pattern and lifespan: a comparison of discipline, institution, and individual. Scientometrics. 2011 Dec;89(3):955–966.
163. Ohmer ME, Bishop PJ. Citation rate and perceived subject bias in the amphibian-decline literature. Conservation biology : the journal of the Society for Conservation Biology. 2011;25(1):195–9.
164. Lopez J, Calotta N, Doshi A, Soni A, Milton J, May JW Jr, et al. Citation Rate Predictors in the Pi Surery Literture. Journal of Surgical Education. 2017 Apr;74(2):191–198.
165. Milojevic S, Radicchi F, Bar-Ilan J. Citation success index - An intuitive pair-wise journal comparison metric. Journal of Informetrics. 2017 Feb;11(1):223–231.
166. Moed HF, Colledge L, Reedijk J, Moya-Anegon F, Guerrero-Bote V, Plume A, et al. Citation-based metrics are appropriate tools in journal assessment provided that they are accurate and used in an informed way. Scientometrics. 2012 Aug;92(2, SI):367–376.
167. Zhang Z, Poucke SV. Citations for Randomized Controlled Trials in Sepsis Literature: The Halo Effect Caused by Journal Impact Factor. PloS one. 2017;12(1):e0169398.
168. Etter J-F, Stapleton J. Citations to trials of nicotine replacement therapy were biased toward positive results and high-impact-factor journals. Journal of clinical epidemiology. 2009;62(8):831–7.
169. Abramo G, D'Angelo CA, Di Costa F. Citations versus journal impact factor as proxy of quality: could the latter ever be preferable? Scientometrics. 2010 Sep;84(3):821–833.
170. da Silva JAT, Memon AR. CiteScore: A cite for sore eyes, or a valuable, transparent metric? Scientometrics. 2017 Apr;111(1):553–556.
171. Zitt M. Citing-side normalization of journal impact: A robust variant of the Audience Factor. JOURNAL OF INFORMETRICS. 2010 Jul;4(3):392–406.
172. Mandaleson A, Lee Y, Kerr C, Graham HK. Classifying cerebral palsy: are we nearly there?. Journal of pediatric orthopedics. 2015;35(2):162–6.
173. Lira RPC, Leal FS, Goncalves FA, Amorim FHR, Felix JPF, Arieta CEL. Clinical trials in Brazilian journals of ophthalmology: where we are. Arquivos brasileiros de oftalmologia. 2013;76(1):21–5.
174. Crespi CM, Maxwell AE, Wu S. Cluster randomized trials of cancer screening interventions: are appropriate statistical methods being used?. Contemporary clinical trials. 2011;32(4):477–84.
175. Jackson CL, Greenhalgh T. Co-creation: a new approach to optimising research impact?. The Medical journal of Australia. 2015;203(7):283–4.
176. Yin C-Y, Aris MJ, Chen X. Combination of Eigenfactor (TM) and h-index to evaluate scientific journals. Scientometrics. 2010 Sep;84(3):639–648.
177. Coronado F, Chen GM, Smith CK, Glynn MK. Communicating Science: The Role of Centers for Disease Control and Prevention's Field-Based Epidemic Intelligence Service Officers, 2009-2014. JOURNAL OF PUBLIC HEALTH MANAGEMENT AND PRACTICE. 2016 Aug;22(4):403–408.
178. Bador P, Lafouge T. Comparative analysis between impact factor and h-index for pharmacology and psychiatry journals. Scientometrics. 2010 Jul;84(1):65–79.
179. Bador P, Lafouge T. Comparative Analysis between Impact Factor and h-Index for Psychiatry Journals. CANADIAN JOURNAL OF INFORMATION AND LIBRARY SCIENCE-REVUE CANADIENNE DES SCIENCES DE L INFORMATION ET DE BIBLIOTHECONOMIE. 2011 Jun;35(2):109–121.
180. Bador P, Lafouge T. Comparative Analysis of Impact Factor and h-index for Pharmacology Journals. THERAPIE. 2010 Apr;65(2):129–137.
181. Dorta-Gonzalez P, Dorta-Gonzalez MI. Comparing journals from different fields of science and social science through a JCR subject categories normalized impact factor. Scientometrics. 2013 May;95(2):645–672.
182. Schloegl C, Gorraiz J. Comparison of citation and usage indicators: the case of oncology journals.

Scientometrics. 2010 Mar;82(3):567–580.
183. Yang Z-G, Gao F, Zhang C-T. Comparison of journal self-citation rates between some Chinese and non-Chinese international journals. PloS one. 2012;7(11):e49001.
184. Schoepfel J, Prost H. Comparison of SCImago Journal Rank Indicator (SJR) with JCR journal impact factor (IF) for French journals. PSYCHOLOGIE FRANCAISE. 2009 Dec;54(4):287–305.
185. Falagas ME, Kouranos VD, Arencibia-Jorge R, Karageorgopoulos DE. Comparison of SCImago journal rank indicator with journal impact factor. FASEB journal : official publication of the Federation of American Societies for Experimental Biology. 2008;22(8):2623–8.
186. Falagas ME, Kouranos VD, Michalopoulos A, Rodopoulou SP, Batsiou MA, Karageorgopoulos DE. Comparison of the distribution of citations received by articles published in high, moderate, and low impact factor journals in clinical medicine. Internal medicine journal. 2010;40(8):587–91.
187. Kulasegarah J, Fenton JE. Comparison of the h index with standard bibliometric indicators to rank influential otolaryngologists in Europe and North America. European archives of oto-rhino-laryngology : official journal of the European Federation of Oto-Rhino-Laryngological Societies (EUFOS) : affiliated with the German Society for Oto-Rhino-Laryngology - Head and Neck Surgery. 2010;267(3):455–8.
188. Cox R, McIntyre KM, Sanchez J, Setzkorn C, Baylis M, Revie CW. Comparison of the h-Index Scores Among Pathogens Identified as Emerging Hazards in North America. Transboundary and emerging diseases. 2016;63(1):79–91.
189. Lee S-Y, Teoh PJ, Camm CF, Agha RA. Compliance of randomized controlled trials in trauma surgery with the CONSORT statement. The journal of trauma and acute care surgery. 2013;75(4):562–72.
190. Wolthoff A, Lee Y, Ghohestani RF. Comprehensive citation factor: a novel method in ranking medical journals. European journal of dermatology : EJD. 2011;21(4):495–500.
191. Moed HF. Comprehensive indicator comparisons intelligible to non-experts: the case of two SNIP versions. Scientometrics. 2016 Jan;106(1):51–65.
192. Resnik DB, Konecny B, Kissling GE. Conflict of Interest and Funding Disclosure Policies of Environmental, Occupational, and Public Health Journals. Journal of occupational and environmental medicine. 2017;59(1):28–33.
193. Cooper RJ, Gupta M, Wilkes MS, Hoffman JR. Conflict of Interest Disclosure Policies and Practices in Peer-reviewed Biomedical Journals. Journal of general internal medicine. 2006;21(12):1248–52.
194. Norris SL, Holmer HK, Ogden LA, Selph SS, Fu R. Conflict of interest disclosures for clinical practice guidelines in the national guideline clearinghouse. PloS one. 2012;7(11):e47343.
195. Kesselheim AS, Wang B, Studdert DM, Avorn J. Conflict of interest reporting by authors involved in promotion of off-label drug use: an analysis of journal disclosures. PLoS medicine. 2012;9(8):e1001280.
196. Lundh A, Barbateskovic M, Hrobjartsson A, Gotzsche PC. Conflicts of interest at medical journals: the influence of industry-supported randomised trials on journal impact factors and revenue - cohort study. PLoS medicine. 2010;7(10):e1000354.
197. Rocha e Silva M. Continuously variable rating: a new, simple and logical procedure to evaluate original scientific publications. Clinics (Sao Paulo, Brazil). 2011;66(12):2099–104.
198. Fosbol EL, Fosbol PL, Harrington RA, Eapen ZJ, Peterson ED. Conversion of cardiovascular conference abstracts to publications. Circulation. 2012;126(24):2819–25.
199. Yacyshyn EA, Soong LC. Conversion rates of abstracts presented at the Canadian Rheumatology Association Annual Meetings into full-text journal articles. Rheumatology international. 2017;37(6):949–953.
200. Costa LOP, Moseley AM, Sherrington C, Maher CG, Herbert RD, Elkins MR. Core journals that publish clinical trials of physical therapy interventions. Physical therapy. 2010;90(11):1631–40.
201. Finardi U. Correlation between Journal Impact Factor and Citation Performance: An experimental study. JOURNAL OF INFORMETRICS. 2013;7(2):357–370.
202. Mirsaeid SJG, Motamedi N, Ghorbani NR. Correlation between Self-Citation and Impact Factor in Iranian English Medical Journals in WoS and ISC: A Comparative Approach. IRANIAN JOURNAL OF PUBLIC HEALTH. 2015 Sep;44(9):1234–1243.
203. Elkins MR, Maher CG, Herbert RD, Moseley AM, Sherrington C. Correlation between the Journal Impact Factor and three other journal citation indices. Scientometrics. 2010 Oct;85(1):81–93.
204. Walter G, Bloch S, Hunt G, Fisher K. Counting on citations: a flawed way to measure quality. The Medical journal of Australia. 2003;178(6):280–1.

205. Rendon H, Johnson MA. Coverage of Mexico in United States media: Phases of academic scholarship. INTERNATIONAL COMMUNICATION GAZETTE. 2015 Dec;77(8):735–752.
206. Lindebaum D. Critical Essay: Building new management theories on sound data? The case of neuroscience. Human relations; studies towards the integration of the social sciences. 2016;69(3):537–550.
207. Spiroski M. Current biomedical scientific impact (2013) of institutions, academic journals and researchers in the Republic of Macedonia. Prilozi (Makedonska akademija na naukite i umetnostite Oddelenie za medicinski nauki). 2014;35(3):67–80.
208. Kurmis A. Current concepts review - Understanding the limitations of the journal impact factor. JOURNAL OF BONE AND JOINT SURGERY-AMERICAN VOLUME. 2003 Dec;85A(12):2449–2454.
209. Zaidi I, Singh S, Sinha A, Dwivedi R. Current views and implications of journal impact factor: A key note. Indian journal of dentistry. 2015;6(2):113–4.
210. Zaidi I, Singh S, Sinha A, Dwivedi R. Current views and implications of journal impact factor: A key note. South Asian journal of cancer. 2016;5(4):191.
211. Piwowar HA, Vision TJ. Data reuse and the open data citation advantage. PEERJ. 2013 Oct;1.
212. Narin F. Decades of progress, or the progress of decades? Scientometrics. 2012 Aug;92(2, SI):391–393.
213. Habibzadeh P. Decay of references to Web sites in articles published in general medical journals: mainstream vs small journals. Applied clinical informatics. 2013;4(4):455–64.
214. Sardanelli F, Sconfienza LM. Declining impact factor of radiologic journals: a matter for debate. AJR American journal of roentgenology. 2013;201(3):W391–3.
215. Nishijima DK, Yadav K, May L, Kraynov L, Courtney DM, 2011-2012 and 2012-2013 Society for Academic Emergency Medicine Research Committees. Description and productivity of emergency medicine researchers receiving K23 or K08 mentored research career development awards. Academic emergency medicine : official journal of the Society for Academic Emergency Medicine. 2013;20(6):611–7.
216. Stallings J, Vance E, Yang J, Vannier MW, Liang J, Pang L, et al. Determining scientific impact using a collaboration index. Proceedings of the National Academy of Sciences of the United States of America. 2013;110(24):9680–5.
217. Jacso P. Differences in the rank position of journals by Eigenfactor metrics and the five-year impact factor in the Journal Citation Reports and the Eigenfactor Project web site. ONLINE INFORMATION REVIEW. 2010;34(3):496–508.
218. von Elm E, Poglia G, Walder B, Tramer MR. Different patterns of duplicate publication: an analysis of articles used in systematic reviews. JAMA. 2004;291(8):974–80.
219. Bridoux V, Moutel G, Schwarz L, Michot F, Herve C, Tuech J-J. Disclosure of funding sources and conflicts of interest in phase III surgical trials: survey of ten general surgery journals. World journal of surgery. 2014;38(10):2487–93.
220. Alasbali T, Smith M, Geffen N, Trope GE, Flanagan JG, Jin Y, et al. Discrepancy between results and abstract conclusions in industry- vs nonindustry-funded studies comparing topical prostaglandins. American journal of ophthalmology. 2009;147(1):33–38.e2.
221. van Leeuwen T. Discussing some basic critique on Journal Impact Factors: revision of earlier comments. Scientometrics. 2012;92(2):443–455.
222. Bornmann L, Marx W, Gasparyan AY, Kitas GD. Diversity, value and limitations of the journal impact factor and alternative metrics. RHEUMATOLOGY INTERNATIONAL. 2012 Jul;32(7):1861–1867.
223. Teunis T, Nota SPFT, Schwab JH. Do corresponding authors take responsibility for their work? A covert survey. Clinical orthopaedics and related research. 2015;473(2):729–35.
224. Dorta Gonzalez MI, Dorta Gonzalez P. Do fixed citation windows match impact maturation rates of scientific journals? INVESTIGACION BIBLIOTECOLOGICA. 2016 Apr;30(68):73–89.
225. Creavin ST, Creavin AL, Mallen CD. Do GPs respond to postal questionnaire surveys? A comprehensive review of primary care literature. FAMILY PRACTICE. 2011 Aug;28(4):461–467.
226. Yin C-Y. Do impact factor, h-index and Eigenfactor (TM) of chemical engineering journals correlate well with each other and indicate the journals' influence and prestige? CURRENT SCIENCE. 2011 Mar;100(5):648–653.
227. Ralston R, Gall C, Brahmi FA. Do local citation patterns support use of the impact factor for collection development?. Journal of the Medical Library Association : JMLA. 2008;96(4):374–8.
228. Prathap G, Mini S, Nishy P. Does high impact factor successfully predict future citations? An analysis using

Peirce’s measure. Scientometrics. 2016 Sep;108(3):1043–1047.
229. Lansingh VC, Carter MJ. Does open access in ophthalmology affect how articles are subsequently cited in research?. Ophthalmology. 2009;116(8):1425–31.
230. Davis PM. Does open access lead to increased readership and citations? A randomized controlled trial of articles published in APS journals. The Physiologist. 2010;53(6):197–1.
231. Carroll AE, Sox CM, Tarini BA, Ringold S, Christakis DA. Does presentation format at the Pediatric Academic Societies’ annual meeting predict subsequent publication?. Pediatrics. 2003;112(6 Pt 1):1238–41.
232. Bornmann L, Leydesdorff L. Does quality and content matter for citedness? A comparison with para-textual factors and over time. JOURNAL OF INFORMETRICS. 2015 Jul;9(3):419–429.
233. Yu L, Yu H. Does the average JIF percentile make a difference? Scientometrics. 2016 Dec;109(3):1979–1987.
234. Fleurence RL, Spackman DE, Hollenbeak C. Does the funding source influence the results in economic evaluations? A case study in bisphosphonates for the treatment of osteoporosis. PharmacoEconomics. 2010;28(4):295–306.
235. Czarnecki L, Kazmierkowski MP, Rogalski A. Doing Hirsch proud; shaping H-index in engineering sciences. BULLETIN OF THE POLISH ACADEMY OF SCIENCES-TECHNICAL SCIENCES. 2013 Mar;61(1):5–21.
236. Hong CJ, McInnes MDF, Hibbert RM, Dang W, Mir ZM, Li D, et al. Duplicate Publication in Radiology Journals. AMERICAN JOURNAL OF ROENTGENOLOGY. 2015 May;204(5):W573–W578.
237. Groesser SN. Dynamics of Journal Impact Factors. SYSTEMS RESEARCH AND BEHAVIORAL SCIENCE. 2012 Dec;29(6, SI):624–644.
238. Haslam N, Laham S. Early-career scientific achievement and patterns of authorship: the mixed blessings of publication leadership and collaboration. RESEARCH EVALUATION. 2009 Dec;18(5):405–410.
239. Foo JYA. EFFECT OF BIBLIOGRAPHICAL CLASSIFICATION ON THE IMPACT FACTOR OF SCIENCE- AND ENGINEERING-BASED JOURNALS. ACCOUNTABILITY IN RESEARCH-POLICIES AND QUALITY ASSURANCE. 2009;16(1):1–12.
240. Miguel Campanario J, Carretero J, Marangon V, Molina A, Ros G. Effect on the journal impact factor of the number and document type of citing records: a wide-scale study. Scientometrics. 2011 Apr;87(1):75–84.
241. Petridis K, Malesios C, Arabatzis G, Thanassoulis E. Efficiency analysis of forestry journals: Suggestions for improving journals’ quality. JOURNAL OF INFORMETRICS. 2013;7(2):505–521.
242. Jacso P. Eigenfactor and article influence scores in the Journal Citation Reports. ONLINE INFORMATION REVIEW. 2010;34(2):339–348.
243. Reynolds JC, Menegazzi JJ, Yealy DM. Emergency medicine journal impact factor and change compared to other medical and surgical specialties. Academic emergency medicine : official journal of the Society for Academic Emergency Medicine. 2012;19(11):1248–54.
244. Macdonald S. Emperor’s New Clothes: The Reinvention of Peer Review as Myth. JOURNAL OF MANAGEMENT INQUIRY. 2015 Jul;24(3):264–279.
245. Miguel Campanario J. Empirical study of journal impact factors obtained using the classical two-year citation window versus a five-year citation window. Scientometrics. 2011 Apr;87(1):189–204.
246. Song M, Kim S, Lee K. Ensemble analysis of topical journal ranking in bioinformatics. JOURNAL OF THE ASSOCIATION FOR INFORMATION SCIENCE AND TECHNOLOGY. 2017 Jun;68(6):1564–1583.
247. Ketefian S, Dai Y-T. Environment for nursing scholarship and journal impact factors in Taiwan. Nursing & health sciences. 2010;12(2):191–7.
248. Ketefian S, Dai Y-T, Hanucharurnkul S, Mendes IAC, Norman IJ. Environments for nursing scholarship and journal impact factor in five countries. International nursing review. 2010;57(3):343–51.
249. Ryan C, Tewey B, Newman S, Turner T, Jaeger RJ. Estimating research productivity and quality in assistive technology: a bibliometric analysis spanning four decades. IEEE transactions on neural systems and rehabilitation engineering : a publication of the IEEE Engineering in Medicine and Biology Society. 2004;12(4):422–9.
250. Chen K-M, Jen T-H, Wu M. Estimating the accuracies of journal impact factor through bootstrap. JOURNAL OF INFORMETRICS. 2014 Jan;8(1):181–196.
251. Prathap G. Evaluating journal performance metrics. Scientometrics. 2012 Aug;92(2, SI):403–408.
252. Mingers J, Yang L. Evaluating journal quality: A review of journal citation indicators, and ranking in business

and management. EUROPEAN JOURNAL OF OPERATIONAL RESEARCH. 2017 Feb;257(1):323–337.
253. Rosas SR, Kagan JM, Schouten JT, Slack PA, Trochim WMK. Evaluating research and impact: a bibliometric analysis of research by the NIH/NIAID HIV/AIDS clinical trials networks. PloS one. 2011;6(3):e17428.
254. Klein-Fedyshin M, Ketchum AM, Arnold RM, Fedyshin PJ. Evaluating the MEDLINE Core Clinical Journals filter: data-driven evidence assessing clinical utility. Journal of evaluation in clinical practice. 2014;20(6):837–43.
255. Sosa JA, Mehta P, Thomas DC, Berland G, Gross C, McNamara RL, et al. Evaluating the surgery literature: can standardizing peer-review today predict manuscript impact tomorrow?. Annals of surgery. 2009;250(1):152–8.
256. Herrmann-Lingen C, Brunner E, Hildenbrand S, Loew TH, Raupach T, Spies C, et al. Evaluation of medical research performance–position paper of the Association of the Scientific Medical Societies in Germany (AWMF). German medical science : GMS e-journal. 2014;12(101227686):Doc11.
257. Gross Cohn E, Haomiao Jia, Larson E. Evaluation of statistical approaches in quantitative nursing research. Clinical nursing research. 2009;18(3):223–41.
258. Ingwersen P, Larsen B. Evaluation of strategic research programs: the case of Danish environmental research 1993-2002. RESEARCH EVALUATION. 2007 Mar;16(1):47–57.
259. Dasi F, Navarro-Garcia MM, Jimenez-Heredia M, Magraner J, Vina JR, Pallardo FV, et al. Evaluation of the quality of publications on randomized clinical trials using the Consolidated Standards of Reporting Trials (CONSORT) statement guidelines in a Spanish tertiary hospital. Journal of clinical pharmacology. 2012;52(7):1106–14.
260. Dechartres A, Trinquart L, Atal I, Moher D, Dickersin K, Boutron I, et al. Evolution of poor reporting and inadequate methods over time in 20920 randomised controlled trials included in Cochrane reviews: research on research study. BMJ (Clinical research ed). 2017;357(8900488, bmj, 101090866):j2490.
261. Malesios C, Abas Z. Examination of the impact of animal and dairy science journals based on traditional and newly developed bibliometric indices. Journal of animal science. 2012;90(13):5170–81.
262. Kurmis AP, Kurmis TP. Exploring the relationship between impact factor and manuscript rejection rates in radiologic journals. Academic radiology. 2006;13(1):77–83.
263. Lu K, Ajiferuke I, Wolfram D. Extending citer analysis to journal impact evaluation. Scientometrics. 2014 Jul;100(1):245–260.
264. Vanclay JK. Factors affecting citation rates in environmental science. JOURNAL OF INFORMETRICS. 2013;7(2):265–271.
265. Sewell JM, Adejoro OO, Fleck JR, Wolfson JA, Konety BR. Factors associated with the Journal Impact Factor (JIF) for Urology and Nephrology Journals. International braz j urol : official journal of the Brazilian Society of Urology. 2015;41(6):1058–66.
266. Manriquez J, Cataldo K, Harz I. Factors influencing citations to systematic reviews in skin diseases: a cross-sectional study through Web of Sciences and Scopus. Anais brasileiros de dermatologia. 2015;90(5):646–52.
267. Lin M-H, Hwang S-J, Hwang I-H, Chen Y-C. Family medicine publications in Taiwan: An analysis of the Web of Science database from 1993 to 2012. JOURNAL OF THE CHINESE MEDICAL ASSOCIATION. 2014 Nov;77(11):583–588.
268. McDonald RJ, Cloft HJ, Kallmes DF. Fate of manuscripts previously rejected by the American Journal of Neuroradiology: a follow-up analysis. AJNR American journal of neuroradiology. 2009;30(2):253–6.
269. Pares D, Norton C, Chelvanayagam S. Fecal incontinence: The quality of reported randomized, controlled trials in the last ten years. DISEASES OF THE COLON & RECTUM. 2008 Jan;51(1):88–95.
270. Voleti PB, Tjoumakaris FP, Rotmil G, Freedman KB. Fifty most-cited articles in anterior cruciate ligament research. Orthopedics. 2015;38(4):e297–304.
271. Ioannidis JPA, Belbasis L, Evangelou E. Fifty-year fate and impact of general medical journals. PloS one. 2010;5(9).
272. Williams JK, Tripp-Reimer T, Daack-Hirsch S, DeBerg J. Five-Year Bibliometric Review of Genomic Nursing Science Research. JOURNAL OF NURSING SCHOLARSHIP. 2016 Mar;48(2):179–186.
273. Jacso P. Five-year impact factor data in the Journal Citation Reports. ONLINE INFORMATION REVIEW. 2009;33(3):603–614.
274. Fiocchi A, Assa'ad A, Bahna S, Adverse Reactions to Foods Committee, American College of Allergy A and

I. Food allergy and the introduction of solid foods to infants: a consensus document. Adverse Reactions to Foods Committee, American College of Allergy, Asthma and Immunology. Annals of allergy, asthma & immunology : official publication of the American College of Allergy, Asthma, & Immunology. 2006;97(1):10–77.

275. Soh N, Walter G, Touyz S, Russell J, Malhi GS, Hunt GE. Food for thought: comparison of citations received from articles appearing in specialized eating disorder journals versus general psychiatry journals. The International journal of eating disorders. 2012;45(8):990–4.
276. Cole GD, Nowbar AN, Mielewczik M, Shun-Shin MJ, Francis DP. Frequency of discrepancies in retracted clinical trial reports versus unretracted reports: blinded case-control study. BMJ (Clinical research ed). 2015;351(8900488, bmj, 101090866):h4708.
277. Czorlich P, Regelsberger J, Meixensberger J, Westphal M, Eicker SO. From Abstract to Publication in a Peer-Reviewed Journal: Evaluation of the 63rd Annual Meeting of the German Society of Neurosurgery. Journal of neurological surgery Part A, Central European neurosurgery. 2016;77(1):46–51.
278. Heneberg P. From Excessive Journal Self-Cites to Citation Stacking: Analysis of Journal Self-Citation Kinetics in Search for Journals, Which Boost Their Scientometric Indicators. PloS one. 2016;11(4):e0153730.
279. Maas E, Maher C, Moseley A, Annevelink R, Jagersma J, Ostelo R. Funding is related to the quality, conduct, and reporting of trial reports in musculoskeletal physical therapy: A survey of 210 published trials. Physiotherapy theory and practice. 2016;32(8):628–635.
280. Hammarstrom A, Lehti A, Danielsson U, Bengs C, Johansson EE. Gender-related explanatory models of depression: a critical evaluation of medical articles. Public health. 2009;123(10):689–93.
281. van Rossum M, Bosker BH, Pierik EGJM, Verheyen CCPM. Geographic origin of publications in surgical journals. The British journal of surgery. 2007;94(2):244–7.
282. Frandsen T. Geographical concentration - The case of economics journals. Scientometrics. 2005 Apr;63(1):69–85.
283. Jacso P. Grim tales about the impact factor and the h-index in the Web of Science and the Journal Citation Reports databases: reflections on Vanclay's criticism. Scientometrics. 2012 Aug;92(2, SI):325–354.
284. Toews I, Binder N, Wolff RF, Toprak G, von Elm E, Meerpohl JJ. Guidance in author instructions of hematology and oncology journals: A cross sectional and longitudinal study. PloS one. 2017;12(4):e0176489.
285. Ahn CS, Li RJ, Ahn BS, Kuo P, Bryant J, Day CS. Hand and wrist research productivity in journals with high impact factors: a 20 year analysis. The Journal of hand surgery, European volume. 2012;37(3):275–83.
286. Vera-Badillo FE, Napoleone M, Krzyzanowska MK, Alibhai SMH, Chan A-W, Ocana A, et al. Honorary and ghost authorship in reports of randomised clinical trials in oncology. EUROPEAN JOURNAL OF CANCER. 2016 Oct;66:1–8.
287. Al-Herz W, Haider H, Al-Bahhar M, Sadeq A. Honorary authorship in biomedical journals: how common is it and why does it exist? JOURNAL OF MEDICAL ETHICS. 2014 May;40(5):346–348.
288. Leydesdorff L. How are New Citation-Based Journal Indicators Adding to the Bibliometric Toolbox? JOURNAL OF THE AMERICAN SOCIETY FOR INFORMATION SCIENCE AND TECHNOLOGY. 2009 Jul;60(7):1327–1336.
289. Ha L, Yang C, Fang L, Zhang T, Chattopadhyay D, Wang F. How Media Scholars' Attributes Affect Their Ratings of Journalism and Mass Communication Quarterly. JOURNALISM & MASS COMMUNICATION QUARTERLY. 2015 Mar;92(1):221–234.
290. von Bohlen Und Halbach O. How to judge a book by its cover? How useful are bibliometric indices for the evaluation of "scientific quality" or "scientific productivity"?. Annals of anatomy = Anatomischer Anzeiger : official organ of the Anatomische Gesellschaft. 2011;193(3):191–6.
291. Lokker C, Haynes RB, Chu R, McKibbon KA, Wilczynski NL, Walter SD. How well are journal and clinical article characteristics associated with the journal impact factor? a retrospective cohort study. Journal of the Medical Library Association : JMLA. 2012;100(1):28–33.
292. Hansen HB, Henriksen JH. How well does journal "impact" work in the assessment of papers on clinical physiology and nuclear medicine?. Clinical physiology (Oxford, England). 1997;17(4):409–18.
293. Liu Y-H, Wang S-Q, Xue J-H, Liu Y, Chen J-Y, Li G-F, et al. Hundred top-cited articles focusing on acute kidney injury: a bibliometric analysis. BMJ open. 2016;6(7):e011630.

294. Ringelhan S, Wollersheim J, Welpe IM. I Like, I Cite? Do Facebook Likes Predict the Impact of Scientific Work?. PloS one. 2015;10(8):e0134389.
295. Vaughan CP, Fowler R, Goodman RA, Graves TR, Flacker JM, Johnson TM 2nd. Identifying landmark articles for advancing the practice of geriatrics. Journal of the American Geriatrics Society. 2014;62(11):2159–62.
296. Schloegl C, Stock W. Impact and relevance of LIS journals: A scientometric analysis of international and German-language LIS journals - Citation analysis versus reader survey. JOURNAL OF THE AMERICAN SOCIETY FOR INFORMATION SCIENCE AND TECHNOLOGY. 2004 Nov;55(13):1155–1168.
297. Wolf DM, Williamson PA. Impact factor and study design: the Academic Value of Published Research (AVaRes) score. Annals of the Royal College of Surgeons of England. 2009;91(1):71–3.
298. Tellez-Zenteno JF, Morales-Buenrostro LE, Estanol B. Impact factor of Latin American medical journals. REVISTA MEDICA DE CHILE. 2007 Apr;135(4):480–487.
299. Azer SA, Holen A, Wilson I, Skokauskas N. Impact factor of medical education journals and recently developed indices: Can any of them support academic promotion criteria? JOURNAL OF POSTGRADUATE MEDICINE. 2016 Mar;62(1):32–39.
300. Grzybowski A. Impact factor–strengths and weaknesses. Clinics in dermatology. 2010;28(4):455–7.
301. Polit DF, Northam S. Impact factors in nursing journals. Nursing outlook. 2011;59(1):18–28.
302. Moverley R, Rankin KS, McNamara I, Davidson DJ, Reed M, Sprowson AP. Impact factors of orthopaedic journals between 2000 and 2010: trends and comparisons with other surgical specialties. International orthopaedics. 2013;37(4):561–7.
303. Dorta-Gonzalez P, Dorta-Gonzalez MI. Impact maturity times and citation time windows: The 2-year maximum journal impact factor. JOURNAL OF INFORMETRICS. 2013;7(3):593–602.
304. Ivers NM, Taljaard M, Dixon S, Bennett C, McRae A, Taleban J, et al. Impact of CONSORT extension for cluster randomised trials on quality of reporting and study methodology: review of random sample of 300 trials, 2000-8. BMJ-BRITISH MEDICAL JOURNAL. 2011 Sep;343.
305. Guerrero-Bote VP, Zapico-Alonso F, Espinosa-Calvo ME, Gomez-Crisostomo R, De Moya-Anegon F. Import-export of knowledge between scientific subject categories: The iceberg hypothesis. Scientometrics. 2007 Jun;71(3):423–441.
306. Margalida A, Angels Colomer M. Improving the peer-review process and editorial quality: key errors escaping the review and editorial process in top scientific journals. PEERJ. 2016 Feb;4.
307. Oksvold MP. Incidence of Data Duplications in a Randomly Selected Pool of Life Science Publications. Science and engineering ethics. 2016;22(2):487–96.
308. Teixeira MC, Thomaz SM, Michelan TS, Mormul RP, Meurer T, Fasolli JVB, et al. Incorrect citations give unfair credit to review authors in ecology journals. PloS one. 2013;8(12):e81871.
309. Chow DS, Ha R, Filippi CG. Increased rates of authorship in radiology publications: a bibliometric analysis of 142,576 articles published worldwide by radiologists between 1991 and 2012. AJR American journal of roentgenology. 2015;204(1):W52–7.
310. Papatheodorou SI, Trikalinos TA, Ioannidis JPA. Inflated numbers of authors over time have not been just due to increasing research complexity. Journal of clinical epidemiology. 2008;61(6):546–51.
311. Lira RPC, Vieira RMC, Goncalves FA, Ferreira MCA, Maziero D, Passos THM, et al. Influence of English language in the number of citations of articles published in Brazilian journals of ophthalmology. Arquivos brasileiros de oftalmologia. 2013;76(1):26–8.
312. Sun GH, Houlton JJ, MacEachern MP, Bradford CR, Hayward RA. Influence of study sponsorship on head and neck cancer randomized trial results. HEAD AND NECK-JOURNAL FOR THE SCIENCES AND SPECIALTIES OF THE HEAD AND NECK. 2013 Oct;35(10):1515–1520.
313. Gu Y. Information management or knowledge management? An informetric view of the dynamics of Academia. Scientometrics. 2004;61(3):285–299.
314. Rohra DK, Rohra VK, Cahusac P. Institute for Scientific Information-indexed biomedical journals of Saudi Arabia. Their performance from 2007-2014. Saudi medical journal. 2016;37(11):1251–1257.
315. Rizkallah J, Sin DD. Integrative approach to quality assessment of medical journals using impact factor, eigenfactor, and article influence scores. PloS one. 2010;5(4):e10204.
316. Yuret T. Interfield comparison of academic output by using department level data. Scientometrics. 2015 Dec;105(3):1653–1664.

317. Nguyen TV, Ho-Le TP, Le UV. International collaboration in scientific research in Vietnam: an analysis of patterns and impact. Scientometrics. 2017 Feb;110(2):1035–1051.
318. Figueredo E, Sanchez Perales G, Munoz Blanco F. International publishing in anaesthesia - how do different countries contribute?. Acta anaesthesiologica Scandinavica. 2003;47(4):378–82.
319. Lehman VT, Doolittle DA, Hunt CH, Eckel LJ, Black DF, Schwartz KM, et al. Intracranial imaging of uncommon diseases is more frequently reported in clinical publications than in radiology publications. AJNR American journal of neuroradiology. 2014;35(1):45–8.
320. Vaughan L, Tang J, Yang R. Investigating disciplinary differences in the relationships between citations and downloads. Scientometrics. 2017 Jun;111(3):1533–1545.
321. Ashrafi F, Mohammadhassanzadeh H, Shokraneh F, Valinejadi A, Johari K, Saemi N, et al. Iranians' contribution to world literature on neuroscience. Health information and libraries journal. 2012;29(4):323–32.
322. Sanchez-Azanza VA, Lopez-Penades R, Buil-Legaz L, Aguilar-Mediavilla E, Adrover-Roig D. Is bilingualism losing its advantage? A bibliometric approach. PloS one. 2017;12(4):e0176151.
323. Crowe M, Carlyle D. Is open access sufficient? A review of the quality of open-access nursing journals. INTERNATIONAL JOURNAL OF MENTAL HEALTH NURSING. 2015 Feb;24(1):59–64.
324. Bornmann L, Wallon G, Ledin A. Is the h index related to (standard) bibliometric measures and to the assessments by peers? An investigation of the h index by using molecular life sciences data. RESEARCH EVALUATION. 2008 Jun;17(2):149–156.
325. Oliveira EA, Peicots-Filho R, Martelli DR, Quirino IG, Lopes Oliveira MC, Duarte MG, et al. Is there a correlation between journal impact factor and researchers' performance? A study comprising the fields of clinical nephrology and neurosciences. Scientometrics. 2013 Nov;97(2):149–160.
326. Wang J, Shapira P. Is there a relationship between research sponsorship and publication impact? An analysis of funding acknowledgments in nanotechnology papers. PloS one. 2015;10(2):e0117727.
327. Dilauro M, McInnes MDF, Korevaar DA, van der Pol CB, Petrcich W, Walther S, et al. Is There an Association between STARD Statement Adherence and Citation Rate? RADIOLOGY. 2016 Jul;280(1):62–67.
328. Abdullah L, Davis DE, Fabricant PD, Baldwin K, Namdari S. Is There Truly "No Significant Difference"? Underpowered Randomized Controlled Trials in the Orthopaedic Literature. JOURNAL OF BONE AND JOINT SURGERY-AMERICAN VOLUME. 2015 Dec;97A(24):2068–2073.
329. Micheli A, Di Salvo F, Lombardo C, Ciampichini R, Ugolini D, Baili P, et al. Italian performance in cancer research. Tumori. 2009;95(2):133–41.
330. Frandsen T. Journal diffusion factors - a measure of diffusion? ASLIB PROCEEDINGS. 2004;56(1):5–11.
331. Ni C, Shaw D, Lind SM, Ding Y. Journal impact and proximity: An assessment using bibliographic features. JOURNAL OF THE AMERICAN SOCIETY FOR INFORMATION SCIENCE AND TECHNOLOGY. 2013 Apr;64(4):802–817.
332. Beatty MJ, Feeley TH, Dodd MD. Journal impact factor or intellectual influence? A content analysis of citation use in Communication Monographs and Human Communication Research (2007-2009). PUBLIC RELATIONS REVIEW. 2012 Mar;38(1):174–176.
333. Paulus FM, Rademacher L, Schafer TAJ, Muller-Pinzler L, Krach S. Journal Impact Factor Shapes Scientists' Reward Signal in the Prospect of Publication. PloS one. 2015;10(11):e0142537.
334. Garfield E, Pudovkin A. Journal Impact Factor Strongly Correlates with the Citedness of the Median Journal Paper. COLLNET JOURNAL OF Scientometrics AND INFORMATION MANAGEMENT. 2015 Jun;9(1):5–14.
335. Rodrigues MA, Tedesco ACB, Nahas FX, Ferreira LM. Journal Impact Factor versus the Evidence Level of Articles Published in Plastic Surgery Journals. PLASTIC AND RECONSTRUCTIVE SURGERY. 2014 Jun;133(6):1502–1507.
336. Liu X-L, Gai S-S, Zhou J. Journal Impact Factor: Do the Numerator and Denominator Need Correction?. PloS one. 2016;11(3):e0151414.
337. Xue-li L, Mei-ying W, Lin Z, Pu W, Zhi-xin Z. Journal impact factor: is it only used in China and South Asia? CURRENT SCIENCE. 2013 Dec;105(11):1480–1484.
338. Bird SB. Journal impact factors, h indices, and citation analyses in toxicology. Journal of medical toxicology : official journal of the American College of Medical Toxicology. 2008;4(4):261–74.

339. Murtaugh P. Journal quality, effect size, and publication bias in meta-analysis. ECOLOGY. 2002 Apr;83(4):1162–1166.
340. Xiao-dong X, Ya-wen W. Journal Self-citation Analysis of Some Chinese Sci-tech Periodicals. SERIALS REVIEW. 2011 Sep;37(3):171–173.
341. Frandsen TF. Journal self-citations - Analysing the JIF mechanism. JOURNAL OF INFORMETRICS. 2007 Jan;1(1):47–58.
342. Dorta-Gonzalez P, Isabel Dorta-Gonzalez M, Rosa Santos-Penate D, Suarez-Vega R. Journal topic citation potential and between-field comparisons: The topic normalized impact factor. JOURNAL OF INFORMETRICS. 2014 Apr;8(2):406–418.
343. Kumaran M, Ha C. Knowledge of journal impact factors among nursing faculty: a cross-sectional study. Journal of the Medical Library Association : JMLA. 2017;105(2):140–144.
344. Glynn RW, Lowery AJ, Scutaru C, O'Dwyer T, Keogh I. Laryngeal cancer: Quantitative and qualitative assessment of research output, 1945-2010. LARYNGOSCOPE. 2012 Sep;122(9):1967–1973.
345. Wupperman R, Davis R, Obremskey WT. Level of evidence in Spine compared to other orthopedic journals. Spine. 2007;32(3):388–93.
346. Amiri AR, Kanesalingam K, Cro S, Casey ATH. Level of evidence of clinical spinal research and its correlation with journal impact factor. SPINE JOURNAL. 2013 Sep;13(9):1148–1153.
347. Lau SL, Samman N. Levels of evidence and journal impact factor in oral and maxillofacial surgery. INTERNATIONAL JOURNAL OF ORAL AND MAXILLOFACIAL SURGERY. 2007 Jan;36(1):1–5.
348. Chew M, Villanueva EV, Van Der Weyden MB. Life and times of the impact factor: retrospective analysis of trends for seven medical journals (1994-2005) and their Editors' views. Journal of the Royal Society of Medicine. 2007;100(3):142–50.
349. Whipple EC, Dixon BE, McGowan JJ. Linking health information technology to patient safety and quality outcomes: a bibliometric analysis and review. INFORMATICS FOR HEALTH & SOCIAL CARE. 2013 Jan;38(1):1–14.
350. Hughes BB, Beas-Luna R, Barner AK, Brewitt K, Brumbaugh DR, Cerny-Chipman EB, et al. Long-Term Studies Contribute Disproportionately to Ecology and Policy. BIOSCIENCE. 2017 Mar;67(3):271–281.
351. Allen L, Jones C, Dolby K, Lynn D, Walport M. Looking for landmarks: the role of expert review and bibliometric analysis in evaluating scientific publication outputs. PloS one. 2009;4(6):e5910.
352. Poomkottayil D, Bornstein MM, Sendi P. Lost in translation: the impact of publication language on citation frequency in the scientific dental literature. Swiss medical weekly. 2011;141(d10, 100970884):w13148.
353. Doughty K, Rothman L, Johnston L, Le K, Wu J, Howard A. Low-income countries' orthopaedic information needs: challenges and opportunities. Clinical orthopaedics and related research. 2010;468(10):2598–603.
354. Royle P, Waugh N. Macular disease research in the United Kingdom 2011-2014: a bibliometric analysis of outputs, performance and coverage. BMC research notes. 2015;8(101462768):833.
355. Sanni SA, Zainab AN, Raj RG, Abrizah A. Measuring journal diffusion using periodic citation counts. MALAYSIAN JOURNAL OF LIBRARY & INFORMATION SCIENCE. 2014;19(1):23–36.
356. Sanni SA, Zainab AN. Measuring the influence of a journal using impact and diffusion factors. MALAYSIAN JOURNAL OF LIBRARY & INFORMATION SCIENCE. 2011 Aug;16(2):127–140.
357. Garcia Romero A, Navarrete Cortes J, Escudero C, Fernandez Lopez JA, Chaichio Moreno JA. Measuring the influence of clinical trials citations on several bibliometric indicators. Scientometrics. 2009 Sep;80(3):747–760.
358. Thonon F, Boulkedid R, Delory T, Rousseau S, Saghatchian M, van Harten W, et al. Measuring the outcome of biomedical research: a systematic literature review. PloS one. 2015;10(4):e0122239.
359. Selvaraj S, Borkar DS, Prasad V. Media coverage of medical journals: do the best articles make the news?. PloS one. 2014;9(1):e85355.
360. Cosco TD. Medical journals, impact and social media: an ecological study of the Twittersphere. CMAJ : Canadian Medical Association journal = journal de l'Association medicale canadienne. 2015;187(18):1353–7.
361. Nourbakhsh E, Nugent R, Wang H, Cevik C, Nugent K. Medical literature searches: a comparison of PubMed and Google Scholar. Health information and libraries journal. 2012;29(3):214–22.
362. Adhikari S, Blaivas M, Frrokaj I, Shostrom V. Meeting abstracts to published manuscripts: how does emergency ultrasound compare?. Journal of ultrasound in medicine : official journal of the American

Institute of Ultrasound in Medicine. 2011;30(9):1275–9.
363. Hadlaczky G, Hokby S, Mkrtchian A, Carli V, Wasserman D. Mental Health First Aid is an effective public health intervention for improving knowledge, attitudes, and behaviour: a meta-analysis. International review of psychiatry (Abingdon, England). 2014;26(4):467–75.
364. Gholami J, Ilghami R. Metadiscourse markers in biological research articles and journal impact factor: Non-native writers vs. native writers. Biochemistry and molecular biology education : a bimonthly publication of the International Union of Biochemistry and Molecular Biology. 2016;44(4):349–60.
365. Bridoux V, Moutel G, Roman H, Kianifard B, Michot F, Herve C, et al. Methodological and ethical quality of randomized controlled clinical trials in gastrointestinal surgery. Journal of gastrointestinal surgery : official journal of the Society for Surgery of the Alimentary Tract. 2012;16(9):1758–67.
366. Nicolau I, Ling D, Tian L, Lienhardt C, Pai M. Methodological and reporting quality of systematic reviews on tuberculosis. The international journal of tuberculosis and lung disease : the official journal of the International Union against Tuberculosis and Lung Disease. 2013;17(9):1160–9.
367. Simundic A-M, Nikolac N, Topic E. Methodological issues in genetic association studies of inherited thrombophilia: original report of recent practice. Clinical and applied thrombosis/hemostasis : official journal of the International Academy of Clinical and Applied Thrombosis/Hemostasis. 2009;15(3):327–33.
368. Thabut G, Estellat C, Boutron I, Samama C, Ravaud P. Methodological issues in trials assessing primary prophylaxis of venous thrombo-embolism. EUROPEAN HEART JOURNAL. 2006 Jan;27(2):227–236.
369. Yucha CB, Schneider BSP, Smyer T, Kowalski S, Stowers E. Methodological quality and scientific impact of quantitative nursing education research over 18 months. Nursing education perspectives. 2011;32(6):362–8.
370. Akcan D, Axelsson S, Bergh C, Davidson T, Rosen M. Methodological quality in clinical trials and bibliometric indicators: no evidence of correlations. Scientometrics. 2013 Jul;96(1):297–303.
371. Danilla S, Wasiak J, Searle S, Arriagada C, Pedreros C, Cleland H, et al. Methodological quality of randomised controlled trials in burns care. A systematic review. BURNS. 2009 Nov;35(7):956–961.
372. Remschmidt C, Wichmann O, Harder T. Methodological quality of systematic reviews on influenza vaccination. Vaccine. 2014;32(15):1678–84.
373. Lu Y, Yao Q, Gu J, Shen C. Methodological reporting of randomized clinical trials in respiratory research in 2010. Respiratory care. 2013;58(9):1546–51.
374. Wu D, Akl EA, Guyatt GH, Devereaux PJ, Brignardello-Petersen R, Prediger B, et al. Methodological survey of designed uneven randomization trials (DU-RANDOM): a protocol. Trials. 2014;15(101263253):33.
375. Kuroki LM, Allsworth JE, Peipert JF. Methodology and analytic techniques used in clinical research: associations with journal impact factor. Obstetrics and gynecology. 2009;114(4):877–84.
376. Xue-Li L, Shuang-Shuang G, Shi-Le Z. Methodology of calculation and structural analysis of Journal Impact Factor based on the Web of Science: A case study of Nature. MALAYSIAN JOURNAL OF LIBRARY & INFORMATION SCIENCE. 2016;21(2):83–92.
377. Boyack KW, Jordan P. Metrics associated with NIH funding: a high-level view. Journal of the American Medical Informatics Association : JAMIA. 2011;18(4):423–31.
378. Zitt M, Small H. Modifying the journal impact factor by fractional citation weighting: The audience factor. JOURNAL OF THE AMERICAN SOCIETY FOR INFORMATION SCIENCE AND TECHNOLOGY. 2008 Sep;59(11):1856–1860.
379. Krzych LJ, Liszka L. No improvement in studies reporting the diagnostic accuracy of B-type natriuretic peptide. Medical science monitor : international medical journal of experimental and clinical research. 2009;15(5):SR5–14.
380. Bjarnason T, Sigfusdottir I. Nordic impact: Article productivity and citation patterns in sixteen Nordic sociology departments. ACTA SOCIOLOGICA. 2002;45(4):253–267.
381. Owlia P, Vasei M, Goliaei B, Nassiri I. Normalized impact factor (NIF): an adjusted method for calculating the citation rate of biomedical journals. Journal of biomedical informatics. 2011;44(2):216–20.
382. Scotti V, De Silvestri A, Scudeller L, Abele P, Topuz F, Curti M. Novel bibliometric scores for evaluating research quality and output: a correlation study with established indexes. The International journal of biological markers. 2016;31(4):e451–e455.
383. Holguin JA. Occupational therapy and the journal citation reports: 10-year performance trajectories. The American journal of occupational therapy : official publication of the American Occupational Therapy

Association. 2009;63(1):105–12.
384. Leonard DS, Broe P. Oesophageal achalasia: An argument for primary surgical management. SURGEON-JOURNAL OF THE ROYAL COLLEGES OF SURGEONS OF EDINBURGH AND IRELAND. 2009 Apr;7(2):101–113.
385. Mansilla R, Koppen E, Cocho G, Miramontes P. On the behavior of journal impact factor rank-order distribution. JOURNAL OF INFORMETRICS. 2007 Apr;1(2):155–160.
386. Ghiasi G, Lariviere V, Sugimoto CR. On the Compliance of Women Engineers with a Gendered Scientific System. PloS one. 2015;10(12):e0145931.
387. Gumpenberger C, Ovalle-Perandones M-A, Gorraiz J. On the impact of Gold Open Access journals. Scientometrics. 2013 Jul;96(1):221–238.
388. Ebadi A, Schiffauerova A. On the relation between the small world structure and scientific activities. PloS one. 2015;10(3):e0121129.
389. Vasilevsky NA, Brush MH, Paddock H, Ponting L, Tripathy SJ, Larocca GM, et al. On the reproducibility of science: unique identification of research resources in the biomedical literature. PeerJ. 2013;1(101603425):e148.
390. Finardi U. On the time evolution of received citations, in different scientific fields: An empirical study. JOURNAL OF INFORMETRICS. 2014 Jan;8(1):13–24.
391. Todd PA, Guest JR, Lu J, Chou LM. One in four citations in marine biology papers is inappropriate. MARINE ECOLOGY PROGRESS SERIES. 2010;408:299–303.
392. Christensen F, Ingwersen P, Wormell I. Online determination of the journal impact factor and its international properties. Scientometrics. 1997 Dec;40(3):529–540.
393. Dougherty MC, Freda MC, Kearney MH, Baggs JG, Broome M. Online Survey of Nursing Journal Peer Reviewers: Indicators of Quality in Manuscripts. WESTERN JOURNAL OF NURSING RESEARCH. 2011 Jun;33(4):506–521.
394. Qi X, Li H, Liu X, Xu W, Bai M, Guo X. Online-to-print Lags and Baseline Number of Citations in 5 Science Citation Index Journals Related to Liver Diseases (2013-2014). Journal of clinical and experimental hepatology. 2015;5(2):127–33.
395. Wren J. Open access and openly accessible: a study of scientific publications shared via the internet. BRITISH MEDICAL JOURNAL. 2005 May;330(7500):1128–1131.
396. Niyazov Y, Vogel C, Price R, Lund B, Judd D, Akil A, et al. Open Access Meets Discoverability: Citations to Articles Posted to Academia.edu. PloS one. 2016;11(2):e0148257.
397. Baskin SM, Lin C, Carlson JN. Osteopathic emergency medicine programs infrequently publish in high-impact emergency medicine journals. The western journal of emergency medicine. 2014;15(7):908–12.
398. Basu S, Pollack MM. Outcome of Pediatric Critical Care Medicine Abstracts Presented at North American Academic National Meetings. Pediatric critical care medicine : a journal of the Society of Critical Care Medicine and the World Federation of Pediatric Intensive and Critical Care Societies. 2017;(100954653).
399. Compeau EA, Gordon K, Buys YM. Outcomes of Canadian National Institute for the Blind Baker research grants from 1998 to 2009. Canadian journal of ophthalmology Journal canadien d'ophtalmologie. 2011;46(5):386–90.
400. Silberzweig JE, Khorsandi AS. Outcomes of Rejected Journal of Vascular and Interventional Radiology Manuscripts. JOURNAL OF VASCULAR AND INTERVENTIONAL RADIOLOGY. 2008 Nov;19(11):1620–1623.
401. Onyeka TC, Chukwuneke FN. Pain research in Africa: a ten-year bibliometric survey. Journal of anesthesia. 2014;28(4):511–6.
402. Yang H, Zhang J-H, Zhang F. Papers featured in the World Journal of Gastroenterology from 2006 to 2007. World journal of gastroenterology. 2009;15(35):4471–5.
403. Khan NR, Thompson CJ, Taylor DR, Gabrick KS, Choudhri AF, Boop FR, et al. Part II: Should the h-index be modified? An analysis of the m-quotient, contemporary h-index, authorship value, and impact factor. World neurosurgery. 2013;80(6):766–74.
404. Li Z, Wan X, Lu A, Li X, Li J. Pathological research output in China and other top-ranking countries: 10-year survey of the literature. PATHOLOGY RESEARCH AND PRACTICE. 2010;206(12):835–838.
405. Gagliardi AR, Dobrow MJ. Paucity of qualitative research in general medical and health services and policy research journals: analysis of publication rates. BMC health services research. 2011;11(101088677):268.

406. Hefny AF, Grivna M, Abbas AK, Branicki FJ, Abu-Zidan FM. Pediatric trauma research in the Gulf Cooperation Council countries. Asian journal of surgery. 2012;35(2):74–80.
407. Greenberg D, Wacht O, Pliskin JS. Peer review in publication: factors associated with the full-length publication of studies presented in abstract form at the annual meeting of the Society for Medical Decision Making. Medical decision making : an international journal of the Society for Medical Decision Making. 2008;28(6):938–42.
408. Djuric D. Penetrating the omerta of predatory publishing: the romanian connection. Science and engineering ethics. 2015;21(1):183–202.
409. Danthi N, Wu CO, Shi P, Lauer M. Percentile ranking and citation impact of a large cohort of National Heart, Lung, and Blood Institute-funded cardiovascular R01 grants. Circulation research. 2014;114(4):600–6.
410. Michaelis LC, Ratain MJ. Phase II trials published in 2002: a cross-specialty comparison showing significant design differences between oncology trials and other medical specialties. Clinical cancer research : an official journal of the American Association for Cancer Research. 2007;13(8):2400–5.
411. Balaban AT. Positive and negative aspects of citation indices and journal impact factors. Scientometrics. 2012 Aug;92(2, SI):241–247.
412. Sune P, Sune JM, Montoro JB. Positive outcomes influence the rate and time to publication, but not the impact factor of publications of clinical trial results. PloS one. 2013;8(1):e54583.
413. Siontis GCM, Tzoulaki I, Ioannidis JPA. Predicting death: an empirical evaluation of predictive tools for mortality. Archives of internal medicine. 2011;171(19):1721–6.
414. Haslam N, Koval P. Predicting long-term citation impact of articles in social and personality psychology. Psychological reports. 2010;106(3):891–900.
415. Willis DL, Bahler CD, Neuberger MM, Dahm P. Predictors of citations in the urological literature. BJU INTERNATIONAL. 2011 Jun;107(12):1876–1880.
416. Moghaddam GG. Price and value of electronic journals: A survey at the Indian Institute of Science. LIBRI. 2006 Jun;56(2):108–116.
417. Oliveira EA de, Ribeiro ALP, Quirino IG, Oliveira MCL, Martelli DR, Lima LS, et al. Profile and scientific production of CNPq researchers in cardiology. Arquivos brasileiros de cardiologia. 2011;97(3):186–93.
418. Oliveira MCLA, Martelli DR, Quirino IG, Colosimo EA, Silva ACS e, Martelli Junior H, et al. Profile and scientific production of the Brazilian Council for Scientific and Technological Development (CNPq) researchers in the field of Hematology/Oncology. Revista da Associacao Medica Brasileira (1992). 2014;60(6):542–7.
419. Luchs A. Profile of Brazilian scientific production on A/H1N1 pandemic influenza. Ciencia & saude coletiva. 2012;17(6):1629–34.
420. Araujo CR, Moreira MA, Lana-Peixoto MA. Profile of the Brazilian scientific production in multiple sclerosis. Brazilian journal of medical and biological research = Revista brasileira de pesquisas medicas e biologicas. 2006;39(9):1143–8.
421. Fredriksson JJ, Ebbevi D, Savage C. Pseudo-understanding: an analysis of the dilution of value in healthcare. BMJ QUALITY & SAFETY. 2015 Jul;24(7):451–457.
422. Hunt GE, Cleary M, Walter G. Psychiatry and the Hirsch h-index: The relationship between journal impact factors and accrued citations. Harvard review of psychiatry. 2010;18(4):207–19.
423. Plikus MV, Zhang Z, Chuong C-M. PubFocus: semantic MEDLINE/PubMed citations analytics through integration of controlled biomedical dictionaries and ranking algorithm. BMC bioinformatics. 2006;7(100965194):424.
424. Piwowar HA, Chapman WW. Public sharing of research datasets: a pilot study of associations. Journal of informetrics. 2010;4(2):148–156.
425. Chou C-Y, Chew SS, Patel DV, Ormonde SE, McGhee CN. Publication and citation analysis of the Australian and New Zealand Journal of Ophthalmology and Clinical and Experimental Ophthalmology over a 10-year period: the evolution of an ophthalmology journal. Clinical & experimental ophthalmology. 2009;37(9):868–73.
426. van Vliet EPM, Eijkemans MJC, Kuipers EJ, Poley JW, Steyerberg EW, Siersema PD. Publication bias does not play a role in the reporting of the results of endoscopic ultrasound staging of upper gastrointestinal cancers. Endoscopy. 2007;39(4):325–32.
427. De Oliveira GSJ, Chang R, Kendall MC, Fitzgerald PC, McCarthy RJ. Publication bias in the anesthesiology

literature. Anesthesia and analgesia. 2012;114(5):1042–8.
428. Jamjoom AAB, Hughes MA, Chuen CK, Hammersley RL, Fouyas IP. Publication fate of abstracts presented at Society of British Neurological Surgeons meetings. British journal of neurosurgery. 2015;29(2):164–8.
429. Elangovan S, Allareddy V. Publication Metrics of Dental Journals - What is the Role of Self Citations in Determining the Impact Factor of Journals?. The journal of evidence-based dental practice. 2015;15(3):97–104.
430. Varghese RA, Chang J, Miyanji F, Reilly CW, Mulpuri K. Publication of abstracts submitted to the annual meeting of the Pediatric Orthopaedic Society of North America: is there a difference between accepted versus rejected abstracts?. Journal of pediatric orthopedics. 2011;31(3):334–40.
431. Crawford SA, Roche-Nagle G. Publication outcomes for research presented at a Canadian surgical conference. Canadian journal of surgery Journal canadien de chirurgie. 2017;60(2):108–114.
432. Amarilyo G, Woo JMP, Furst DE, Hoffman OL, Eyal R, Piao C, et al. Publication outcomes of abstracts presented at an American College of Rheumatology/Association of Rheumatology Health Professionals annual scientific meeting. Arthritis care & research. 2013;65(4):622–9.
433. Rabenda V, Bruyere O, Cooper C, Rizzoli R, Buckinx F, Quabron A, et al. Publication outcomes of the abstracts presented at the 2011 European Congress on Osteoporosis, Osteoarthritis and Musculo-Skeletal Diseases (ECCEO-IOF11): A position paper of the European Society for Clinical and Economical Aspects of Osteoporosis, Osteoarthritis and Musculo-Skeletal Diseases (ESCEO) and the International Osteoporosis and Other Skeletal Diseases Foundation (IOF). Archives of osteoporosis. 2015;10(101318988):11.
434. Bohm M, Papoutsis K, Gottwik M, Ukena C. Publication performance of women compared to men in German cardiology. International journal of cardiology. 2015;181(gqw, 8200291):267–9.
435. Sanni SA, Zainab AN. Publication productivity and citation analysis of the Medical Journal of Malaysia: 2004 - 2008. The Medical journal of Malaysia. 2012;67(1):52–9.
436. Meral UM, Alakus U, Urkan M, Ureyen O, Oren NC, Meral AO, et al. Publication Rate of Abstracts Presented at the Annual Congress of the European Society for Surgical Research during 2008-2011. EUROPEAN SURGICAL RESEARCH. 2016;56(3–4):132–140.
437. Meissner A, Delouya G, Marcovitch D, Donath D, Taussky D. Publication rates of abstracts presented at the 2007 and 2010 Canadian Association of Radiation Oncology meetings. Current oncology (Toronto, Ont). 2014;21(2):e250–4.
438. Egloff HM, West CP, Wang AT, Lowe KM, Edakkanambeth Varayil J, Beckman TJ, et al. Publication Rates of Abstracts Presented at the Society of General Internal Medicine Annual Meeting. Journal of general internal medicine. 2017;(8605834):673–678.
439. Rosing CK, Junges R, Haas AN. Publication rates of editorial board members in oral health journals. Brazilian oral research. 2014;28(100941949, 101307187).
440. Costa-Font J, McGuire A, Stanley T. Publication selection in health policy research: the winner's curse hypothesis. Health policy (Amsterdam, Netherlands). 2013;109(1):78–87.
441. Gordon D, Cooper-Arnold K, Lauer M. Publication Speed, Reporting Metrics, and Citation Impact of Cardiovascular Trials Supported by the National Heart, Lung, and Blood Institute. Journal of the American Heart Association. 2015;4(8):e002292.
442. Chen H, Chen CH, Jhanji V. Publication Times, Impact Factors, and Advance Online Publication in Ophthalmology Journals. OPHTHALMOLOGY. 2013 Aug;120(8):1697–1701.
443. Ma Y, Dong M, Zhou K, Mita C, Liu J, Wayne PM. Publication Trends in Acupuncture Research: A 20-Year Bibliometric Analysis Based on PubMed. PloS one. 2016;11(12):e0168123.
444. Ziemann E, Oestmann J-W. Publications by doctoral candidates at Charite University Hospital, Berlin, from 1998-2008. Deutsches Arzteblatt international. 2012;109(18):333–7.
445. Lauritsen J, Moller AM. Publications in anesthesia journals: quality and clinical relevance. Anesthesia and analgesia. 2004;99(5):1486–contents.
446. Franco Aixela J, Rovira-Esteva S. Publishing and impact criteria, and their bearing on Translation Studies: In search of comparability. PERSPECTIVES-STUDIES IN TRANSLATOLOGY. 2015 Apr;23(2, SI):265–283.
447. Royle J, Coles L, Williams D, Evans P. Publishing in international journals - An examination of trends in Chinese co-authorship. Scientometrics. 2007 Apr;71(1):59–86.
448. Poulin R. Qualitative and quantitative aspects of recent research on helminth parasites. Journal of

helminthology. 2002;76(4):373–6.
449. Bath FJ, Owen VE, Bath PM. Quality of full and final publications reporting acute stroke trials: a systematic review. Stroke. 1998;29(10):2203–10.
450. Roush GC, Amante B, Singh T, Ayele H, Araoye M, Yang D, et al. Quality of meta-analyses for randomized trials in the field of hypertension: a systematic review. Journal of hypertension. 2016;34(12):2305–2317.
451. Hussain JA, Bland M, Langan D, Johnson MJ, Currow DC, White IR. Quality of missing data reporting and handling in palliative care trials demonstrates that further development of the CONSORT missing data reporting guidance is required: a systematic review. Journal of clinical epidemiology. 2017;(jce, 8801383).
452. Cioffi I, Farella M. Quality of randomised controlled trials in dentistry. International dental journal. 2011;61(1):37–42.
453. Bausch B, Spaar A, Kleijnen J, Puhan MA. Quality of randomised trials in COPD. The European respiratory journal. 2009;34(5):1060–5.
454. Sjogren P, Halling A. Quality of reporting randomised clinical trials in dental and medical research. British dental journal. 2002;192(2):100–3.
455. Faggion CM Jr, Wu Y-C, Tu Y-K, Wasiak J. Quality of search strategies reported in systematic reviews published in stereotactic radiosurgery. BRITISH JOURNAL OF RADIOLOGY. 2016;89(1062).
456. Zhang C, Liu X, Xu Y (Calvin), Wang Y. Quality-Structure Index: A New Metric to Measure Scientific Journal Influence. JOURNAL OF THE AMERICAN SOCIETY FOR INFORMATION SCIENCE AND TECHNOLOGY. 2011 Apr;62(4):643–653.
457. Haslam N, Laham SM. Quality, quantity, and impact in academic publication. EUROPEAN JOURNAL OF SOCIAL PSYCHOLOGY. 2010 Mar;40(2):216–220.
458. Sutherland WJ, Goulson D, Potts SG, Dicks LV. Quantifying the impact and relevance of scientific research. PloS one. 2011;6(11):e27537.
459. Hessey R, Willett P. Quantifying the value of knowledge exports from librarianship and information science research. JOURNAL OF INFORMATION SCIENCE. 2013 Feb;39(1):141–150.
460. Rohra DK, Azam SI. Quantitative and Qualitative Analysis of PubMed-Indexed Biomedical Publications in Oman from years 2005-2009. Oman medical journal. 2011;26(3):160–5.
461. Mansouri A, Shin S, Cooper B, Srivastava A, Bhandari M, Kondziolka D. Randomized controlled trials and neuro-oncology: should alternative designs be considered? JOURNAL OF NEURO-ONCOLOGY. 2015 Sep;124(3):345–356.
462. Yanada M, Narimatsu H, Suzuki T, Matsuo K, Naoe T. Randomized controlled trials of treatments for hematologic malignancies: study characteristics and outcomes. Cancer. 2007;110(2):334–9.
463. Bala MM, Akl EA, Sun X, Bassler D, Mertz D, Mejza F, et al. Randomized trials published in higher vs. lower impact journals differ in design, conduct, and analysis. Journal of clinical epidemiology. 2013;66(3):286–95.
464. Racki G. Rank-normalized journal impact factor as a predictive tool. Archivum immunologiae et therapiae experimentalis. 2009;57(1):39–43.
465. Vanclay JK. Ranking forestry journals using the h-index. JOURNAL OF INFORMETRICS. 2008 Oct;2(4):326–334.
466. Moussa S, Touzani M. Ranking marketing journals using the Google Scholar-based hg-index. JOURNAL OF INFORMETRICS. 2010 Jan;4(1):107–117.
467. Jamal T, Smith B, Watson E. Ranking, rating and scoring of tourism journals: Interdisciplinary challenges and innovations. TOURISM MANAGEMENT. 2008 Feb;29(1):66–78.
468. Bar-Ilan J. Rankings of information and library science journals by JIF and by h-type indices. JOURNAL OF INFORMETRICS. 2010 Apr;4(2):141–147.
469. Abuzeid W, Fosbol EL, Fosbol PL, Fosbol M, Zarinehbaf S, Ross H, et al. Rate and predictors of the conversion of abstracts presented at the Canadian Cardiovascular Congress scientific meetings to full peer-reviewed publications. The Canadian journal of cardiology. 2013;29(11):1520–3.
470. Kiesslich T, Weineck SB, Koelblinger D. Reasons for Journal Impact Factor Changes: Influence of Changing Source Items. PloS one. 2016;11(4):e0154199.
471. Awrey J, Inaba K, Barmparas G, Recinos G, Teixeira PGR, Chan LS, et al. Reference Accuracy in the General Surgery Literature. WORLD JOURNAL OF SURGERY. 2011 Mar;35(3):475–479.
472. Dellavalle RP, Schilling LM, Rodriguez MA, Van de Sompel H, Bollen J. Refining dermatology journal

impact factors using PageRank. Journal of the American Academy of Dermatology. 2007;57(1):116–9.
473. Frost L, Grondal AK, Benjamin EJ, Friberg L, Rosenqvist M, Johnsen SP. Registry-based studies of atrial fibrillation from Sweden and Denmark, 2000-2014. SCANDINAVIAN CARDIOVASCULAR JOURNAL. 2016;50(5–6):323–328.
474. Jefferson T, Di Pietrantonj C, Debalini MG, Rivetti A, Demicheli V. Relation of study quality, concordance, take home message, funding, and impact in studies of influenza vaccines: systematic review. BRITISH MEDICAL JOURNAL. 2009 Feb;338.
475. Bain CR, Myles PS. Relationship between journal impact factor and levels of evidence in anaesthesia. Anaesthesia and intensive care. 2005;33(5):567–70.
476. Vaughan L, Hysen K. Relationship between links to journal Web sites and impact factors. ASLIB PROCEEDINGS. 2002;54(6):356–361.
477. Patsopoulos NA, Analatos AA, Ioannidis JPA. Relative citation impact of various study designs in the health sciences. JAMA. 2005;293(19):2362–6.
478. Hutchins BI, Yuan X, Anderson JM, Santangelo GM. Relative Citation Ratio (RCR): A New Metric That Uses Citation Rates to Measure Influence at the Article Level. PLOS BIOLOGY. 2016 Sep;14(9).
479. Fernando DM, Minton CAB. Relative Influence of Professional Counseling Journals. JOURNAL OF COUNSELING AND DEVELOPMENT. 2011;89(4):423–430.
480. Greenwood DC. Reliability of journal impact factor rankings. BMC medical research methodology. 2007;7(100968545):48.
481. Knox K, Adams J, Djulbegovic B, Stinson T, Tomori C, Bennett C. Reporting and dissemination of industry versus non-profit sponsored economic analyses of six novel drugs used in oncology. ANNALS OF ONCOLOGY. 2000 Dec;11(12):1591–1595.
482. Gambadauro P, Navaratnarajah R. Reporting of embryo transfer methods in IVF research: a cross-sectional study. Reproductive biomedicine online. 2015;30(2):137–43.
483. Hakoum MB, Anouti S, Al-Gibbawi M, Abou-Jaoude EA, Hasbani DJ, Lopes LC, et al. Reporting of financial and non-financial conflicts of interest by authors of systematic reviews: a methodological survey. BMJ open. 2016;6(8):e011997.
484. Jonsson U, Alaie I, Parling T, Arnberg FK. Reporting of harms in randomized controlled trials of psychological interventions for mental and behavioral disorders: A review of current practice. CONTEMPORARY CLINICAL TRIALS. 2014 May;38(1):1–8.
485. McRae A, Taljaard M, Weijer C, Bennett C, Skea Z, Boruch R, et al. Reporting of patient consent in healthcare cluster randomised trials is associated with the type of study interventions and publication characteristics. Journal of medical ethics. 2013;39(2):119–24.
486. Cisler JM, Barnes AC, Farnsworth D, Sifers SK. Reporting practices of dropouts in psychological research using a wait-list control: current state and suggestions for improvement. International journal of methods in psychiatric research. 2007;16(1):34–42.
487. Rikos D, Dardiotis E, Tsivgoulis G, Zintzaras E, Hadjigeorgiou GM. Reporting quality of randomized-controlled trials in multiple sclerosis from 2000 to 2015, based on CONSORT statement. Multiple sclerosis and related disorders. 2016;9(101580247):135–9.
488. Sankar P, Cho MK, Monahan K, Nowak K. Reporting Race and Ethnicity in Genetics Research: Do Journal Recommendations or Resources Matter?. Science and engineering ethics. 2015;21(5):1353–66.
489. Maclean EN, Stone IS, Ceelen F, Garcia-Albeniz X, Sommer WH, Petersen SE. Reporting standards in cardiac MRI, CT, and SPECT diagnostic accuracy studies: analysis of the impact of STARD criteria. EUROPEAN HEART JOURNAL-CARDIOVASCULAR IMAGING. 2014 Jun;15(6):691–700.
490. Zheng SL, Chan FT, Maclean E, Jayakumar S, Nabeebaccus AA. Reporting trends of randomised controlled trials in heart failure with preserved ejection fraction: a systematic review. Open heart. 2016;3(2):e000449.
491. Glynn RW, Chin JZ, Kerin MJ, Sweeney KJ. Representation of cancer in the medical literature–a bibliometric analysis. PloS one. 2010;5(11):e13902.
492. Rohra DK. Representation of less-developed countries in Pharmacology journals: an online survey of corresponding authors. BMC medical research methodology. 2011;11(100968545):60.
493. Nightingale JM, Marshall G. Reprint of "Citation analysis as a measure of article quality, journal influence and individual researcher performance". Nurse education in practice. 2013;13(5):429–36.
494. Vasilevsky NA, Minnier J, Haendel MA, Champieux RE. Reproducible and reusable research: are journal

data sharing policies meeting the mark?. PeerJ. 2017;5(101603425):e3208.
495. Marshall KE, Hammill TL. Research 101: An Initiative to Encourage and Facilitate Quality Resident Research in a Military Setting. Otolaryngology–head and neck surgery : official journal of American Academy of Otolaryngology-Head and Neck Surgery. 2017;156(6):1054–1059.
496. Shao J, Shen H. Research assessment and monetary rewards: the overemphasized impact factor in China. RESEARCH EVALUATION. 2012 Sep;21(3):199–203.
497. Schroen AT, Thielen MJ, Turrentine FE, Kron IL, Slingluff CL Jr. Research incentive program for clinical surgical faculty associated with increases in research productivity. JOURNAL OF THORACIC AND CARDIOVASCULAR SURGERY. 2012 Nov;144(5):1003–1009.
498. von Zglinicki T. Research on ageing in Germany. EXPERIMENTAL GERONTOLOGY. 2000 May;35(3):259–270.
499. Kalcioglu MT, Ileri Y, Karaca S, Egilmez OK, Kokten N. Research on the Submission, Acceptance and Publication Times of Articles Submitted to International Otorhinolaryngology Journals. Acta informatica medica : AIM : journal of the Society for Medical Informatics of Bosnia & Herzegovina : casopis Drustva za medicinsku informatiku BiH. 2015;23(6):379–84.
500. Dakik H, Kaidbey H, Sabra R. Research productivity of the medical faculty at the American University of Beirut. POSTGRADUATE MEDICAL JOURNAL. 2006 Jul;82(969):462–464.
501. Hon K-LE, Yong V, Leung T-F. Research statistics in Atopic Eczema: what disease is this? ITALIAN JOURNAL OF PEDIATRICS. 2012 Jun;38.
502. Albuquerque PC, Rodrigues ML. Research trends on pathogenic Cryptococcus species in the last 20 years: a global analysis with focus on Brazil. Future microbiology. 2012;7(3):319–29.
503. Layton DM, Clarke M. Research Waste: How Are Dental Survival Articles Indexed and Reported?. The International journal of oral & maxillofacial implants. 2016;31(1):125–32.
504. Yan J, MacDonald A, Baisi L-P, Evaniew N, Bhandari M, Ghert M. Retractions in orthopaedic research A SYSTEMATIC REVIEW. BONE & JOINT RESEARCH. 2016 Jun;5(6):263–268.
505. Tort ABL, Targino ZH, Amaral OB. Rising publication delays inflate journal impact factors. PloS one. 2012;7(12):e53374.
506. Lok CK, Chan MT, Martinson IM. Risk factors for citation errors in peer-reviewed nursing journals. Journal of advanced nursing. 2001;34(2):223–9.
507. Macleod MR, Lawson McLean A, Kyriakopoulou A, Serghiou S, de Wilde A, Sherratt N, et al. Risk of Bias in Reports of In Vivo Research: A Focus for Improvement. PLoS biology. 2015;13(10):e1002273.
508. Chess LE, Gagnier J. Risk of bias of randomized controlled trials published in orthopaedic journals. BMC medical research methodology. 2013;13(100968545):76.
509. Bohlin L, Esquivel AV, Lancichinetti A, Rosvall M. Robustness of Journal Rankings by Network Flows With Different Amounts of Memory. JOURNAL OF THE ASSOCIATION FOR INFORMATION SCIENCE AND TECHNOLOGY. 2016 Oct;67(10):2527–2535.
510. van Lent M, Overbeke J, Out HJ. Role of editorial and peer review processes in publication bias: analysis of drug trials submitted to eight medical journals. PloS one. 2014;9(8):e104846.
511. Mulward S, Gotzsche PC. Sample size of randomized double-blind trials 1976-1991. Danish medical bulletin. 1996;43(1):96–8.
512. Medeiros PM, Ladio AH, Albuquerque UP. Sampling problems in Brazilian research: a critical evaluation of studies on medicinal plants. REVISTA BRASILEIRA DE FARMACOGNOSIA-BRAZILIAN JOURNAL OF PHARMACOGNOSY. 2014 Apr;24(2):103–109.
513. Zhang L, Rousseau R, Sivertsen G. Science deserves to be judged by its contents, not by its wrapping: Revisiting Seglen's work on journal impact and research evaluation. PloS one. 2017;12(3):e0174205.
514. Kaifi JT, Kibbe MR, LeMaire SA, Staveley-O'Carroll KF, Kao LS, Sosa JA, et al. Scientific impact of Association for Academic Surgery and Society of University Surgeons plenary session abstracts increases in the era of the Academic Surgical Congress from 2006 to 2010. The Journal of surgical research. 2013;182(1):6–10.
515. Housri N, Cheung MC, Koniaris LG, Zimmers TA. Scientific impact of women in academic surgery. The Journal of surgical research. 2008;148(1):13–6.
516. Boyer R, Boutron I, Ravaud P. Scientific production and impact of national registers: the example of orthopaedic national registers. OSTEOARTHRITIS AND CARTILAGE. 2011 Jul;19(7):858–863.

517. Barrios M, Villarroya A, Borrego A. Scientific production in psychology: a gender analysis. Scientometrics. 2013 Apr;95(1):15–23.
518. Chang H-T, Lin M-H, Hwang I-H, Chen T-J, Lin H-C, Hou M-C, et al. Scientific publications in gastroenterology and hepatology in Taiwan: An analysis of Web of Science from 1993 to 2013. JOURNAL OF THE CHINESE MEDICAL ASSOCIATION. 2017 Feb;80(2):80–85.
519. Cubero P, Sanz O, Rodriguez M, Criado Y. Scope distribution analysis in the bulletin of the Spanish ceramic and glass society. BOLETIN DE LA SOCIEDAD ESPANOLA DE CERAMICA Y VIDRIO. 2005 Dec;44(6):427–434.
520. Leydesdorff L, Opthof T. Scopus's Source Normalized Impact per Paper (SNIP) Versus a Journal Impact Factor Based on Fractional Counting of Citations. JOURNAL OF THE AMERICAN SOCIETY FOR INFORMATION SCIENCE AND TECHNOLOGY. 2010 Nov;61(11):2365–2369.
521. Miguel Campanario J. Self-Citations That Contribute to the Journal Impact Factor: An Investment-Benefit-Yield Analysis. JOURNAL OF THE AMERICAN SOCIETY FOR INFORMATION SCIENCE AND TECHNOLOGY. 2010 Dec;61(12):2575–2580.
522. Gasparyan AY, Ayvazyan L, Akazhanov NA, Kitas GD. Self-correction in biomedical publications and the scientific impact. Croatian medical journal. 2014;55(1):61–72.
523. Gargouri Y, Hajjem C, Lariviere V, Gingras Y, Carr L, Brody T, et al. Self-selected or mandated, open access increases citation impact for higher quality research. PloS one. 2010;5(10):e13636.
524. Xia J, Nakanishi K. Self-selection and the citation advantage of open access articles. ONLINE INFORMATION REVIEW. 2012;36(1):40–51.
525. Tanaka LY, Herskovic JR, Iyengar MS, Bernstam EV. Sequential result refinement for searching the biomedical literature. Journal of biomedical informatics. 2009;42(4):678–84.
526. Piwowar HA, Day RS, Fridsma DB. Sharing detailed research data is associated with increased citation rate. PloS one. 2007;2(3):e308.
527. Mutz R, Daniel H-D. Skewed citation distributions and bias factors: Solutions to two core problems with the journal impact factor. JOURNAL OF INFORMETRICS. 2012 Apr;6(2):169–176.
528. Karimkhani C, Gamble R, Dellavalle RP. Social media impact factor: the top ten dermatology journals on Facebook and Twitter. Dermatology online journal. 2014;20(4):22327.
529. Brotons L. Species distribution models and impact factor growth in environmental journals: methodological fashion or the attraction of global change science. PloS one. 2014;9(11):e111996.
530. Peruzzi M, De Falco E, Abbate A, Biondi-Zoccai G, Chimenti I, Lotrionte M, et al. State of the Art on the Evidence Base in Cardiac Regenerative Therapy: Overview of 41 Systematic Reviews. BIOMED RESEARCH INTERNATIONAL. 2015;
531. Misra M, Golden NH, Katzman DK. State of the art systematic review of bone disease in anorexia nervosa. INTERNATIONAL JOURNAL OF EATING DISORDERS. 2016 Mar;49(3):276–292.
532. Evaniew N, van der Watt L, Bhandari M, Ghert M, Aleem I, Drew B, et al. Strategies to improve the credibility of meta-analyses in spine surgery: a systematic survey. SPINE JOURNAL. 2015 Sep;15(9):2066–2076.
533. Ramsdell R, Lerman J, Pickhardt D, Feldman D, Foster J, Houle TT. Subspecialty impact factors: the contribution of pediatric anesthesia and pain articles. Anesthesia and analgesia. 2009;108(1):105–10.
534. Cunha A, dos Santos B, Dias AM, Carmagnani AM, Lafer B, Busatto GF. Success in publication by graduate students in psychiatry in Brazil: an empirical evaluation of the relative influence of English proficiency and advisor expertise. BMC medical education. 2014;14(101088679):238.
535. Suiter AM, Moulaison HL. Supporting Scholars: An Analysis of Academic Library Websites' Documentation on Metrics and Impact. JOURNAL OF ACADEMIC LIBRARIANSHIP. 2015 Nov;41(6):814–820.
536. Jamjoom AAB, Phan PNH, Hutchinson PJ, Kolias AG. Surgical trainee research collaboratives in the UK: an observational study of research activity and publication productivity. BMJ open. 2016;6(2):e010374.
537. Shiga T, Wajima Z, Inoue T, Ogawa R. Survey of observer variation in transesophageal echocardiography: Comparison of anesthesiology and cardiology literature. JOURNAL OF CARDIOTHORACIC AND VASCULAR ANESTHESIA. 2003 Aug;17(4):430–442.
538. Miguel Campanario J, Molina A. Surviving bad times: The role of citations, self-citations and numbers of citable items in recovery of the journal impact factor after at least four years of continuous decreases. Scientometrics. 2009 Dec;81(3):859–864.

539. Housri N, Cheung MC, Gutierrez JC, Zimmers TA, Koniaris LG. SUS/AAS abstracts: what is the scientific impact?. Surgery. 2008;144(2):322–31.
540. Liu CC, Lui J, Paolucci EO, Rudmik L. Systematic Review of the Quality of Economic Evaluations in the Otolaryngology Literature. OTOLARYNGOLOGY-HEAD AND NECK SURGERY. 2015 Jan;152(1):106–115.
541. Huang M-H, Huang W-T, Chen D-Z. Technological impact factor: An indicator to measure the impact of academic publications on practical innovation. JOURNAL OF INFORMETRICS. 2014 Jan;8(1):241–251.
542. Karageorgopoulos DE, Lamnatou V, Sardi TA, Gkegkes ID, Falagas ME. Temporal trends in the impact factor of European versus USA biomedical journals. PloS one. 2011;6(2):e16300.
543. Madlock-Brown CR, Eichmann D. The (lack of) impact of retraction on citation networks. Science and engineering ethics. 2015;21(1):127–37.
544. Marx W. The anatomy of the International Journal of Materials Research in the light of bibliometry. INTERNATIONAL JOURNAL OF MATERIALS RESEARCH. 2009 Jan;100(1):11–23.
545. Mueller PS, Murali NS, Cha SS, Erwin PJ, Ghosh AK. The association between impact factors and language of general internal medicine journals. SWISS MEDICAL WEEKLY. 2006 Jul;136(27–28):441–443.
546. Smith DR. The continuing rise of contact dermatitis, Part 2: The scientific journal. Contact dermatitis. 2009;61(4):194–200.
547. Crookes PA, Reis SL, Jones SC. The development of a ranking tool for refereed journals in which nursing and midwifery researchers publish their work. NURSE EDUCATION TODAY. 2010 Jul;30(5):420–427.
548. Xie Z, Willett P. The development of computer science research in the People’s Republic of China 2000-2009: a bibliometric study. INFORMATION DEVELOPMENT. 2013 Aug;29(3):251–264.
549. Jones AW. The distribution of forensic journals, reflections on authorship practices, peer-review and role of the impact factor. FORENSIC SCIENCE INTERNATIONAL. 2007 Jan;165(2–3):115–128.
550. Goldsack J, McLaughlin C, Bristol MN, Loeb A, Bergey M, Sonnad SS. The distribution of outcomes research papers across clinical journals. Evaluation & the health professions. 2011;34(2):239–49.
551. Moreira JAG, Zeng XHT, Amaral LAN. The Distribution of the Asymptotic Number of Citations to Sets of Publications by a Researcher or from an Academic Department Are Consistent with a Discrete Lognormal Model. PloS one. 2015;10(11):e0143108.
552. Miguel Campanario J, Cabos W. The effect of additional citations in the stability of Journal Citation Report categories. Scientometrics. 2014 Feb;98(2):1113–1130.
553. Miguel Campanario J. The effect of citations on the significance of decimal places in the computation of journal impact factors. Scientometrics. 2014 May;99(2):289–298.
554. Froud R, Bjorkli T, Bright P, Rajendran D, Buchbinder R, Underwood M, et al. The effect of journal impact factor, reporting conflicts, and reporting funding sources, on standardized effect sizes in back pain trials: a systematic review and meta-regression. BMC MUSCULOSKELETAL DISORDERS. 2015 Nov;16.
555. Mueller PS, Murali NS, Cha SS, Erwin PJ, Ghosh AK. The effect of online status on the impact factors of general internal medicine journals. The Netherlands journal of medicine. 2006;64(2):39–44.
556. O’Kelly F, Nason GJ, Manecksha RP, Cascio S, Quinn FJ, Leonard M, et al. The effect of social media (#SoMe) on journal impact factor and parental awareness in paediatric urology. Journal of pediatric urology. 2017;(101233150).
557. Sillet A, Katsahian S, Range H, Czernichow S, Bouchard P. The EigenfactorTM Score in highly specific medical fields: the dental model. Journal of dental research. 2012;91(4):329–33.
558. Lee C-H, Shih C-P, Chang Y-C, Chaou C-H. The Evolution of Academic Performance in Emergency Medicine Journals: Viewpoint from 2000 to 2009 Journal Citation Reports. ACADEMIC EMERGENCY MEDICINE. 2011 Aug;18(8, SI):898–904.
559. Zhang Y, Kou J, Zhang X-G, Zhang L, Liu S-W, Cao X-Y, et al. The Evolution of Academic Performance in Nine Subspecialties of Internal Medicine: An Analysis of Journal Citation Reports from 1998 to 2010. PLOS ONE. 2012 Oct;7(10).
560. Ray J, Berkwits M, Davidoff F. The fate of manuscripts rejected by a general medical journal. AMERICAN JOURNAL OF MEDICINE. 2000 Aug;109(2):131–135.
561. de Meijer VE, Knops SP, van Dongen JA, Eyck BM, Vles WJ. The fate of research abstracts submitted to a national surgical conference: a cross-sectional study to assess scientific impact. American journal of surgery. 2016;211(1):166–71.

562. Mutz R, Daniel H-D. The generalized propensity score methodology for estimating unbiased journal impact factors. Scientometrics. 2012 Aug;92(2, SI):377–390.
563. Dreyer NA, Bryant A, Velentgas P. The GRACE Checklist: A Validated Assessment Tool for High Quality Observational Studies of Comparative Effectiveness. Journal of managed care & specialty pharmacy. 2016;22(10):1107–13.
564. Garfield E. The history and meaning of the journal impact factor. JAMA-JOURNAL OF THE AMERICAN MEDICAL ASSOCIATION. 2006 Jan;295(1):90–93.
565. Lancho-Barrantes BS, Guerrero-Bote VP, Moya-Anegon F. The iceberg hypothesis revisited. Scientometrics. 2010 Nov;85(2):443–461.
566. Yue W, Wilson C, Rousseau R. The immediacy index and the journal impact factor: Two highly correlated derived measures. CANADIAN JOURNAL OF INFORMATION AND LIBRARY SCIENCE-REVUE CANADIENNE DES SCIENCES DE L INFORMATION ET DE BIBLIOTHECONOMIE. 2004 Mar;28(1):33–48.
567. Gonzalez-Alcaide G, Carlos Valderrama-Zurian J, Aleixandre-Benavent R. The Impact Factor in non-English-speaking countries. Scientometrics. 2012 Aug;92(2, SI):297–311.
568. Kodumuri P, Ollivere B, Holley J, Moran CG. The impact factor of a journal is a poor measure of the clinical relevance of its papers. The bone & joint journal. 2014;96-B(3):414–9.
569. Chen M, Zhao M-H, Kallenberg CGM. The impact factor of rheumatology journals: an analysis of 2008 and the recent 10 years. Rheumatology international. 2011;31(12):1611–5.
570. Rezaei-Ghaleh N, Azizi F. The impact factor-based quality assessment of biomedical research institutes in Iran: effect of impact factor normalization by subject. Archives of Iranian medicine. 2007;10(2):182–9.
571. Jones AW. The impact of Alcohol and Alcoholism among substance abuse journals. Alcohol and alcoholism (Oxford, Oxfordshire). 1999;34(1):25–34.
572. Falagas ME, Zarkali A, Karageorgopoulos DE, Bardakas V, Mavros MN. The Impact of Article Length on the Number of Future Citations: A Bibliometric Analysis of General Medicine Journals. PLOS ONE. 2013 Feb;8(2).
573. Nabil S, Samman N. The impact of case reports in oral and maxillofacial surgery. International journal of oral and maxillofacial surgery. 2012;41(7):789–96.
574. Lyubarova R, Itagaki BK, Itagaki MW. The impact of National Institutes of Health funding on U.S. cardiovascular disease research. PloS one. 2009;4(7):e6425.
575. Lyubarova R, Itagaki BK, Itagaki MW. The Impact of National Institutes of Health Funding on US Cardiovascular Disease Research. PLOS ONE. 2009 Jul;4(7).
576. Marx W, Cardona M. The impact of Solid State Communications in view of the ISI Citation data. SOLID STATE COMMUNICATIONS. 2003 Jul;127(5):323–336.
577. Huang M-H, Lin W-YC. The influence of journal self-citations on journal impact factor and immediacy index. ONLINE INFORMATION REVIEW. 2012;36(5):639–654.
578. Yu G, Wang X, Yu D. The influence of publication delays on impact factors. Scientometrics. 2005 Aug;64(2):235–246.
579. Yu G, Guo R, Yu D. The influence of the publication delay on journal rankings according to the impact factor. Scientometrics. 2006 May;67(2):201–211.
580. Gluud LL, Sorensen TIA, Gotzsche PC, Gluud C. The journal impact factor as a predictor of trial quality and outcomes: cohort study of hepatobiliary randomized clinical trials. The American journal of gastroenterology. 2005;100(11):2431–5.
581. Grzybowski A. The journal impact factor: how to interpret its true value and importance. Medical science monitor : international medical journal of experimental and clinical research. 2009;15(2):SR1–4.
582. Ha TC, Tan SB, Soo KC. The journal impact factor: too much of an impact?. Annals of the Academy of Medicine, Singapore. 2006;35(12):911–6.
583. Satcher MJ, Litton AG, Waterbor JW, Brooks CM. The Journal of Cancer Education: a retrospective review of quality indicators. Journal of cancer education : the official journal of the American Association for Cancer Education. 2009;24(1):16–21.
584. Ma T, Wang G-F, Dong K, Cao M. The Journal's Integrated Impact Index: a new indicator for journal evaluation. Scientometrics. 2012 Feb;90(2):649–658.
585. Grandjean P, Eriksen ML, Ellegaard O, Wallin JA. The Matthew effect in environmental science publication:

a bibliometric analysis of chemical substances in journal articles. Environmental health : a global access science source. 2011;10(101147645):96.
586. Elizee PK, Ghassab RK, Raoofi A, Miri SM. The More Publication, the Higher Impact Factor: Citation Analysis of Top Nine Gastroenterology and Hepatology Journals. HEPATITIS MONTHLY. 2012 Dec;12(12).
587. Mariam N, Cavanna AE. The most cited works in Tourette syndrome. Journal of child neurology. 2012;27(10):1250–9.
588. Fraley RC, Vazire S. The N-pact factor: evaluating the quality of empirical journals with respect to sample size and statistical power. PloS one. 2014;9(10):e109019.
589. Kashkoush A, Prabhu AV, Tonetti D, Agarwal N. The Neurosurgery Match: A Bibliometric Analysis of 206 First-year Residents. World neurosurgery. 2017;(101528275).
590. Yamato TP, Maher C, Koes B, Moseley A. The PEDro scale had acceptably high convergent validity, construct validity, and interrater reliability in evaluating methodological quality of pharmaceutical trials. Journal of clinical epidemiology. 2017;(jce, 8801383).
591. Ingwersen P. The pragmatics of a diachronic journal impact factor. Scientometrics. 2012 Aug;92(2, SI):319–324.
592. Ketcham CM. The proper use of citation data in journal management. Archivum immunologiae et therapiae experimentalis. 2008;56(6):357–62.
593. Bornmann L, Marx W. The proposal of a broadening of perspective in evaluative bibliometrics by complementing the times cited with a cited reference analysis. JOURNAL OF INFORMETRICS. 2013 Jan;7(1):84–88.
594. Neuhaus C, Marx W, Daniel H-D. The Publication and Citation Impact Profiles of Angewandte Chemie and the Journal of the American Chemical Society Based on the Sections of Chemical Abstracts: A Case Study on the Limitations of the Journal Impact Factor. JOURNAL OF THE AMERICAN SOCIETY FOR INFORMATION SCIENCE AND TECHNOLOGY. 2009 Jan;60(1):176–183.
595. Lloyd JC, Madden-Fuentes RJ, Nelson CP, Kokorowski PJ, Wiener JS, Ross SS, et al. The Publication Ranking Score for pediatric urology: Quantifying thought leadership within the subspecialty. JOURNAL OF PEDIATRIC UROLOGY. 2013 Dec;9(6, B):1108–1113.
596. Pandis N, Fleming PS, Worthington H, Salanti G. The Quality of the Evidence According to GRADE Is Predominantly Low or Very Low in Oral Health Systematic Reviews. PloS one. 2015;10(7):e0131644.
597. Lee PH, Tse ACY. The quality of the reported sample size calculations in randomized controlled trials indexed in PubMed. EUROPEAN JOURNAL OF INTERNAL MEDICINE. 2017 May;40:16–21.
598. Nicolaisen J, Frandsen TF. The Reference Return Ratio. JOURNAL OF INFORMETRICS. 2008 Apr;2(2):128–135.
599. Waltman L, van Eck NJ. The Relation Between Eigenfactor, Audience Factor, and Influence Weight. JOURNAL OF THE AMERICAN SOCIETY FOR INFORMATION SCIENCE AND TECHNOLOGY. 2010 Jul;61(7):1476–1486.
600. Devereaux PJ, Manns BJ, Ghali WA, Quan H, Guyatt GH. The reporting of methodological factors in randomized controlled trials and the association with a journal policy to promote adherence to the Consolidated Standards of Reporting Trials (CONSORT) checklist. Controlled clinical trials. 2002;23(4):380–8.
601. Foo JYA. The retrospective analysis of bibliographical trends for nine biomedical engineering journals from 1999 to 2007. Annals of biomedical engineering. 2009;37(7):1474–81.
602. Schoffel N, Vitzthum K, Mache S, Groneberg DA, Quarcoo D. The role of endocarditis, myocarditis and pericarditis in qualitative and quantitative data analysis. International journal of environmental research and public health. 2009;6(12):2919–33.
603. Evaniew N, Adili AF, Ghert M, Khan M, Madden K, Smith C, et al. The Scholarly Influence of Orthopaedic Research According to Conventional and Alternative Metrics: A Systematic Review. JBJS reviews. 2017;5(5):e5.
604. Whipple EC, McGowan JJ, Dixon BE, Zafar A. The selection of high-impact health informatics literature: a comparison of results between the content expert and the expert searcher. Journal of the Medical Library Association : JMLA. 2009;97(3):212–8.
605. Thoma B, Sanders JL, Lin M, Paterson QS, Steeg J, Chan TM. The social media index: measuring the impact

of emergency medicine and critical care websites. The western journal of emergency medicine. 2015;16(2):242–9.
606. Sabnis AB, Diwan AD. The timing of surgery in lumbar disc prolapse: A systematic review. INDIAN JOURNAL OF ORTHOPAEDICS. 2014 Apr;48(2):127–135.
607. Shuaib W, Acevedo JN, Khan MS, Santiago LJ, Gaeta TJ. The top 100 cited articles published in emergency medicine journals. The American journal of emergency medicine. 2015;33(8):1066–71.
608. Del Fabbro M, Corbella S, Tsesis I, Taschieri S. The trend of quality of publications in endodontic surgery: a 10-year systematic survey of the literature. The journal of evidence-based dental practice. 2015;15(1):2–7.
609. Angarita AM, Stone R, Temkin SM, Levinson K, Fader AN, Tanner EJ. The Use of "Optimal Cytoreduction" Nomenclature in Ovarian Cancer Literature: Can We Move Toward a More Optimal Classification System? INTERNATIONAL JOURNAL OF GYNECOLOGICAL CANCER. 2016 Oct;26(8):1421–1427.
610. Theodoulou A, Bramwell DC, Spiteri AC, Kim SW, Krishnan J. The Use of Scoring Systems in Knee Arthroplasty: A Systematic Review of the Literature. JOURNAL OF ARTHROPLASTY. 2016 Oct;31(10):2364+.
611. Kelly BS, Redmond CE, Nason GJ, Healy GM, Horgan NA, Heffernan EJ. The Use of Twitter by Radiology Journals: An Analysis of Twitter Activity and Impact Factor. Journal of the American College of Radiology : JACR. 2016;13(11):1391–1396.
612. Bornmann L, Leydesdorff L. The validation of (advanced) bibliometric indicators through peer assessments: A comparative study using data from InCites and F1000. JOURNAL OF INFORMETRICS. 2013;7(2):286–291.
613. Kanaan Z, Galandiuk S, Abby M, Shannon KV, Dajani D, Hicks N, et al. The value of lesser-impact-factor surgical journals as a source of negative and inconclusive outcomes reporting. Annals of surgery. 2011;253(3):619–23.
614. Probst P, Huttner FJ, Klaiber U, Diener MK, Buchler MW, Knebel P. Thirty years of disclosure of conflict of interest in surgery journals. Surgery. 2015;157(4):627–33.
615. Pshetizky Y, Tandeter H, Tabenkin H, Vinker S, Lahad A, Karkabi K, et al. Thirty Years of Family Medicine Publications in Israel (1975-2004): What, Where, and How Much? JOURNAL OF THE AMERICAN BOARD OF FAMILY MEDICINE. 2009 Feb;22(1):57–61.
616. Lopez-Abente G, Munoz-Tinoco C. Time trends in the impact factor of Public Health journals. BMC public health. 2005;5(100968562):24.
617. Thorn JC, Noble SM, Hollingworth W. Timely and Complete Publication of Economic Evaluations Alongside Randomized Controlled Trials. PHARMACOECONOMICS. 2013;31(1):77–85.
618. Valachis A, Mauri D, Neophytou C, Polyzos NP, Tsali L, Garras A, et al. Translational Medicine and Reliability of Single-Nucleotide Polymorphism Studies: Can We Believe in SNP Reports or Not? INTERNATIONAL JOURNAL OF MEDICAL SCIENCES. 2011;8(6):492–500.
619. Aleixandre-Benavent R, Simon C, Fauser BCJM. Trends in clinical reproductive medicine research: 10 years of growth. Fertility and sterility. 2015;104(1):131–7.e5.
620. Ahmed AA, Holliday EB, Fakhreddine M, Yoo SK, Deville C, Jagsi R. Trends in Disclosures of Industry Sponsorship. INTERNATIONAL JOURNAL OF RADIATION ONCOLOGY BIOLOGY PHYSICS. 2016 Jul;95(4):1093–1101.
621. Cimmino MA, Maio T, Ugolini D, Borasi F, Mela GS. Trends in otolaryngology research during the period 1995-2000: a bibliometric approach. Otolaryngology–head and neck surgery : official journal of American Academy of Otolaryngology-Head and Neck Surgery. 2005;132(2):295–302.
622. Wells CI, Robertson JP, O'Grady G, Bissett IP. Trends in publication of general surgical research in New Zealand, 1996-2015. ANZ JOURNAL OF SURGERY. 2017 Feb;87(1–2):76–79.
623. Chua TC, Crowe PJ, Morris DL. Trends in Surgical Oncology Research in Australia During the Period 1998-2009-A Bibliometric Review. JOURNAL OF SURGICAL ONCOLOGY. 2011 Aug;104(2):216–219.
624. Grabas M, Dinulescu M, Droitcourt C, Dupuy A. Trial Designs and Characteristics in Laser Studies in Dermatology: A Systematic Review. DERMATOLOGIC SURGERY. 2017 Feb;43(2):198–203.
625. Falavigna A, Botelho RV, Teles AR, da Silva PG, Martins D, Guyot JP, et al. Twelve Years of Scientific Production on Medline by Latin American Spine Surgeons. PLOS ONE. 2014 Feb;9(2).
626. Peoples BK, Midway SR, Sackett D, Lynch A, Cooney PB. Twitter Predicts Citation Rates of Ecological Research. PLOS ONE. 2016 Nov;11(11).

627. Grimm LJ, Maxfield CM. Ultimate publication rate of unpublished manuscripts listed on radiology residency applications at one institution. Academic medicine : journal of the Association of American Medical Colleges. 2013;88(11):1719–22.
628. Kothari D, Gourevitch MN, Lee JD, Grossman E, Truncali A, Ark TK, et al. Undergraduate Medical Education in Substance Abuse: A Review of the Quality of the Literature. ACADEMIC MEDICINE. 2011 Jan;86(1):98–112.
629. Rousseau R. Updating the journal impact factor or total overhaul? Scientometrics. 2012 Aug;92(2, SI):413–417.
630. Prasad V, Goldstein JA. US News and World Report cancer hospital rankings: do they reflect measures of research productivity?. PloS one. 2014;9(9):e107803.
631. Haddad M. Use and relevance of bibliometrics for nursing. Nursing standard (Royal College of Nursing (Great Britain) : 1987). 2017;31(37):55–63.
632. Wren JD, Georgescu C, Giles CB, Hennessey J. Use it or lose it: citations predict the continued online availability of published bioinformatics resources. Nucleic acids research. 2017;45(7):3627–3633.
633. Garg V, Shen X, Cheng Y, Nawarskas JJ, Raisch DW. Use of Number Needed to Treat in Cost-Effectiveness Analyses. ANNALS OF PHARMACOTHERAPY. 2013 Mar;47(3):380–387.
634. Enger KB. Using citation analysis to develop core book collections in academic libraries. LIBRARY & INFORMATION SCIENCE RESEARCH. 2009 Apr;31(2):107–112.
635. Bernstam EV, Herskovic JR, Meric-Bernstam F. Using citation analysis to ease information overload in oncology. Journal of clinical oncology : official journal of the American Society of Clinical Oncology. 2005;23(16_suppl):6036.
636. Bernstam E, Herskovic J, Aphinyanaphongs Y, Aliferis C, Sriram M, Hersh W. Using citation data to improve retrieval from MEDLINE. JOURNAL OF THE AMERICAN MEDICAL INFORMATICS ASSOCIATION. 2006 Feb;13(1):96–105.
637. Worrall JL. Validating Peer Review in Criminal Justice Evaluation Research: Evidence from CrimeSolutions.gov. JOURNAL OF CRIMINAL JUSTICE EDUCATION. 2015 Oct;26(4):507–529.
638. Hirsch M, Duffy JMN, Kusznir JO, Davis CJ, Plana MN, Khan KS, et al. Variation in outcome reporting in endometriosis trials: a systematic review. AMERICAN JOURNAL OF OBSTETRICS AND GYNECOLOGY. 2016 Apr;214(4):452–464.
639. Rogozinska E, Fen Y, Molyneaux E, Khan KS, Thangaratinam S. Variation in outcomes in trials reporting effects of diet and lifestyle based intervention on pregnancy outcomes: a systematic review. Pregnancy hypertension. 2014;4(3):237.
640. Al Wattar BH, Placzek A, Troko J, Pirie AM, Khan KS, McCorry D, et al. Variation in the reporting of outcomes among pregnant women with epilepsy: a systematic review. EUROPEAN JOURNAL OF OBSTETRICS & GYNECOLOGY AND REPRODUCTIVE BIOLOGY. 2015 Dec;195:193–199.
641. Tirlapur SA, Riordain RN, Khan KS, Collaboration E-C. Variations in the reporting of outcomes used in systematic reviews of treatment effectiveness research in bladder pain syndrome. EUROPEAN JOURNAL OF OBSTETRICS & GYNECOLOGY AND REPRODUCTIVE BIOLOGY. 2014 Sep;180:61–67.
642. Jawas A, Hefny AF, Abbas AK, Abu-Zidan FM. Vascular surgery research in the Gulf Cooperation Council countries. Asian journal of surgery. 2014;37(2):100–5.
643. Spreckelsen C, Deserno TM, Spitzer K. Visibility of medical informatics regarding bibliometric indices and databases. BMC medical informatics and decision making. 2011;11(101088682):24.
644. Yan E, Ding Y. Weighted Citation: An Indicator of an Article's Prestige. JOURNAL OF THE AMERICAN SOCIETY FOR INFORMATION SCIENCE AND TECHNOLOGY. 2010 Aug;61(8):1635–1643.
645. Behrens TEJ, Fox P, Laird A, Smith SM. What is the most interesting part of the brain? TRENDS IN COGNITIVE SCIENCES. 2013 Jan;17(1):2–4.
646. Susarla SM, Mundinger GS, Swanson EW, Basile LE, Redett RJ, Dodson TB. What Is the Quality of the Evidence in the Craniomaxillofacial Surgery Literature? JOURNAL OF ORAL AND MAXILLOFACIAL SURGERY. 2015 Oct;73(10):2017–2023.
647. Gregory TN, Liu T, Machuk A, Arneja JS. What is the ultimate fate of presented abstracts? The conversion rates of presentations to publications over a five-year period from three North American plastic surgery meetings. The Canadian journal of plastic surgery = Journal canadien de chirurgie plastique. 2012;20(1):33–6.

648. Cheek J, Garnham B, Quan J. What's in a number? Issues in providing evidence of impact and quality of research(ers). QUALITATIVE HEALTH RESEARCH. 2006 Mar;16(3):423–435.
649. Didegah F, Thelwall M. Which factors help authors produce the highest impact research? Collaboration, journal and document properties. JOURNAL OF INFORMETRICS. 2013;7(4):861–873.
650. Steen RG, Casadevall A, Fang FC. Why Has the Number of Scientific Retractions Increased? PLOS ONE. 2013 Jul;8(7).
651. Gonon F, Konsman J-P, Cohen D, Boraud T. Why most biomedical findings echoed by newspapers turn out to be false: the case of attention deficit hyperactivity disorder. PloS one. 2012;7(9):e44275.
652. Vaughan L, Glanzel W, Korch C, Capes-Davis A. Widespread Use of Misidentified Cell Line KB (HeLa): Incorrect Attribution and Its Impact Revealed through Mining the Scientific Literature. Cancer research. 2017;77(11):2784–2788.
653. Pelts MD, Rolbiecki AJ, Albright DL. Wounded bonds: A review of the social work literature on gay, lesbian and bisexual military service members and veterans. JOURNAL OF SOCIAL WORK. 2015 Mar;15(2):207–220.
