# Supplementary material for: Evaluating Journal Impact Factor: a systematic survey of the pros and cons, and overview of alternative measures
Source: J Venom Anim Toxins Incl Trop Dis. 2020 Aug 31;26:e20190082. doi: 10.1590/1678-9199-JVATITD-2019-0082 (PMC7458102; doi:10.1590/1678-9199-JVATITD-2019-0082)

Journal of Venomous Animals and Toxins including Tropical Diseases
https://doi.org/10.1590/1678-9199-JVATITD-2019-0082

Supplementary Material to "Evaluating Journal Impact Factor: a systematic survey of the pros and cons, and overview of alternative measures"

Additional file 5. PRISMA flowchart detailing systematic search protocol for the study's second objective (Sample 2).

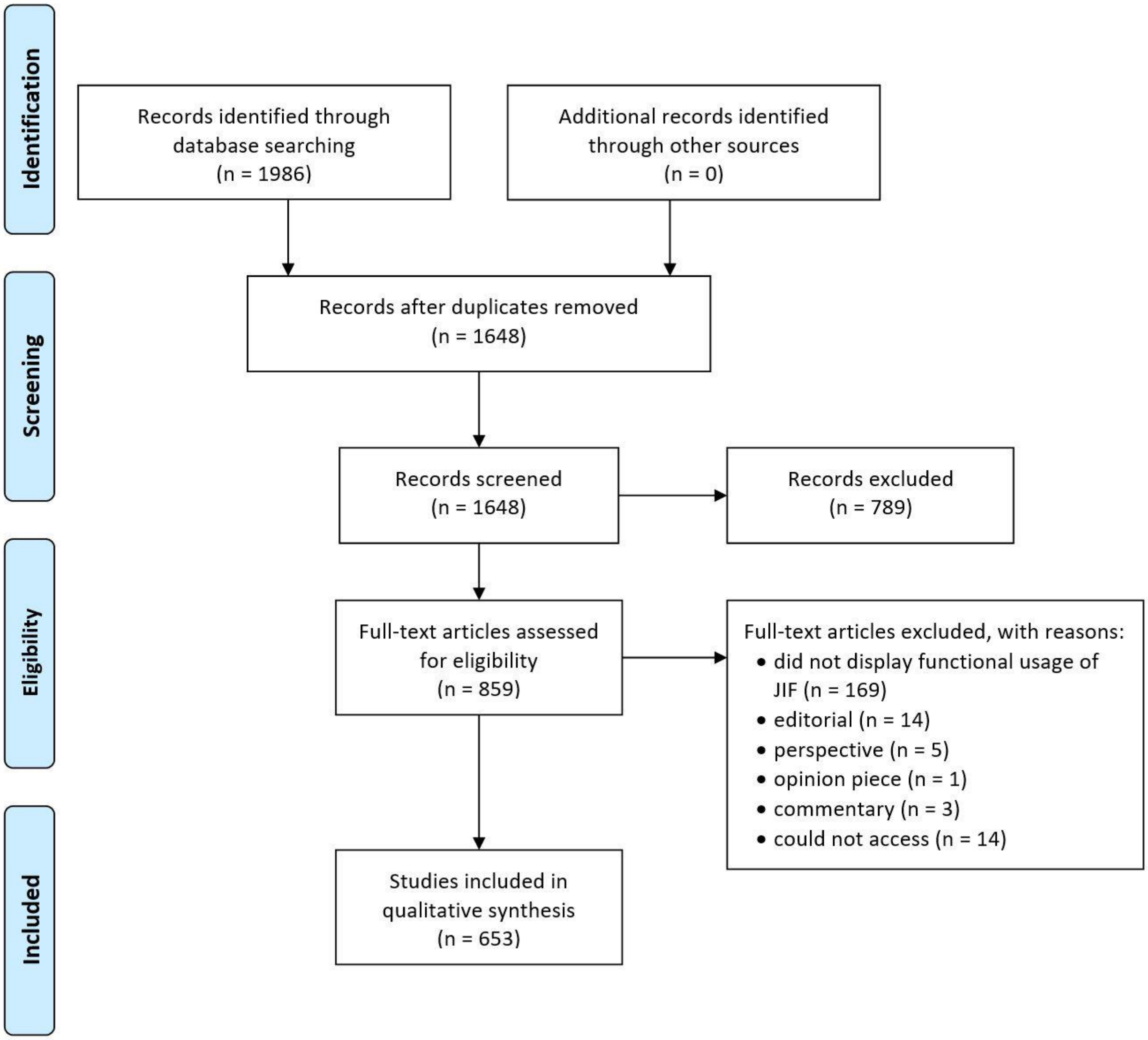

Supplement: Additional file 5. [file 1678-9199-jvatitd-26-e20190082-s5.pdf]
