# Supplementary material for: Evaluating Journal Impact Factor: a systematic survey of the pros and cons, and overview of alternative measures
Source: J Venom Anim Toxins Incl Trop Dis. 2020 Aug 31;26:e20190082. doi: 10.1590/1678-9199-JVATITD-2019-0082 (PMC7458102; doi:10.1590/1678-9199-JVATITD-2019-0082)
Supplement: Additional file 6. [file 1678-9199-jvatitd-26-e20190082-s6.pdf]

Journal of Venomous Animals and Toxins including Tropical Diseases
https://doi.org/10.1590/1678-9199-JVATITD-2019-0082

Supplementary Material to "Evaluating Journal Impact Factor: a systematic survey of the pros and cons, and overview of alternative measures"

Additional file 6. Sample 3 references addressing alternative measures to JIF.

1. Hunt GE, Jackson D, Watson R, Cleary M. A citation analysis of nurse education journals using various bibliometric indicators. Journal of advanced nursing. 2013;69(7):1441–5.
2. Friedberg EC. A closer look at bibliometrics. DNA repair. 2010;9(10):1018–20.
3. Johnston M. A glaring paradox. Genetics. 2015;199(3):637–8.
4. Harzing A-W, van der Wal R. A Google Scholar h-Index for Journals: An Alternative Metric to Measure Journal Impact in Economics and Business. Journal of the American Society for Information Science and Technology. 2009 Jan;60(1):41–46.
5. Hsu W-C, Tsai C-F, Li J-H. A hybrid indicator for journal ranking an example from the field of Health Care Sciences and Services. Online Information Review. 2015;39(7):858–869.
6. Lando T, Bertoli-Barsotti L. A new bibliometric index based on the shape of the citation distribution. PloS one. 2014;9(12):e115962.
7. Bollen J, Van de Sompel H, Hagberg A, Chute R. A principal component analysis of 39 scientific impact measures. PloS one. 2009;4(6):e6022.
8. Li X. A review of the development and application of the Web impact factor. Online Information Review. 2003;27(6):407–417.
9. Lippi G, Borghi L. A short story on how the H-index may change the fate of scientists and scientific publishing. Clinical chemistry and laboratory medicine. 2014;52(2):e1–3.
10. Oosthuizen JC, Fenton JE. Alternatives to the impact factor. The surgeon : journal of the Royal Colleges of Surgeons of Edinburgh and Ireland. 2014;12(5):239–43.
11. Leydesdorff L. Alternatives to the journal impact factor: I3 and the top-10% (or top-25%?) of the most-highly cited papers. Scientometrics. 2012 Aug;92(2, SI):355–365.
12. Elwood TW. Altmetrics, Biased Metrics, and Contentious Metrics. Journal of allied health. 2017;46(1):62.
13. Taylor RLJ. An Incomplete Story Told by a Single Number. Poultry science. 2015;94(9):1995–6.
14. Wagner CS, Leydesdorff L. An Integrated Impact Indicator: A new definition of `Impact' with policy relevance. RESEARCH EVALUATION. 2012 Sep;21(3):183–188.
15. Garcia-Romero A, Santin D, Sicilia G. Another brick in the wall: a new ranking of academic journals in Economics using FDH. Scientometrics. 2016 Apr;107(1):91–101.
16. Frandsen T, Rousseau R. Article impact calculated over arbitrary periods. Journal of the American Society for Information Science and Technology. 2005 Jan;56(1):58–62.
17. Brink PA. Article visibility: journal impact factor and availability of full text in PubMed Central and open access. Cardiovascular journal of Africa. 2013;24(8):295–6.
18. Santangelo GM. Article-level assessment of influence and translation in biomedical research. Molecular biology of the cell. 2017;28(11):1401–1408.
19. Neylon C, Wu S. Article-level metrics and the evolution of scientific impact. PLoS biology. 2009;7(11):e1000242.
20. De Sordi JO, Conejero MA, Meireles M. Bibliometric indicators in the context of regional repositories: proposing the D-index. Scientometrics. 2016 Apr;107(1):235–258.
21. Durieux V, Gevenois PA. Bibliometric indicators: quality measurements of scientific publication. Radiology. 2010;255(2):342–51.
22. Mansour AM, El Mollayess G, Habib R, Arabi A, Medawar WA. Bibliometric trends in ophthalmology 1997-

2009. Indian Journal of Ophthalmology. 2015 Jan;63(1):54–58.
23. Karanatsiou D, Misirlis N, Vlachopoulou M. Bibliometrics and altmetrics literature review Performance indicators and comparison analysis. PERFORMANCE MEASUREMENT AND METRICS. 2017;18(1, SI):16–27.
24. Abbas AM. Bounds and inequalities relating h-index, g-index, e-index and generalized impact factor: an improvement over existing models. PloS one. 2012;7(4):e33699.
25. Eysenbach G. Can tweets predict citations? Metrics of social impact based on Twitter and correlation with traditional metrics of scientific impact. Journal of medical Internet research. 2011;13(4):e123.
26. Finch A. Can we do better than existing author citation metrics? BIOESSAYS. 2010 Sep;32(9):744–747.
27. Hong S-T, Gasparyan AY. Celebrating the Latest Release of the Journal Impact Factors: Thinking Globally, Acting Locally. Journal of Korean medical science. 2015;30(8):999–1000.
28. Dodson MV. Citation analysis: Maintenance of h-index and use of e-index. Biochemical and biophysical research communications. 2009;387(4):625–6.
29. Nigam A, Nigam PK. Citation Index and Impact factor. Indian journal of dermatology, venereology and leprology. 2012;78(4):511–6.
30. Yin C-Y, Aris MJ, Chen X. Combination of Eigenfactor (TM) and h-index to evaluate scientific journals. Scientometrics. 2010 Sep;84(3):639–648.
31. Kulasegarah J, Fenton JE. Comparison of the h index with standard bibliometric indicators to rank influential otolaryngologists in Europe and North America. European archives of oto-rhino-laryngology : official journal of the European Federation of Oto-Rhino-Laryngological Societies (EUFOS) : affiliated with the German Society for Oto-Rhino-Laryngology - Head and Neck Surgery. 2010;267(3):455–8.
32. Moed HF. Comprehensive indicator comparisons intelligible to non-experts: the case of two SNIP versions. Scientometrics. 2016 Jan;106(1):51–65.
33. Van Noorden R. Controversial impact factor gets a heavyweight rival. Nature. 2016;540(7633):325–326.
34. Elkins MR, Maher CG, Herbert RD, Moseley AM, Sherrington C. Correlation between the Journal Impact Factor and three other journal citation indices. Scientometrics. 2010 Oct;85(1):81–93.
35. Stallings J, Vance E, Yang J, Vannier MW, Liang J, Pang L, et al. Determining scientific impact using a collaboration index. Proceedings of the National Academy of Sciences of the United States of America. 2013;110(24):9680–5.
36. Bornmann L, Marx W, Gasparyan AY, Kitas GD. Diversity, value and limitations of the journal impact factor and alternative metrics. RHEUMATOLOGY INTERNATIONAL. 2012 Jul;32(7):1861–1867.
37. Jackson D, Haigh C, Watson R. Editorial: Nurses and publications - the impact of the impact factor. Journal of clinical nursing. 2009;18(18):2537–8.
38. Hunt GE, Cleary M. Editorial: Quality is better than quantity when it comes to publications. Journal of clinical nursing. 2011;20(1–2):70–2.
39. Jacso P. Eigenfactor and article influence scores in the Journal Citation Reports. Online Information Review. 2010;34(2):339–348.
40. Mingers J, Yang L. Evaluating journal quality: A review of journal citation indicators, and ranking in business and management. EUROPEAN JOURNAL OF OPERATIONAL RESEARCH. 2017 Feb;257(1):323–337.
41. Herrmann-Lingen C, Brunner E, Hildenbrand S, Loew TH, Raupach T, Spies C, et al. Evaluation of medical research performance–position paper of the Association of the Scientific Medical Societies in Germany (AWMF). German medical science : GMS e-journal. 2014;12(101227686):Doc11.
42. Sebire NJ. H-index and impact factors: assessing the clinical impact of researchers and specialist journals. Ultrasound in obstetrics & gynecology : the official journal of the International Society of Ultrasound in Obstetrics and Gynecology. 2008;32(7):843–5.
43. Mullins ME. Has the time come for bibliometrics and the h-index in academic radiology?. Academic radiology. 2010;17(7):815–6.
44. Bornmann L, Marx W, Schier H. Hirsch-Type Index Values for Organic Chemistry Journals: A Comparison of New Metrics with the Journal Impact Factor. EUROPEAN JOURNAL OF ORGANIC CHEMISTRY. 2009 Apr;(10):1471–1476.
45. Gefen A. How high is a “high” Hirsch index in biomechanics research?. Journal of biomechanics. 2011;44(1):206–9.
46. Scully C. Impact and other newer factors. Oral oncology. 2009;45(12):1005.

47. Rocha-e-Silva M. Impact factor, Scimago Indexes and the Brazilian journal rating system: where do we go from here? Clinics (Sao Paulo, Brazil). 2010;65(4):351–5.
48. Kamath PS, Bologna G. Impact factor: misused and overhyped?. Hepatology (Baltimore, Md). 2009;49(6):1787–9.
49. Miller CS. Impact versus impact factor and Eigenfactor. Oral surgery, oral medicine, oral pathology and oral radiology. 2012;113(2):145–6.
50. Conn VS, Chan KC. Moving beyond counting publications to assess impact. Western journal of nursing research. 2015;37(3):283–7.
51. Andersson A, Borjesson JL. Operating in an era of impact factor mania. Upsala journal of medical sciences. 2015;120(2):124–31.
52. Roberts WC. Piercing the impact factor and promoting the EigenfactorTM. The American journal of cardiology. 2011;108(6):896–8.
53. Ward J. Prestige versus citation volume as journal indices in cognitive neuroscience. Cognitive neuroscience. 2014;5(3–4):135–7.
54. Flaatten H, Rasmussen LS, Haney M. Publication footprints and pitfalls of bibliometry. Acta anaesthesiologica Scandinavica. 2016;60(1):3–5.
55. Smith DR. Quantum leap in the AEOH impact factor. Archives of environmental & occupational health. 2010;65(3):119–20.
56. Fang FC, Casadevall A. Retracted Science and the Retraction Index. INFECTION AND IMMUNITY. 2011 Oct;79(10):3855–3859.
57. Kali A. Scientific impact and altmetrics. Indian journal of pharmacology. 2015;47(5):570–1.
58. Honekopp J, Kleber J. Sometimes the impact factor outshines the H index. RETROVIROLOGY. 2008 Oct;5.
59. Aoun SG, Bendok BR, Rahme RJ, Dacey RG Jr, Batjer HH. Standardizing the Evaluation of Scientific and Academic Performance in Neurosurgery-Critical Review of the “h” Index and its Variants. WORLD NEUROSURGERY. 2013 Nov;80(5):E85–E90.
60. Sillet A, Katsahian S, Range H, Czernichow S, Bouchard P. The Eigenfactor (TM) Score in Highly Specific Medical Fields: The Dental Model. JOURNAL OF DENTAL RESEARCH. 2012 Apr;91(4):329–333.
61. Wykes T, Lipczynska S, Guha M. The h-index, the citation rating, impact factors and the aspiring researcher. Journal of mental health (Abingdon, England). 2013;22(6):467–73.
62. Wilms G. The impact factor. Neuroradiology. 2013;55(7):803–6.
63. Fersht A. The most influential journals: Impact Factor and Eigenfactor. Proceedings of the National Academy of Sciences of the United States of America. 2009;106(17):6883–4.
64. Wurtz M, Schmidt M. The stratified H-index. ANNALS OF EPIDEMIOLOGY. 2016 Apr;26(4):299–300.
65. Haeffner-Cavaillon N, Graillot-Gak C. The use of bibliometric indicators to help peer-review assessment. Archivum immunologiae et therapiae experimentalis. 2009;57(1):33–8.
